# RawBeans: A Simple, Vendor-Independent, Raw-Data Quality-Control Tool

**DOI:** 10.1021/acs.jproteome.0c00956

**Published:** 2021-03-04

**Authors:** David Morgenstern, Rotem Barzilay, Yishai Levin

**Affiliations:** † de Botton Institute for Protein Profiling, The Nancy and Stephen Grand Israel National Center for Personalized Medicine, Weizmann Institute of Science, Rehovot 76100, Israel; ‡ Ilana and Pascal Mantoux Institute for Bioinformatics, The Nancy and Stephen Grand Israel National Center for Personalized Medicine, Weizmann Institute of Science, Rehovot 76100, Israel

**Keywords:** QC, quality control, nanoLC-MS/MS, Raw Data

## Abstract

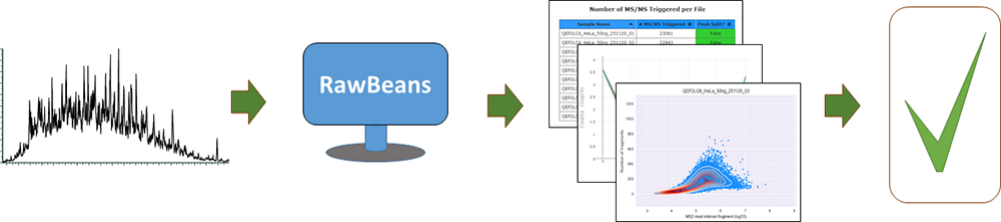

Every laboratory performing mass-spectrometry-based proteomics strives to generate high-quality data. Among the many factors that impact the outcome of any experiment in proteomics is the LC–MS system performance, which should be monitored within each specific experiment and also long term. This process is termed quality control (QC). We present an easy-to-use tool that rapidly produces a visual, HTML-based report that includes the key parameters needed to monitor the LC–MS system performance, with a focus on monitoring the performance within an experiment. The tool, named RawBeans, generates a report for individual files or for a set of samples from a whole experiment. We anticipate that it will help proteomics users and experts evaluate raw data quality independent of data processing. The tool is available at https://bitbucket.org/incpm/prot-qc/downloads. The mass-spectrometry proteomics data have been deposited to the ProteomeXchange Consortium via the PRIDE partner repository with the data set identifier PXD022816.

## Introduction

Mass-spectrometry-based proteomics is an essential technique in life sciences, enabling analyses of whole proteomes, posttranslational modifications, protein–protein interactions, and more. The same flexibility that allows the application of the technology to a myriad of proteomics experiments also makes it very challenging to achieve a high degree of repeatability and reproducibility due to the technical limitations of instruments,^2−4^ variations in sample preparation,^5−8^ data processing,^9−11^ LC column degradation,^12^ loss of sensitivity due to long-term effects, and chemical modifications in the LC autosampler vials.^13^ All have detrimental effects on the data quality. As a result of this potential variability, a call for quality-control (QC) measures has been made.^14−16^

QC in proteomics is directed toward sample preparation, chromatography, data acquisition (i.e., performance of the LC and MS), and data analysis. Toward that end, Rudnick et al. generated a comprehensive list of performance metrics that should be taken into account in the QC analyses of generated data.^16^ This was followed by development of many software tools and script pipelines aiming to provide users QC information on the performance of their instruments.

Two of the earliest tools were RawMeat by Vast Scientific, which is no longer supported, and LogViewer,^17^ which requires prepreparation of the data before analysis. Both tools provide quick, identification-free, graphic information about the instrument performance and are simple to use, which is why they were popular; however, they are limited to Thermo instruments and are not updated to handle the latest instrumentation.

MSQC is a software developed with the QC criteria determined by Rudnick,^16^ producing data for 46 metrics described in the manuscript. Unfortunately, this tool requires database search results for most metrics, is dependent on a search engine, does not accept the generic search results format, mzID,^18^ from completed search results, involves multiple format conversions, and lacks clear and easy visualizations. Lastly, MSQC is no longer supported. QuaMeter^19^ circumvents some the issues with MSQC by using a generic format for spectral data, mzML,^20^ and an identifications format (mzID), allowing an independent analysis of data from any vendor and any search engine. Moreover, this tool can provide identification-independent metrics, allowing QC before the data-processing pipeline is concluded. Unfortunately, the output is in the form of the Tab-delimited format and requires downstream analysis to extract important information on the instrument’s performance.

Newer server- and database-based pipelines, allow data archiving and time-course accumulation of the data. These are either identification-dependent^19,21^ or require the use of known peptides either in separate QC runs or spiked into the samples.^22^ Both of these tools allow local implementation and are easy to use, and Metriculator,^23^ is also open source, allowing users to modify it based on their needs. The last pipeline worth noting is SIMPATIQCO.^24^ This is a server-based tool that allows large laboratories to accumulate all of the relevant data from all of the instruments in the lab, store it, and analyze it automatically. This tool provides both identification-dependent and -independent metrics. It is a powerful tool, but it is not intuitive and requires a certain degree of expertise to install and operate.

The popularity of RawMeat as a QC tool, despite its limitations, shows the need for an up-to-date identification-free tool that is simple, intuitive, and graphic. Here we present **RawBeans**, a vendor-independent tool for QC of raw data. The input can be Thermo data, mzML, or other vendor data that are converted into mzML by integration of msconvert from ProteoWizard.

The tool can be run standalone on a Windows PC or can be automated as part of a pipeline (https://bitbucket.org/incpm/prot-qc/downloads/).

The focus of this tool is evaluation of a set of raw data files from one experiment or sporadic raw files, as it produces a graphical representation of the key raw data parameters, which can be reviewed quickly prior to data processing or to assist in troubleshooting efforts. For long-term evaluation of instrument performance, it requires regeneration of areport with every new raw file added, which makes it less practical for this purpose.

To show how RawBeans can be of use, we generated a set of 10 repeated analyses of a 50 ng HeLa digest, where some of the injections included problems we introduced, to mimic real-life scenarios. For one injection, we set the normalized collision energy (NCE) to 10, compared with 27, which is the optimal value. This generated suboptimal fragmentation. We also changed the injection volume from 1 to 0.5 and 2 μL for two of the injections, representing differences in sample loading or changes in sensitivity during the experiment. Finally, for one of the samples, we set the spray voltage to zero during the run, simulating a drop in spray that sometimes occurs when using nanoflow LC.

We also provide RawBeans reports generated from ABSciex Q-ToF data and Bruker TIMS-ToF data to exemplify its ability to analyze multiple vendor data (Supplementary Files S3 and S4).

Taken together, it exemplifies the utility of our tool in real-life settings.

## Materials and Methods

A HeLa digestion standard (Pierce Thermo, USA) was solubilized with 97:3 H_2_O/ACN + 0.1% formic acid and diluted to 50 ng/μL.

ULC/MS-grade solvents were used for all chromatographic steps. Each sample was loaded using splitless nano-ultra-performance liquid chromatography (10 kpsi nanoAcquity; Waters, Milford, MA). The mobile phase was: (A) H_2_O + 0.1% formic acid and (B) acetonitrile + 0.1% formic acid. Desalting of the samples was performed online using a reversed-phase symmetry C18 trapping column (180 μm internal diameter, 20 mm length, 5 μm particle size; Waters). The peptides were then separated using a T3 HSS nanocolumn (75 μm internal diameter, 250 mm length, 1.8 μm particle size; Waters) at 0.35 μL/min. Peptides were eluted from the column into the mass spectrometer using the following gradient: 5–35% B in 50 min, 35–90% B in 5 min, maintained at 90% for 5 min, and then back to initial conditions.

The nanoUPLC was coupled online through a nanoESI emitter (10 μm tip; New Objective, Woburn, MA) to a quadrupole-Orbitrap mass spectrometer (Q Exactive Plus, Thermo Scientific) using a FlexIon nanospray apparatus (Proxeon).

Data were acquired in data-dependent acquisition (DDA) mode using a Top10 method. The MS1 resolution was set to 70 000 with a mass range of 375–1650 *m*/*z* and an automatic gain control (AGC) of 3e6, and the maximum injection time was set to 60 ms. The MS2 resolution was set to 17 500 with quadrupole isolation of 1.7 *m*/*z*, an AGC of 1e5, dynamic exclusion of 30 s, and a maximum injection time of 60 ms.

The mass-spectrometry proteomics data have been deposited to the ProteomeXchange Consortium via the PRIDE^1^ partner repository with the data set identifier PXD022816.

Raw data were imported into RawBeans version 1.5.1 using the default parameters (https://bitbucket.org/incpm/prot-qc/downloads/).

## Results

RawBeans was designed with the main aim of producing an easy-to-use, accessible, visual tool for raw data QC. The idea is to be able to quickly spot a problematic run and have it stand out compared with other raw files within a given experiment or to aid in pinpointing the source of a problem during a troubleshooting process. The input to RawBeans can be .raw files (Thermo Scientific) or the generic mzML, or the data can be converted to mzML using msconvert (ProteoWizard), which is embedded into the tool. The data can be of data-dependent acquisition (DDA) type or data-independent acquisition (DIA) type for Thermo data only (Supplementary File S5).

RawBeans can be used to generate individual reports or one report for a set of samples.

The output is an HTML-based report that includes the information listed in Table 1. This makes up the essential information that can be extracted directly from raw mass-spectrometry data.

**Table 1:** List of the Tabs in the RawBeans HTML Report and a Brief Description of Each Tab

tab name	explanation
MS2 counts	Number of triggered MS/MS spectra per raw file. It includes a test for peak splitting (tribrid instruments only).
Top-N	Number of triggered MS/MS events per data-dependent cycle, shown as histograms in Log10 scale.
Charge distribution	Histograms of the precursor charge state based on the triggered MS/MS events.
Injection Time	Histograms of MS/MS injection time in Log10 scale.
Retention Time vs TopN	Graphs of the number of MS/MS events per data-dependent cycle versus the retention time per raw file.
Injection vs Retention	MS1 injection time per retention time for each raw file.
Total Ion Current	Sum of MS1 signals per raw file based on all full MS1 scans. Presented in linear scale.
MS2-intensities	Graphs of the number of fragment ions versus the most intense fragment ion (in log10 scale).
MS2 Precursor Ratio	Binned ratios of the precursor ion to the next highest fragment ion intensity per MS/MS scan.
Triggered M/Z distribution	Density plots of the precursor *m*/*z* based on the triggered MS/MS events.
FWHM	Chromatographic full width at half-height. A crude measurement of peak width.
Peak Symmetry	Chromatographic peak symmetry. A crude measurement to show global peak tailing.
Mass Deviation	Mass error throughout the run time based on the masses entered in the GUI.

## Quality Control and Troubleshooting

The quality of MS-based proteomic data relies on instrument performance. The better the performance, the better the data. Performance is a general term that encompasses a number of key components of the instrument: electrospray stability, cleanliness of the ion source, cleanliness of the inner components of the MS, fragmentation efficiency, and mass accuracy. If any one of these is not working at optimal conditions, they will have a negative effect on data quality. Spotting these problems in an early stage may help one to avoid unsuccessful experiments, thus saving costly MS time as well as precious samples.

To show how RawBeans can help in the quality-control process, we performed repeated injections of 50 ng of HeLa cell digest. We introduced three problems: spray instability, unequal sample loading, and a problem with fragmentation. The report is provided as Supplementary File S1.

## Visualizing Total Signal

**Figure 1:**
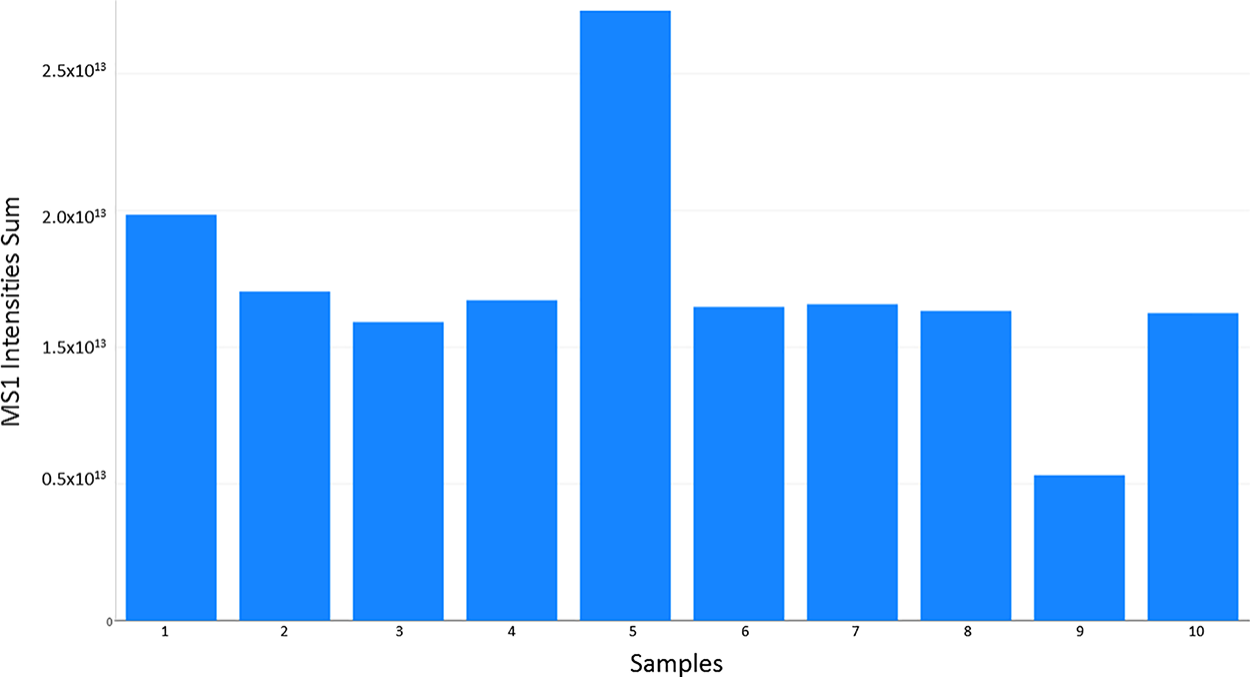
Bar graph showing the total signal of all peaks in all MS1 scans of each sample. In the HTML report, the samples can be ordered according to their name or according to the running order.

In label-free quantification, one typically expects similar overall signals from the samples. Variations in the overall signal can be due to a drop in sensitivity during the experiment or unequal sample loading. RawBeans shows the user if there are variations in the total signal by the graph called “Total Ion Current”. This is the sum of all peak intensities in all MS1 scans in the data. It can be seen in Figure 1 that samples 05 and 09 stand out. Sample 05 was 100 ng loading, and sample 09 was 25 ng loading. The bar plot in Figure 1 is in correlation with these loadings.

## Fragmentation Efficiency

Fragmentation in MS/MS scans is essential for identification of the molecules being analyzed (e.g. peptides in bottom up proteomics). To test how RawBeans can be utilized to identify problems with fragmentation, we set the normalized collision energy (NCE) to 10 instead of 27. At this value, we expect to primarily detect the precursor ion, with very low-intensity fragment ions in the MS/MS spectra.

**Figure 2:**
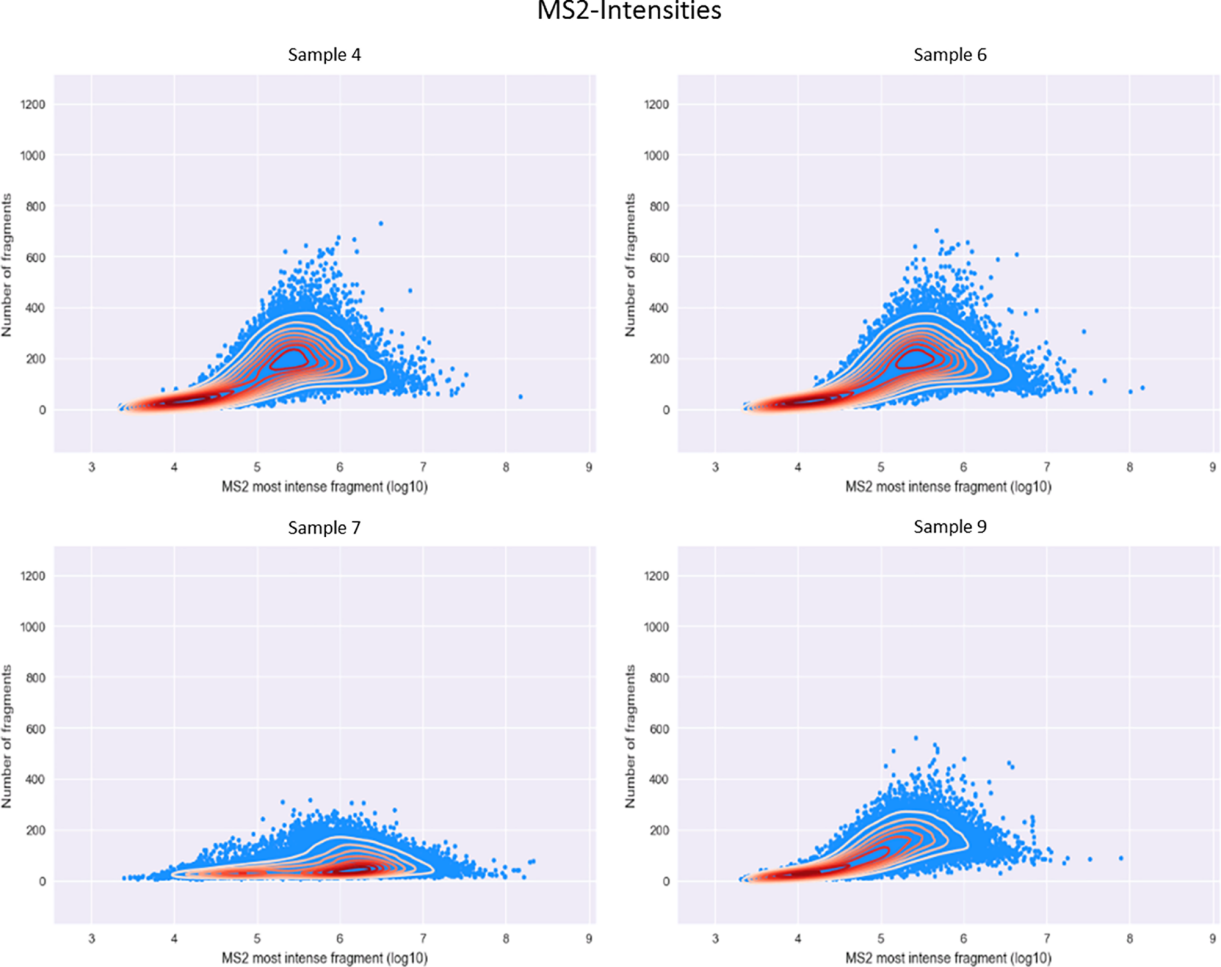
Intensity of the most intense peak in a given MS/MS scan in log scale (*x* axis) versus the number of fragment ions in the MS/MS scan (*y* axis). The yellow to red color shows areas of high density. A graph is generated for each sample. Here we show four of the ten graphs.

Looking at the tab “MS2-intensities” of the RawBeans report, we can see that sample 7 stands out compared with the rest. Figure 2 shows the plots of four of the ten injections. “MS2-intensities” is a measure of how efficient the fragmentation is. Each point on the plot represents a single MS/MS, where the *x* axis value represents the log-transformed intensity of the most intense fragment and the *y*-axis value represents the number of fragments in the MS2 spectrum. The main difference in sample 7 is in the *y* axis, which shows the number of fragment ions in each MS/MS scan. Furthermore, we can see that the density of the most intense peaks is higher compared with that of other samples, at just over 6E10. This is most likely due to the high intensity of the precursor ion, which was unfragmented in most cases. It is also worth noting that sample 9 is also slightly lower on the *y* axis, and this is due to the fact that in this sample, we injected 25 ng instead of 50 ng, and thus generally, we get lower intensity precursors translating to fewer fragment ions.

**Figure 3:**
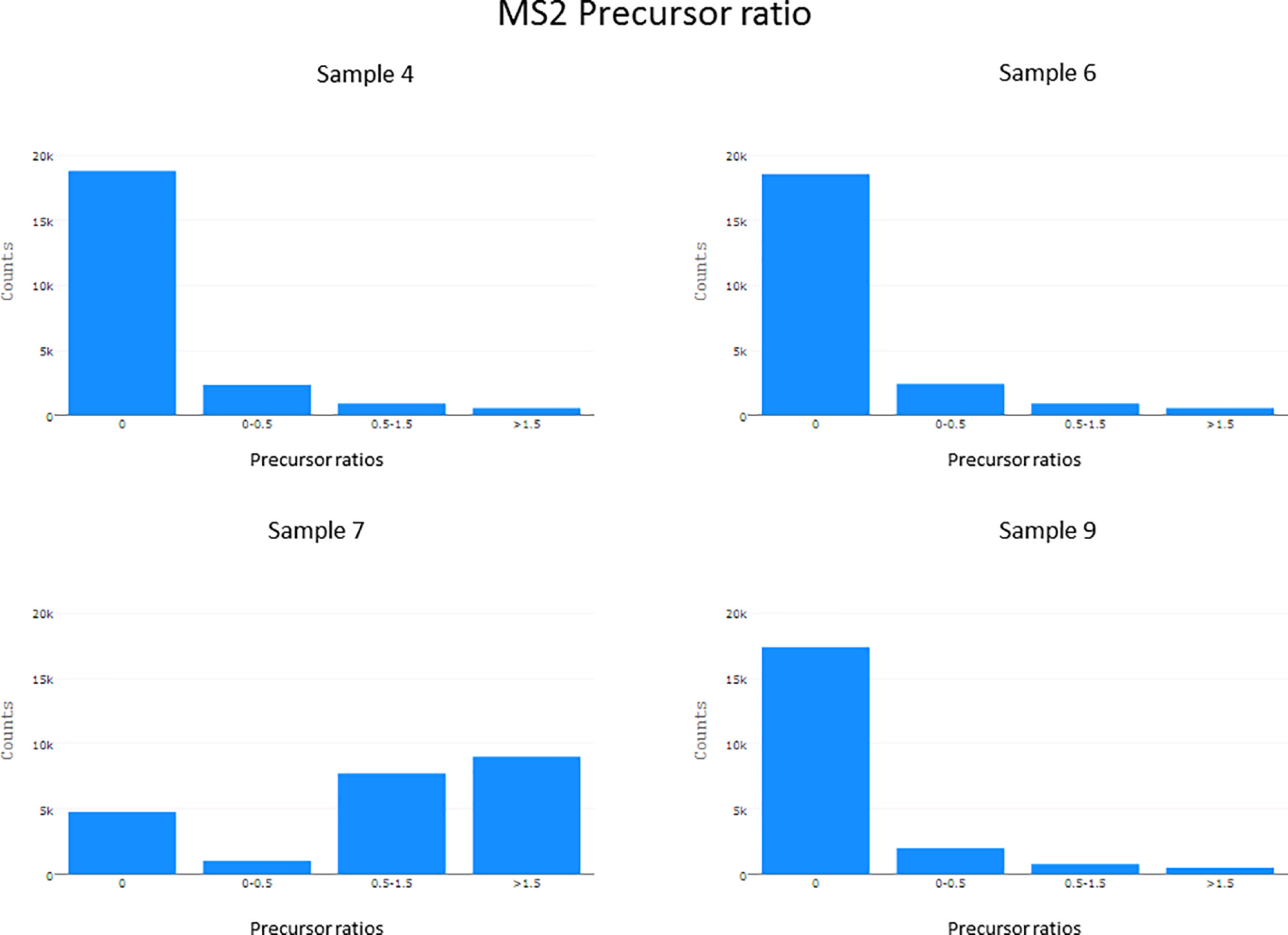
Binned bar graphs showing the ratio of the precursor intensity to the next highest fragment ion in an MS/MS scan. A graph is generated for each sample.

Another piece of information that helps to identify problems related to fragmentation is the “MS2 Precursor Ratio” tab. Here RawBeans calculates the ratio of the precursor intensity to the next highest fragment ion intensity in a given MS/MS spectrum. This is shown as a binned bar graph. When fragmentation is efficient in most MS/MS spectra, the highest bar is that for a ratio of zero. However, when fragmentation is not efficient, like in sample 7, we expect a shift toward ratios that are greater than zero (Figure 3).

## Spray Stability

The most common ionization setup in mass-spectrometry-based proteomics is nanoESI. In this setup, achieving spray stability is sometimes challenging. When the spray is unstable, it often causes decreased sensitivity for a short period of time, resulting in almost no signal for a few seconds to a few minutes during an LC–MS run. Whereas these signal dropouts can be easily spotted by looking at the raw data file in the acquisition software, in a large data set, this means having to open each raw file one by one, which is inconvenient and might take a long time.

**Figure 4:**
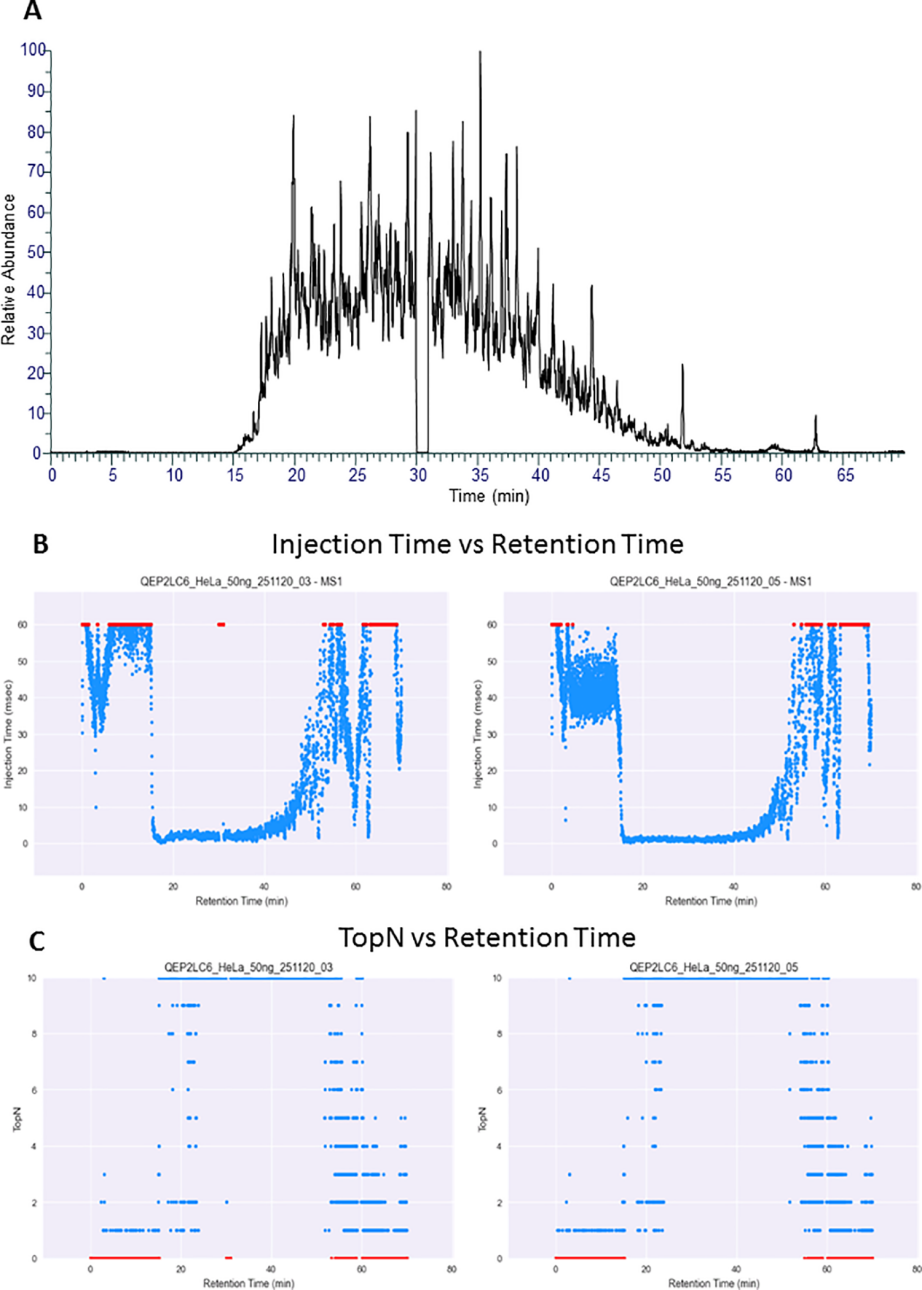
(A) Chromatogram of sample 3, where we introduced zero spray voltage to simulate spray instability at 30 min for 1 min. (B) “Injection Time vs Retention Time” view from the RawBeans report. One can see the red dots at time 30 min of sample 3, indicating a brief drop in signal that, in turn, increases the injection time to the maximum. (C) “TopN vs Retention Time” view. One can see the red dots at 30 min at zero values.

When generating the RawBeans report, one can quickly inspect all files in an experiment and spot problematic files. To show this, we created a signal dropout in sample 3 of our experiment. We set the spray voltage to zero for 1 min in the middle of the run. Figure 4 shows the resulting chromatogram and two views from the report. One, “Injection Time vs Retention Time”, shows that at the time of the signal drop due to zero spray voltage, the injection time increases to the maximum. This is indicated by the red dots at 30 min. The second view where this can be seen is in “Retention Time vs TopN”. Here one can see a drop in TopN at 30 min.

## Long-Term Monitoring

RawBeans can also be used for long-term performance monitoring, although it is somewhat cumbersome for this purpose because with every new raw file, the report needs to be generated again and again. Nevertheless, to exemplify this capability, we generated a report from 86 QC raw files run throughout several months. These files were 100 ng of HeLa digest that were run periodically to test the instrument performance. The report is provided as Supplemental File S2.

## Vendor Independency

RawBeans can accept raw Thermo data directly and also data from other vendors, which is then converted using the embedded MSCONVERT (ProteoWizard). When generating a report for other data formats, mainly Q-ToF instruments, some of the graphs are not generated, such as the Injection Time. However, it still provides useful information, as can be seen in Supplemental Files S3 (ABSciex data) and S4 (Bruker, TIMS-ToF Pro data).

## Discussion

We developed an easy-to-use tool that provides information regarding the quality of the mass-spectrometry data postacquisition. The information is presented in an HTML-based report and allows a user to rapidly inspect one or several raw data files independent of any data processing. The tool can be used as a standalone executable or as a command line for automation by software engineers.

We presented three scenarios where RawBeans can be useful to pinpoint problems and assist in troubleshooting. We generated ten nanoLC–MS/MS analyses, acquired in DDA mode, where we introduced three example problems: spray drop and different sample loadings and fragmentation issues. There are many other potential problems that the tool can aid in identifying, such as peak splitting, sensitivity problems, incorrect acquisition parameters, chromatographic peak broadening, and many others. We provide the tool as a courtesy to the mass-spectrometry community in the hope that we all generate high-quality data.

## Supporting Information Available

File S1. RawBeans report of 10 HeLa runs (ZIP)
File S2. RawBeans report of 86 HeLa QC runs from one instrument (ZIP)
File S3. RawBeans report of ABSciex data from ProteomeXchange PXD023653 (ZIP)
File S4. RawBeans report of Bruker TIMS-ToF data from ProteomeXchange PXD021832 (ZIP)
File S5. RawBeans report of a DIA raw data (ZIP)

## Notes

The tool is available at https://bitbucket.org/incpm/prot-qc/downloads. The mass-spectrometry proteomics data have been deposited to the ProteomeXchange Consortium via the PRIDE partner repository with the data set identifier PXD022816.

## Abbreviations

nanoLC: nanoflow liquid chromatography
QC: quality control
