# Supplementary material for: RawBeans: A Simple, Vendor-Independent, Raw-Data Quality-Control Tool
Source: J Proteome Res. 2021 Mar 4;20(4):2098–104. doi: 10.1021/acs.jproteome.0c00956 (PMC8041395; doi:10.1021/acs.jproteome.0c00956)
Supplement: Supplementary file 1 — pr0c00956_si_002.zip [file pr0c00956_si_002.zip › RawBeans_report/qc-report.html]

QC Report


# Raw Beans

MS2 Counts
Top-N
Charge Distribution
Injection Time
Retention vs. Top-N
Injection vs. Retention
Total Ion Current
MS2-Intensities
MS2 Precursor Ratio
Triggered M/Z Distribution
FWHM
Peak Symmetry
Mass Deviation
Help

## Number of MS/MS Triggered per File

| Sample Name | # MS/MS Triggered | Peak Split? |
| --- | --- | --- |
| QEP2LC6\_HeLa\_50ng\_251120\_03 | 22116 | False |
| QEP2LC6\_HeLa\_50ng\_251120\_10 | 21884 | False |
| QEP2LC6\_HeLa\_50ng\_251120\_02 | 22843 | False |
| QEP2LC6\_HeLa\_50ng\_251120\_07 | 22566 | False |
| QEP2LC6\_HeLa\_50ng\_251120\_05 | 23660 | False |
| QEP2LC6\_HeLa\_50ng\_251120\_06 | 22451 | False |
| QEP2LC6\_HeLa\_50ng\_251120\_04 | 22651 | False |
| QEP2LC6\_HeLa\_50ng\_251120\_09 | 20733 | False |
| QEP2LC6\_HeLa\_50ng\_251120\_01 | 23081 | False |
| QEP2LC6\_HeLa\_50ng\_251120\_08 | 22158 | False |

## TopN per Cycle

## Charge Distributions

## Injection Time

## Retention Time vs TopN

## Injection Time vs Retention Time

## Total Ion Current

Sort by Name

## MS2-Intensities

## MS2 Precursor Ratio

## Triggered M/Z Distribution

## FMHW

## Peak Symmetry

## Mass Deviation
