# Supplementary figures and images for: RawBeans: A Simple, Vendor-Independent, Raw-Data Quality-Control Tool (part 2 of 3)
Source: J Proteome Res. 2021 Mar 4;20(4):2098–104. doi: 10.1021/acs.jproteome.0c00956 (PMC8041395; doi:10.1021/acs.jproteome.0c00956)

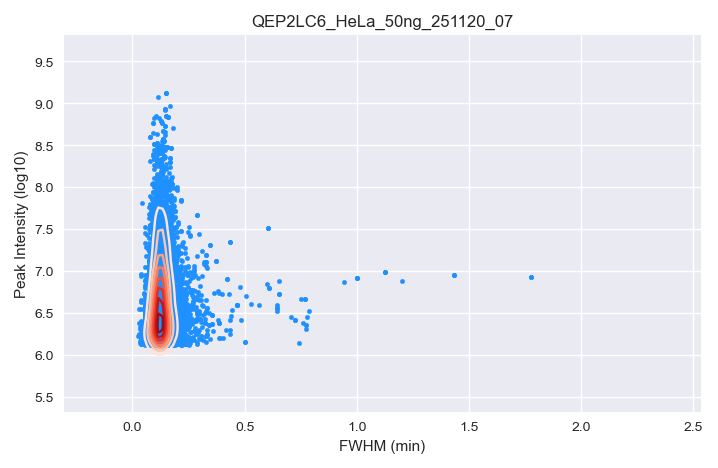

Supplement: Supplementary file 1 — pr0c00956_si_002.zip [file pr0c00956_si_002.zip › RawBeans_report/resources/images/QEP2LC6_HeLa_50ng_251120_07-peak-intentsity-vs-t-sum.png]

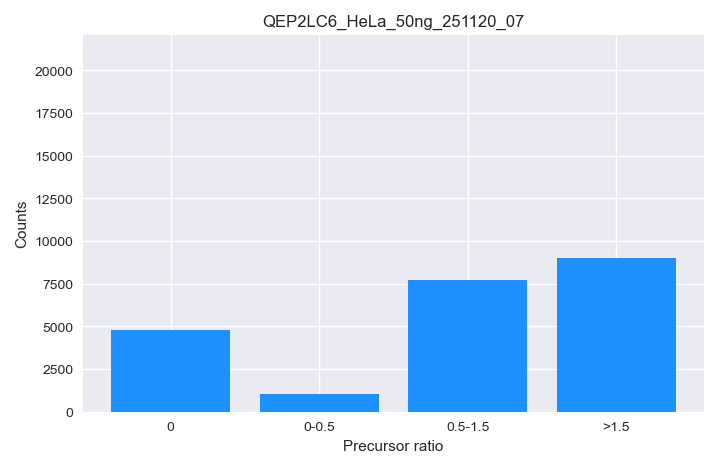

Supplement: Supplementary file 1 — pr0c00956_si_002.zip [file pr0c00956_si_002.zip › RawBeans_report/resources/images/QEP2LC6_HeLa_50ng_251120_07-prec-ratio.png]

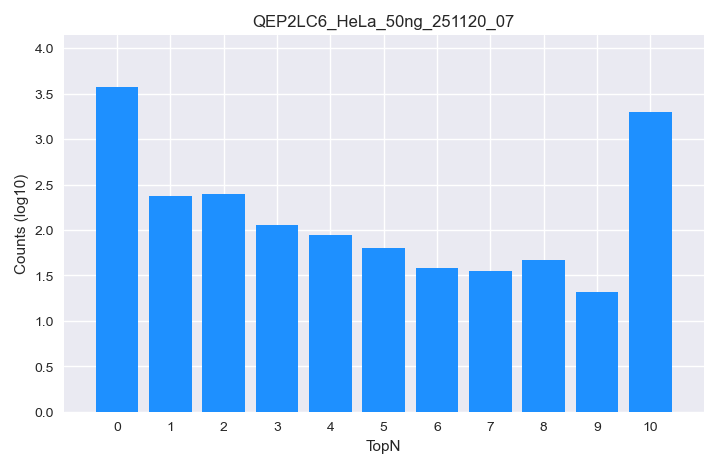

Supplement: Supplementary file 1 — pr0c00956_si_002.zip [file pr0c00956_si_002.zip › RawBeans_report/resources/images/QEP2LC6_HeLa_50ng_251120_07-top-n.png]

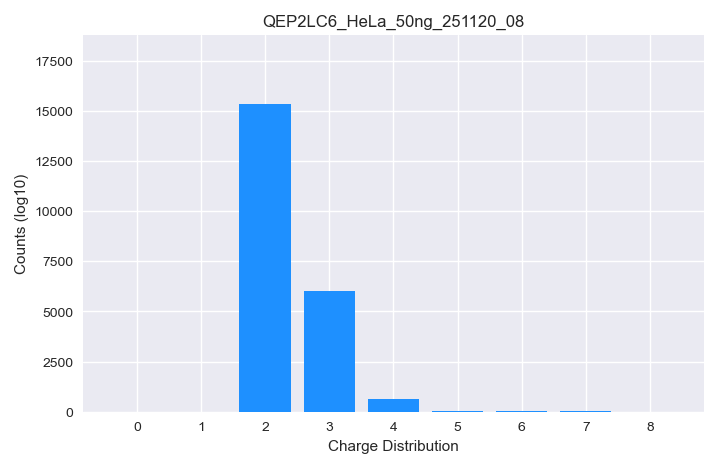

Supplement: Supplementary file 1 — pr0c00956_si_002.zip [file pr0c00956_si_002.zip › RawBeans_report/resources/images/QEP2LC6_HeLa_50ng_251120_08-charge-state.png]

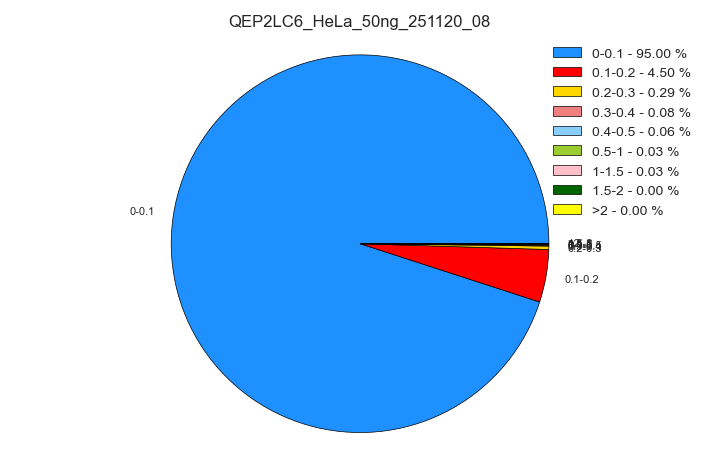

Supplement: Supplementary file 1 — pr0c00956_si_002.zip [file pr0c00956_si_002.zip › RawBeans_report/resources/images/QEP2LC6_HeLa_50ng_251120_08-fmhw-pie.png]

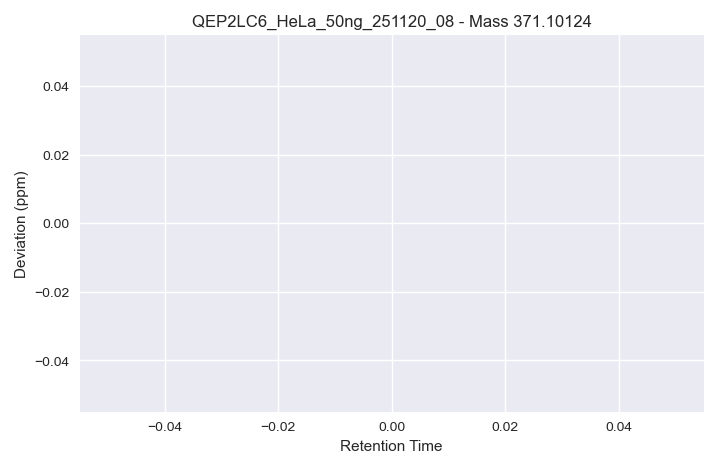

Supplement: Supplementary file 1 — pr0c00956_si_002.zip [file pr0c00956_si_002.zip › RawBeans_report/resources/images/QEP2LC6_HeLa_50ng_251120_08-mass-deviation1.png]

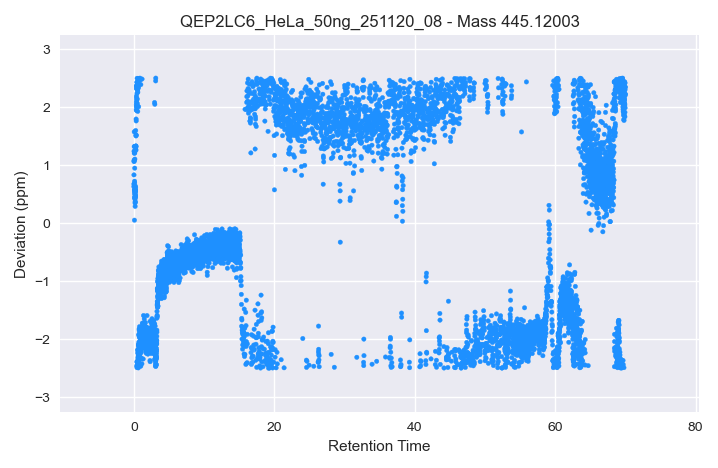

Supplement: Supplementary file 1 — pr0c00956_si_002.zip [file pr0c00956_si_002.zip › RawBeans_report/resources/images/QEP2LC6_HeLa_50ng_251120_08-mass-deviation2.png]

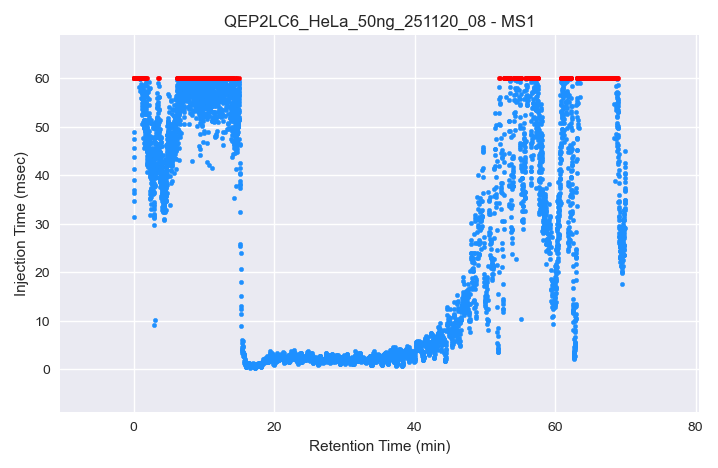

Supplement: Supplementary file 1 — pr0c00956_si_002.zip [file pr0c00956_si_002.zip › RawBeans_report/resources/images/QEP2LC6_HeLa_50ng_251120_08-ms1-inject-vs-ret.png]

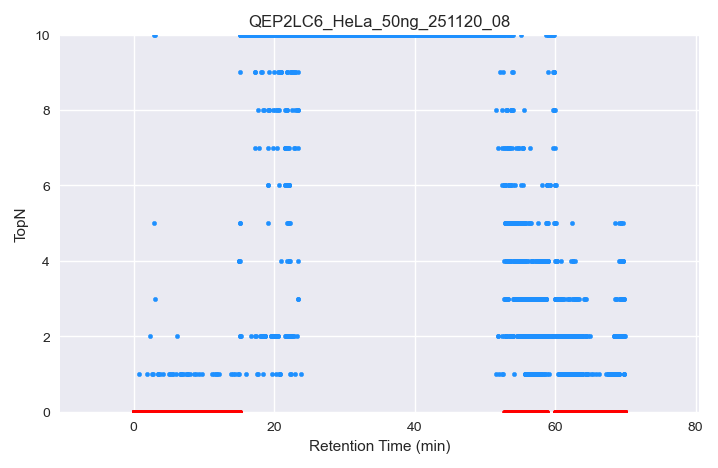

Supplement: Supplementary file 1 — pr0c00956_si_002.zip [file pr0c00956_si_002.zip › RawBeans_report/resources/images/QEP2LC6_HeLa_50ng_251120_08-ms1-ret-vs-top-n.png]

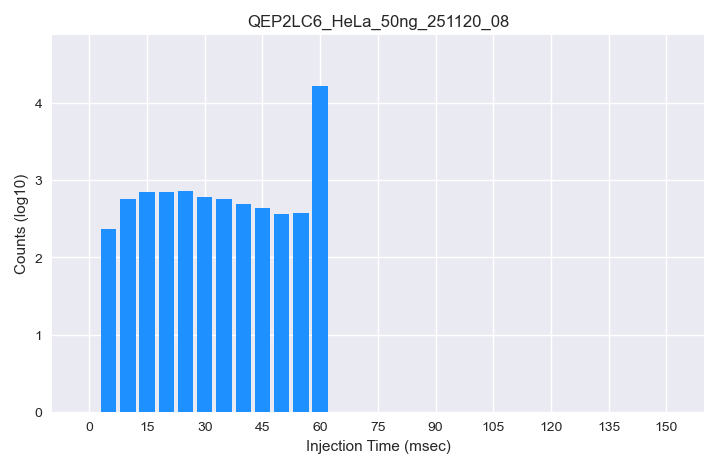

Supplement: Supplementary file 1 — pr0c00956_si_002.zip [file pr0c00956_si_002.zip › RawBeans_report/resources/images/QEP2LC6_HeLa_50ng_251120_08-ms2-inject.png]

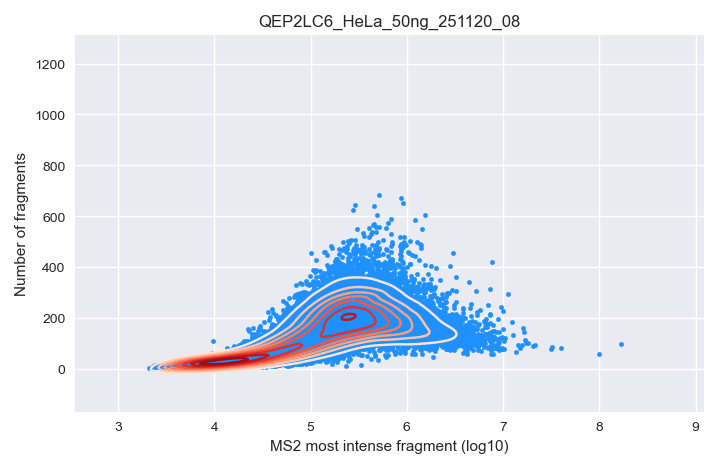

Supplement: Supplementary file 1 — pr0c00956_si_002.zip [file pr0c00956_si_002.zip › RawBeans_report/resources/images/QEP2LC6_HeLa_50ng_251120_08-ms2-max-log-intensity-vs-ms2-num-intensities.png]

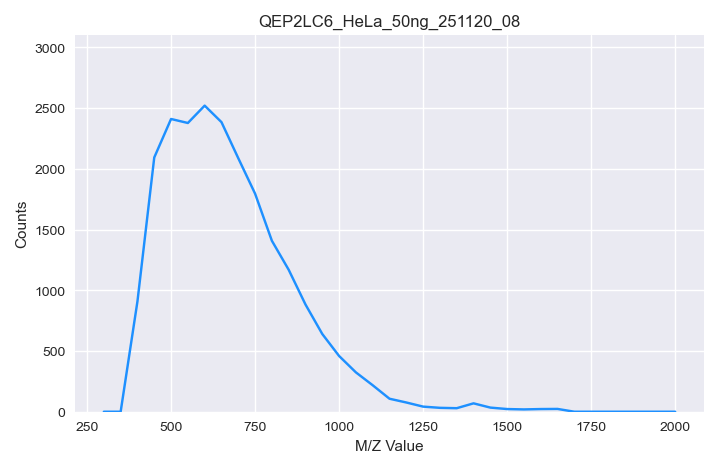

Supplement: Supplementary file 1 — pr0c00956_si_002.zip [file pr0c00956_si_002.zip › RawBeans_report/resources/images/QEP2LC6_HeLa_50ng_251120_08-ms2-mz-value.png]

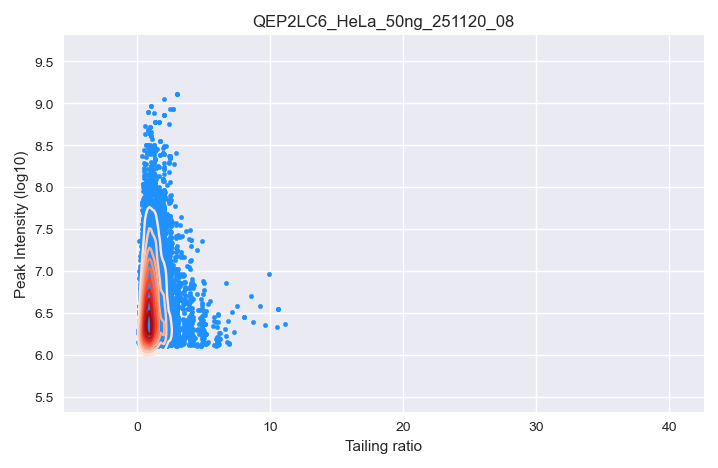

Supplement: Supplementary file 1 — pr0c00956_si_002.zip [file pr0c00956_si_002.zip › RawBeans_report/resources/images/QEP2LC6_HeLa_50ng_251120_08-peak-intentsity-vs-t2-t1-ratio.png]

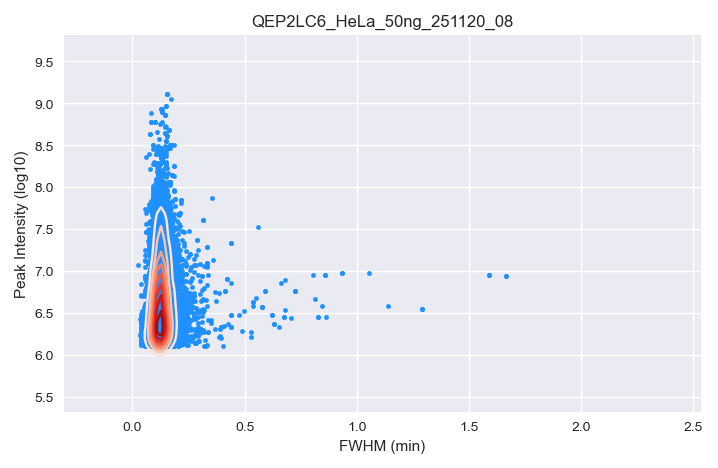

Supplement: Supplementary file 1 — pr0c00956_si_002.zip [file pr0c00956_si_002.zip › RawBeans_report/resources/images/QEP2LC6_HeLa_50ng_251120_08-peak-intentsity-vs-t-sum.png]

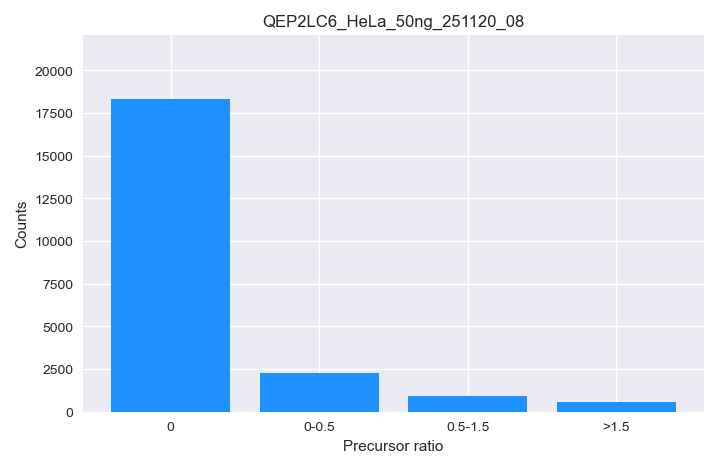

Supplement: Supplementary file 1 — pr0c00956_si_002.zip [file pr0c00956_si_002.zip › RawBeans_report/resources/images/QEP2LC6_HeLa_50ng_251120_08-prec-ratio.png]

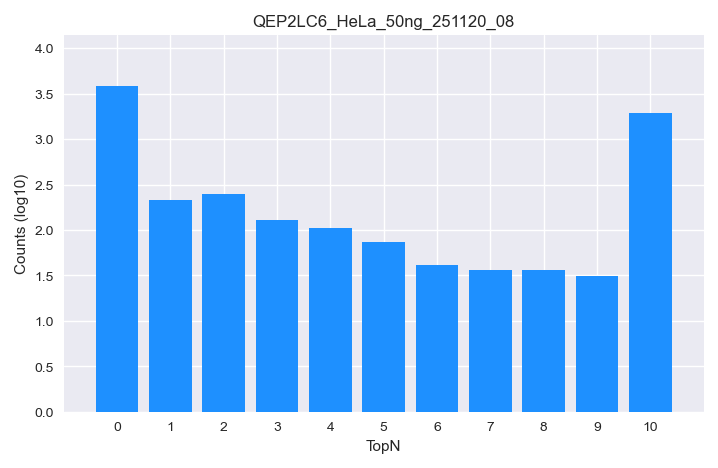

Supplement: Supplementary file 1 — pr0c00956_si_002.zip [file pr0c00956_si_002.zip › RawBeans_report/resources/images/QEP2LC6_HeLa_50ng_251120_08-top-n.png]

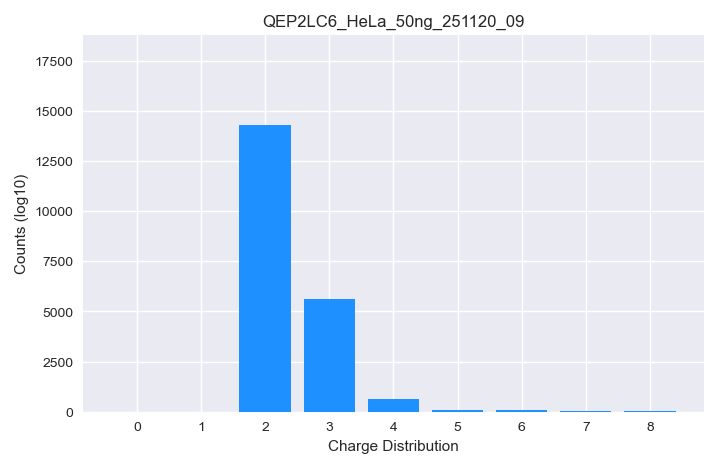

Supplement: Supplementary file 1 — pr0c00956_si_002.zip [file pr0c00956_si_002.zip › RawBeans_report/resources/images/QEP2LC6_HeLa_50ng_251120_09-charge-state.png]

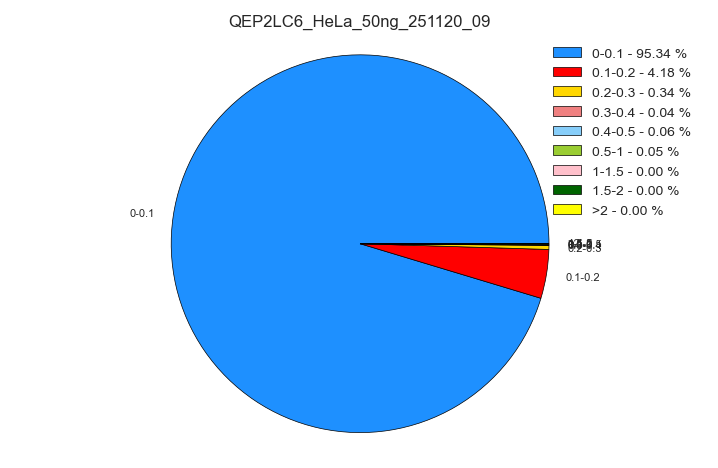

Supplement: Supplementary file 1 — pr0c00956_si_002.zip [file pr0c00956_si_002.zip › RawBeans_report/resources/images/QEP2LC6_HeLa_50ng_251120_09-fmhw-pie.png]

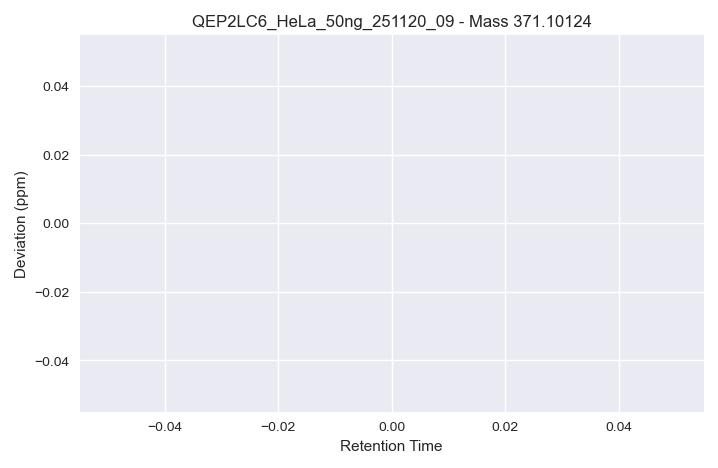

Supplement: Supplementary file 1 — pr0c00956_si_002.zip [file pr0c00956_si_002.zip › RawBeans_report/resources/images/QEP2LC6_HeLa_50ng_251120_09-mass-deviation1.png]

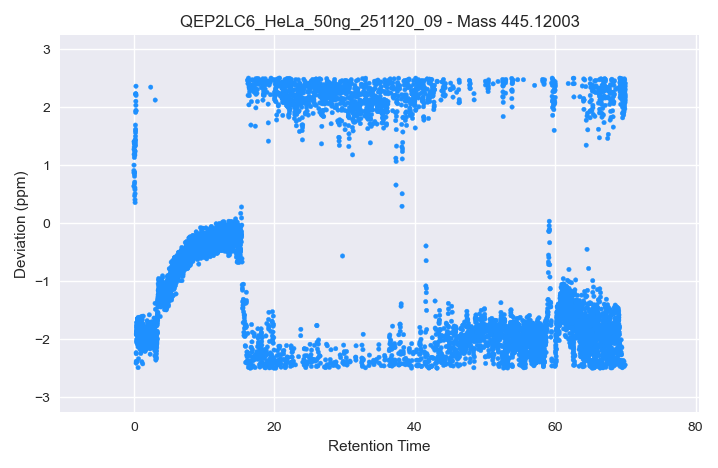

Supplement: Supplementary file 1 — pr0c00956_si_002.zip [file pr0c00956_si_002.zip › RawBeans_report/resources/images/QEP2LC6_HeLa_50ng_251120_09-mass-deviation2.png]

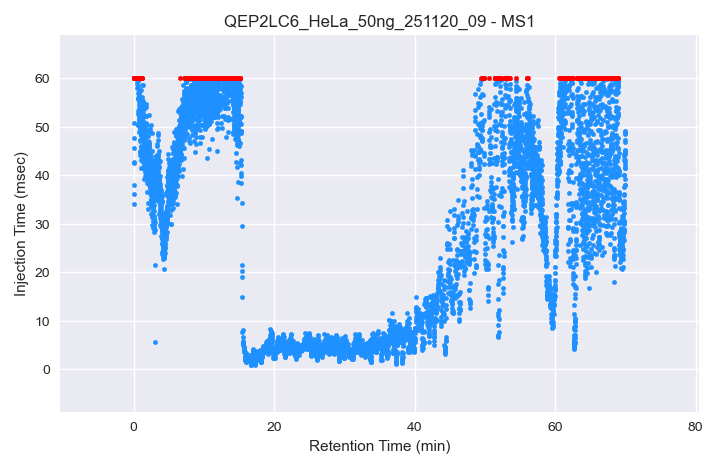

Supplement: Supplementary file 1 — pr0c00956_si_002.zip [file pr0c00956_si_002.zip › RawBeans_report/resources/images/QEP2LC6_HeLa_50ng_251120_09-ms1-inject-vs-ret.png]

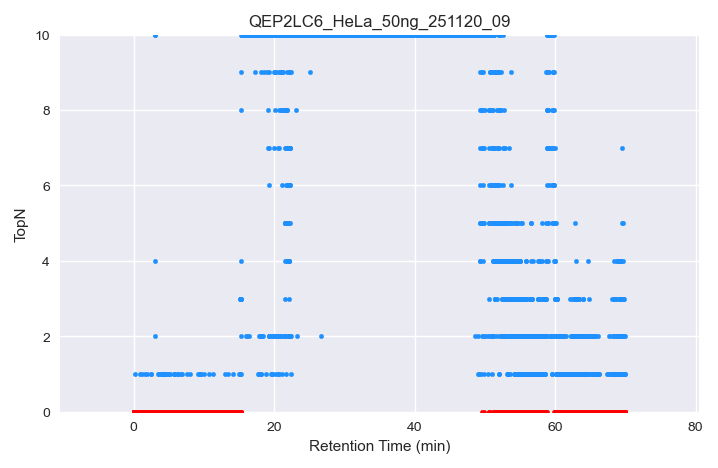

Supplement: Supplementary file 1 — pr0c00956_si_002.zip [file pr0c00956_si_002.zip › RawBeans_report/resources/images/QEP2LC6_HeLa_50ng_251120_09-ms1-ret-vs-top-n.png]

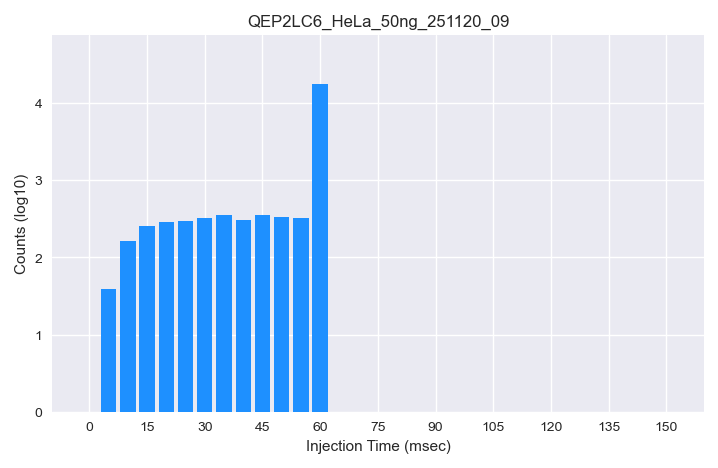

Supplement: Supplementary file 1 — pr0c00956_si_002.zip [file pr0c00956_si_002.zip › RawBeans_report/resources/images/QEP2LC6_HeLa_50ng_251120_09-ms2-inject.png]

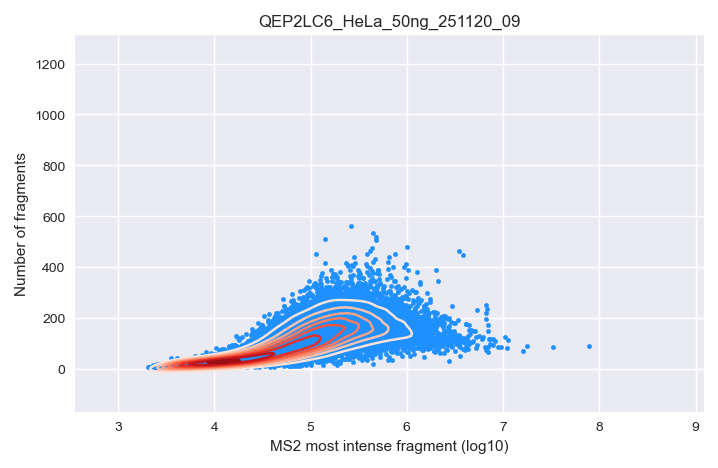

Supplement: Supplementary file 1 — pr0c00956_si_002.zip [file pr0c00956_si_002.zip › RawBeans_report/resources/images/QEP2LC6_HeLa_50ng_251120_09-ms2-max-log-intensity-vs-ms2-num-intensities.png]

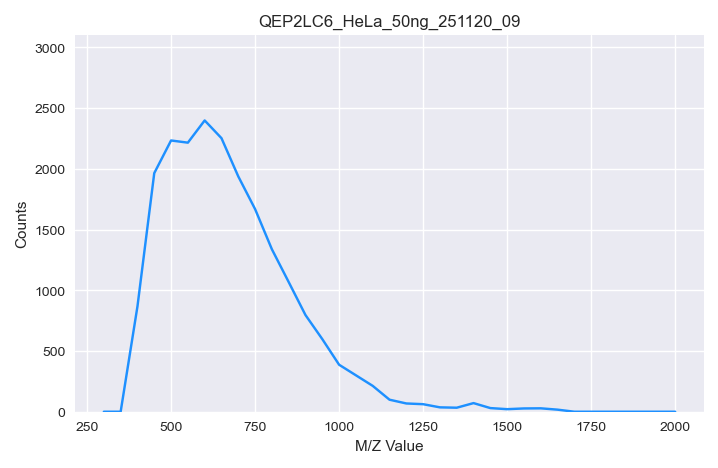

Supplement: Supplementary file 1 — pr0c00956_si_002.zip [file pr0c00956_si_002.zip › RawBeans_report/resources/images/QEP2LC6_HeLa_50ng_251120_09-ms2-mz-value.png]

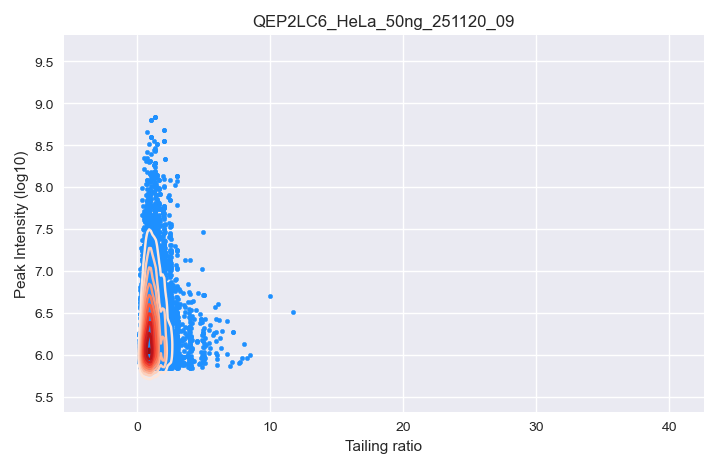

Supplement: Supplementary file 1 — pr0c00956_si_002.zip [file pr0c00956_si_002.zip › RawBeans_report/resources/images/QEP2LC6_HeLa_50ng_251120_09-peak-intentsity-vs-t2-t1-ratio.png]

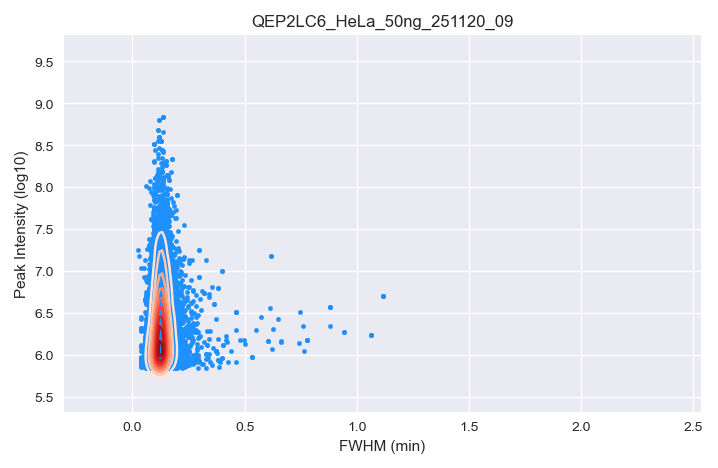

Supplement: Supplementary file 1 — pr0c00956_si_002.zip [file pr0c00956_si_002.zip › RawBeans_report/resources/images/QEP2LC6_HeLa_50ng_251120_09-peak-intentsity-vs-t-sum.png]

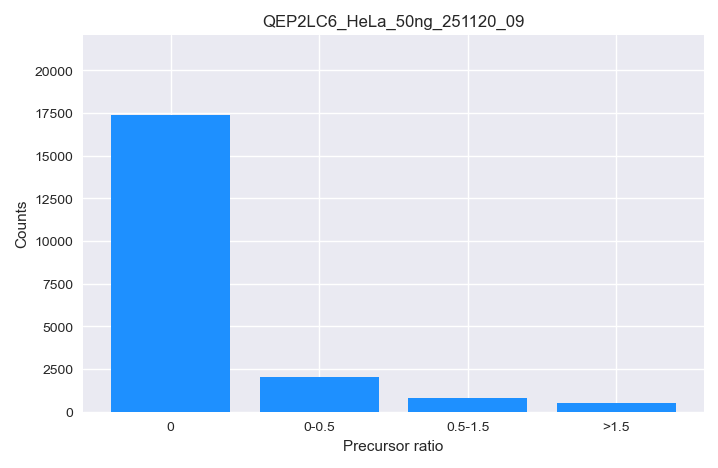

Supplement: Supplementary file 1 — pr0c00956_si_002.zip [file pr0c00956_si_002.zip › RawBeans_report/resources/images/QEP2LC6_HeLa_50ng_251120_09-prec-ratio.png]

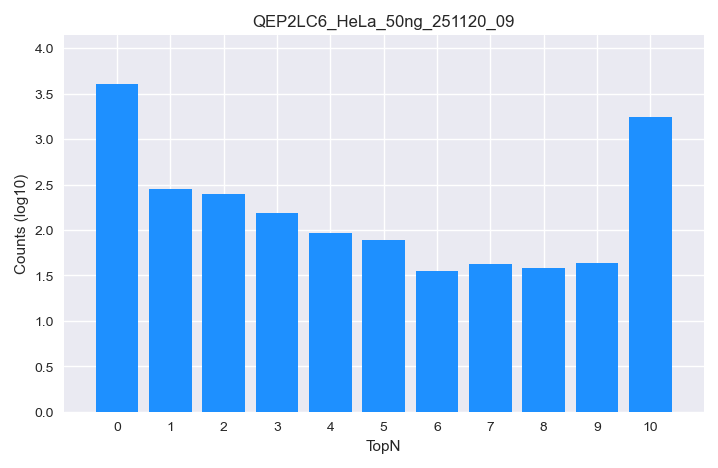

Supplement: Supplementary file 1 — pr0c00956_si_002.zip [file pr0c00956_si_002.zip › RawBeans_report/resources/images/QEP2LC6_HeLa_50ng_251120_09-top-n.png]

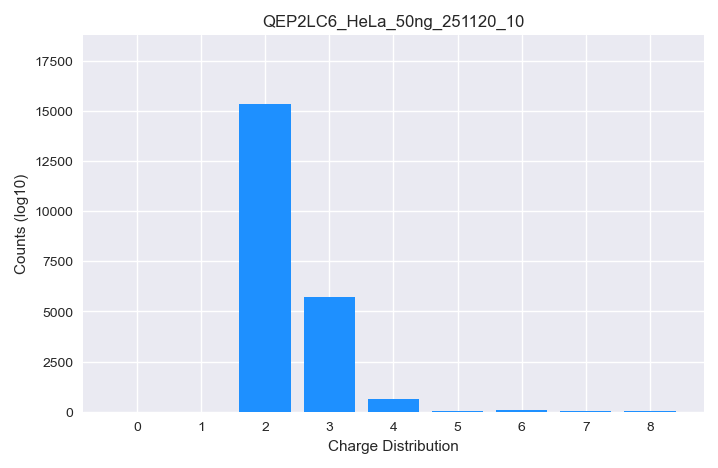

Supplement: Supplementary file 1 — pr0c00956_si_002.zip [file pr0c00956_si_002.zip › RawBeans_report/resources/images/QEP2LC6_HeLa_50ng_251120_10-charge-state.png]

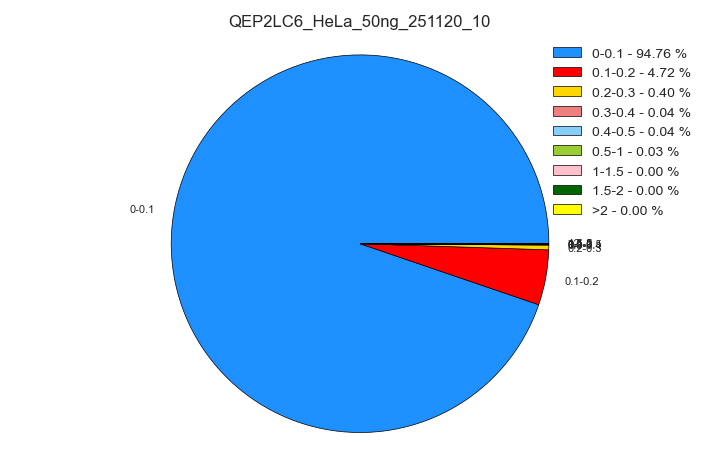

Supplement: Supplementary file 1 — pr0c00956_si_002.zip [file pr0c00956_si_002.zip › RawBeans_report/resources/images/QEP2LC6_HeLa_50ng_251120_10-fmhw-pie.png]

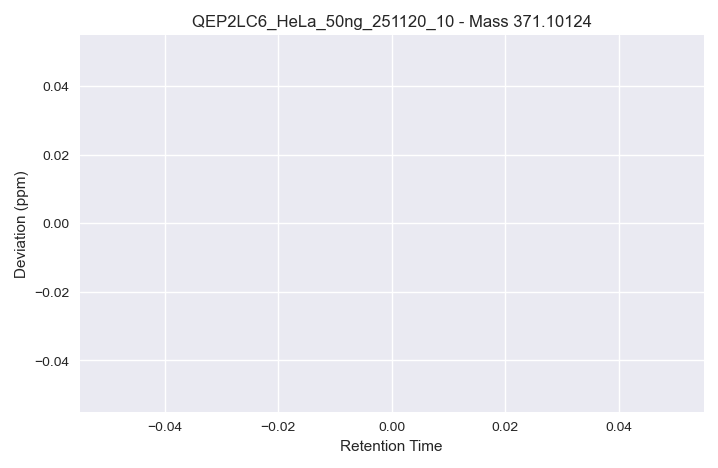

Supplement: Supplementary file 1 — pr0c00956_si_002.zip [file pr0c00956_si_002.zip › RawBeans_report/resources/images/QEP2LC6_HeLa_50ng_251120_10-mass-deviation1.png]

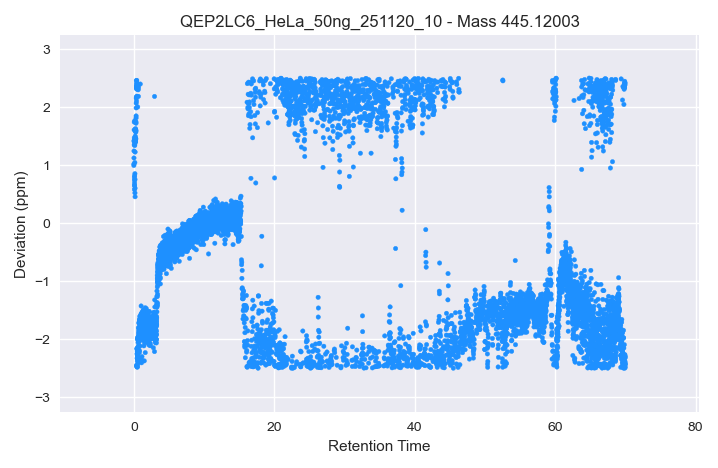

Supplement: Supplementary file 1 — pr0c00956_si_002.zip [file pr0c00956_si_002.zip › RawBeans_report/resources/images/QEP2LC6_HeLa_50ng_251120_10-mass-deviation2.png]

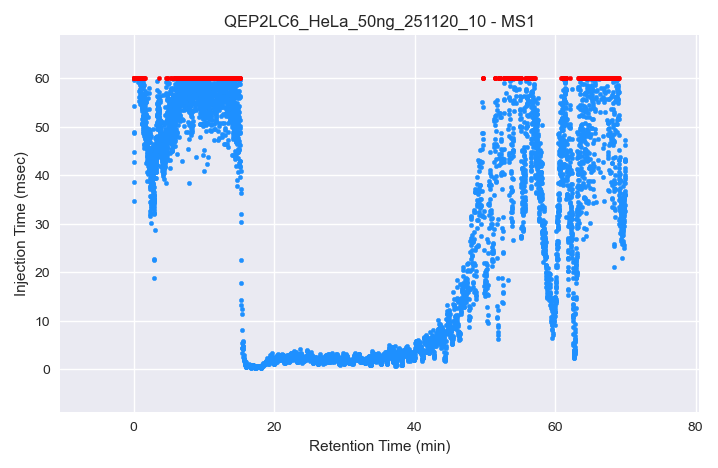

Supplement: Supplementary file 1 — pr0c00956_si_002.zip [file pr0c00956_si_002.zip › RawBeans_report/resources/images/QEP2LC6_HeLa_50ng_251120_10-ms1-inject-vs-ret.png]

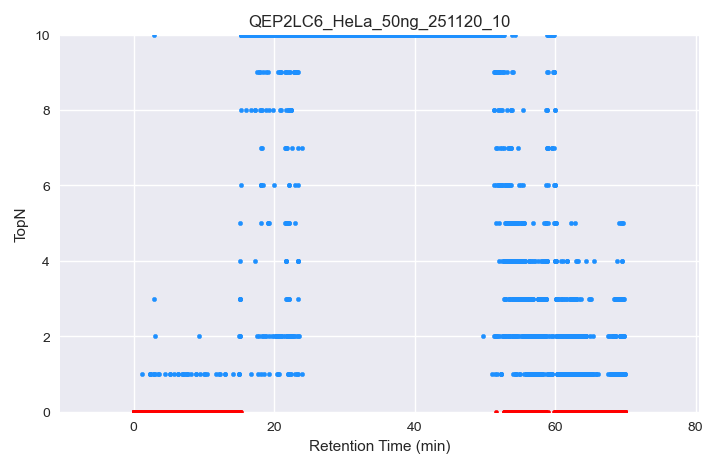

Supplement: Supplementary file 1 — pr0c00956_si_002.zip [file pr0c00956_si_002.zip › RawBeans_report/resources/images/QEP2LC6_HeLa_50ng_251120_10-ms1-ret-vs-top-n.png]

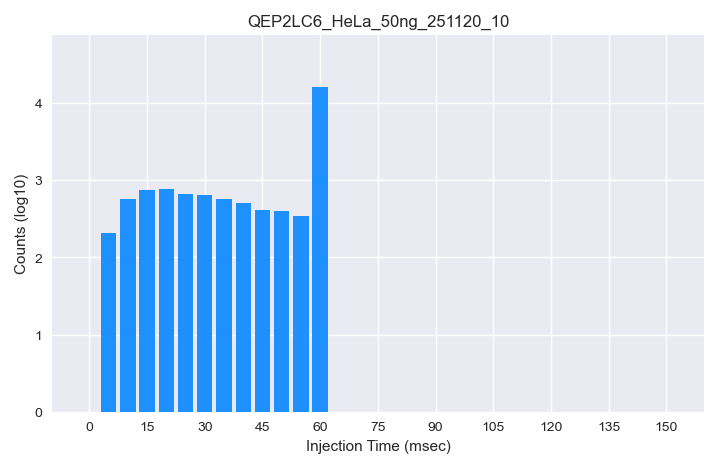

Supplement: Supplementary file 1 — pr0c00956_si_002.zip [file pr0c00956_si_002.zip › RawBeans_report/resources/images/QEP2LC6_HeLa_50ng_251120_10-ms2-inject.png]

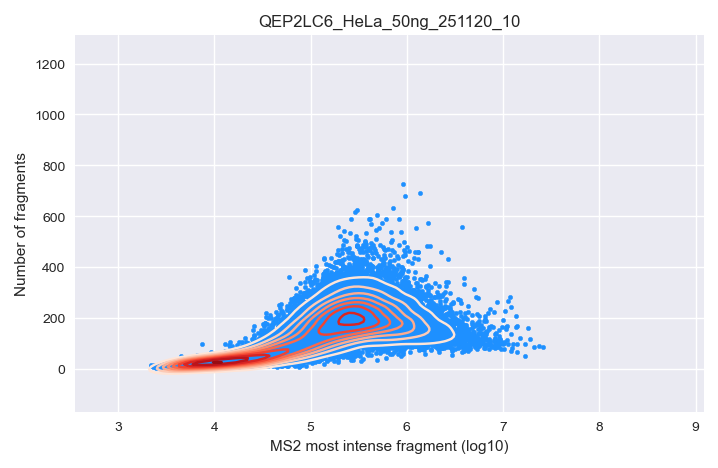

Supplement: Supplementary file 1 — pr0c00956_si_002.zip [file pr0c00956_si_002.zip › RawBeans_report/resources/images/QEP2LC6_HeLa_50ng_251120_10-ms2-max-log-intensity-vs-ms2-num-intensities.png]

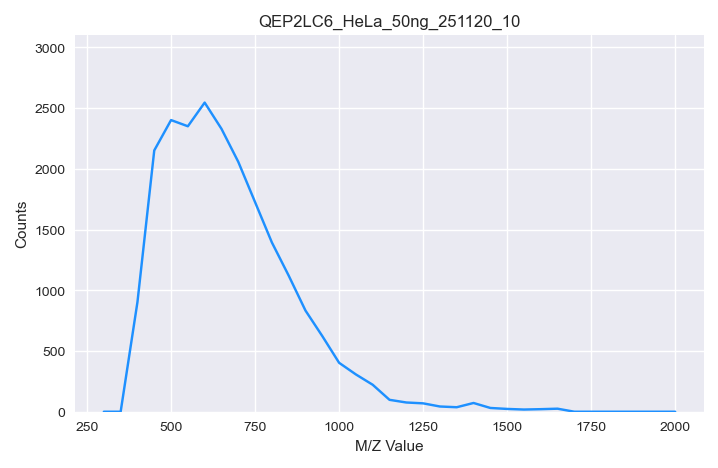

Supplement: Supplementary file 1 — pr0c00956_si_002.zip [file pr0c00956_si_002.zip › RawBeans_report/resources/images/QEP2LC6_HeLa_50ng_251120_10-ms2-mz-value.png]

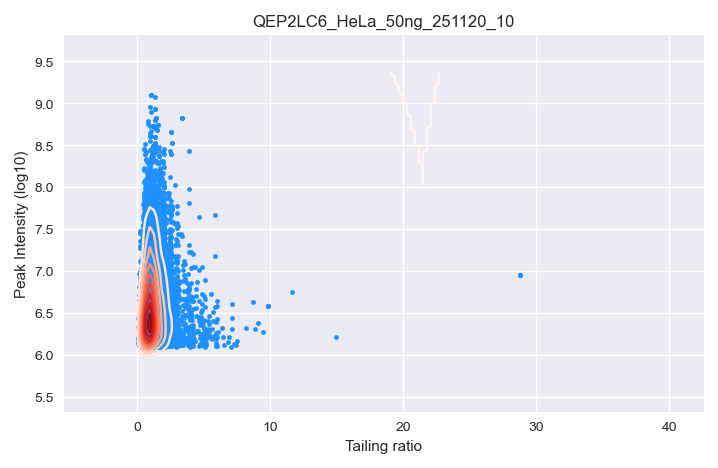

Supplement: Supplementary file 1 — pr0c00956_si_002.zip [file pr0c00956_si_002.zip › RawBeans_report/resources/images/QEP2LC6_HeLa_50ng_251120_10-peak-intentsity-vs-t2-t1-ratio.png]

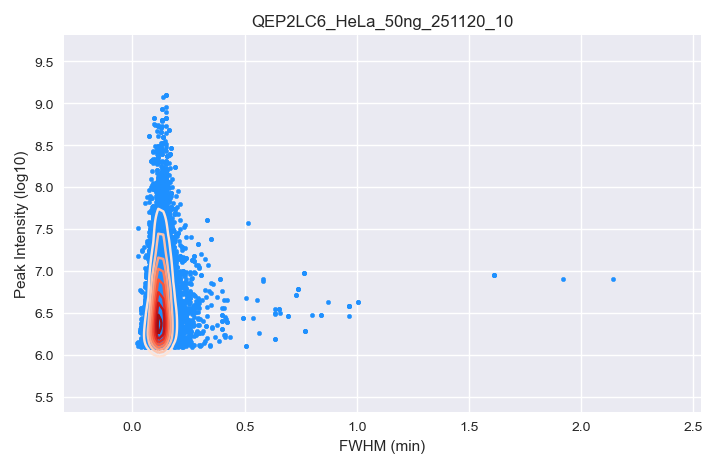

Supplement: Supplementary file 1 — pr0c00956_si_002.zip [file pr0c00956_si_002.zip › RawBeans_report/resources/images/QEP2LC6_HeLa_50ng_251120_10-peak-intentsity-vs-t-sum.png]

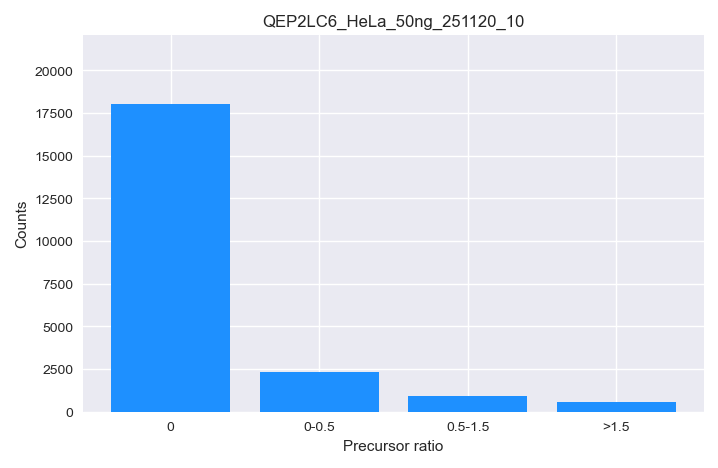

Supplement: Supplementary file 1 — pr0c00956_si_002.zip [file pr0c00956_si_002.zip › RawBeans_report/resources/images/QEP2LC6_HeLa_50ng_251120_10-prec-ratio.png]

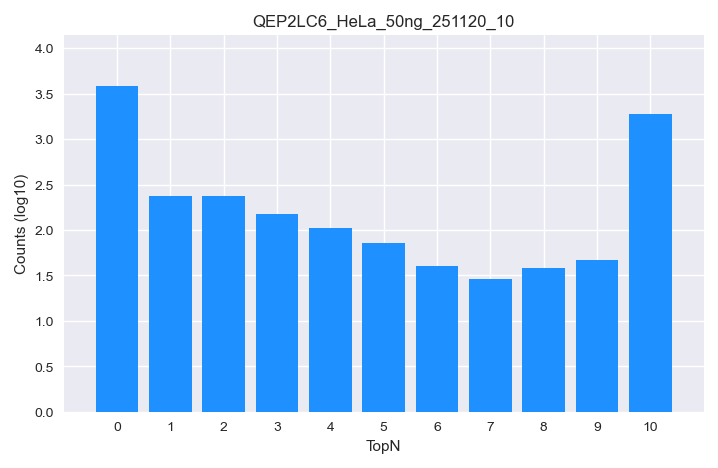

Supplement: Supplementary file 1 — pr0c00956_si_002.zip [file pr0c00956_si_002.zip › RawBeans_report/resources/images/QEP2LC6_HeLa_50ng_251120_10-top-n.png]

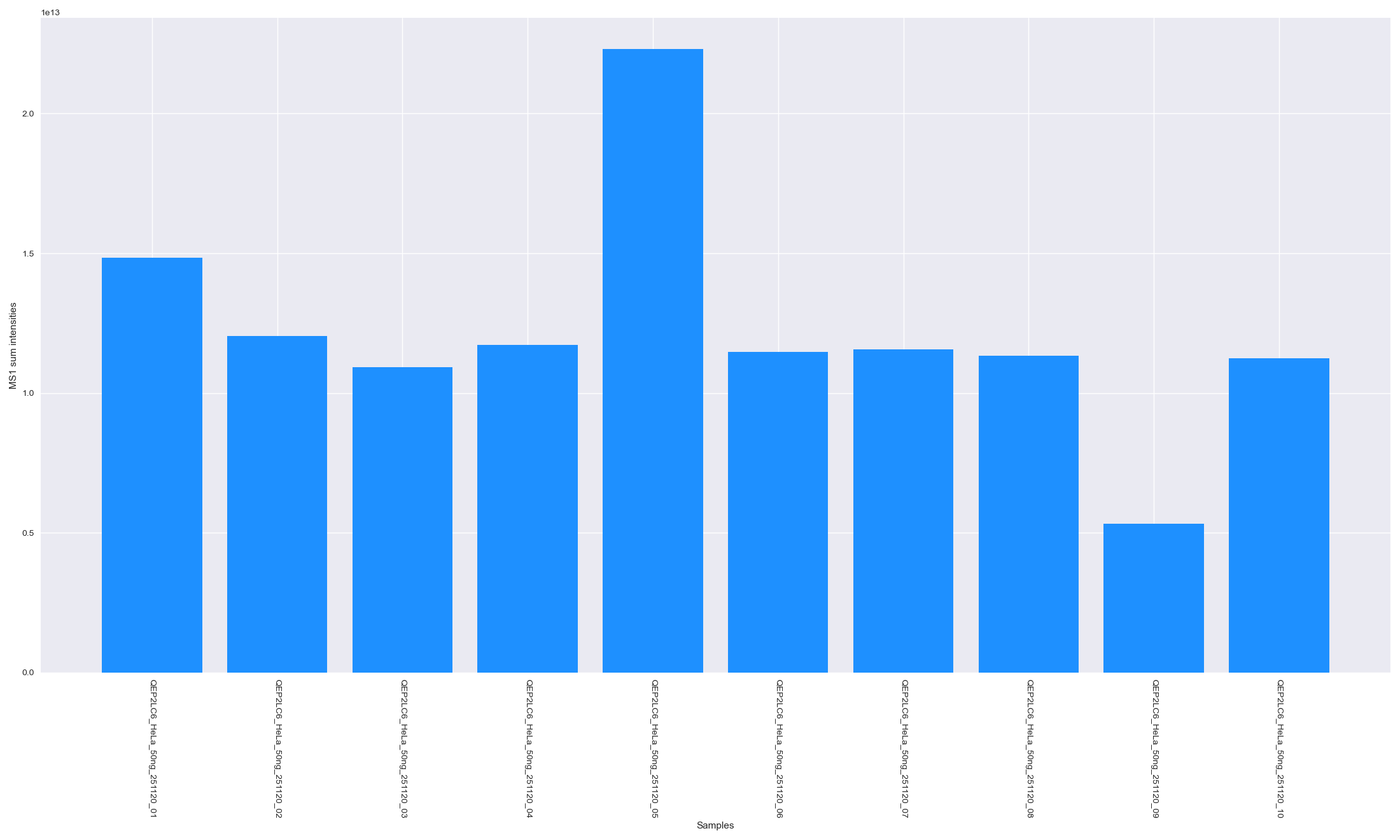

Supplement: Supplementary file 1 — pr0c00956_si_002.zip [file pr0c00956_si_002.zip › RawBeans_report/resources/images/tic-lex-sort.png]

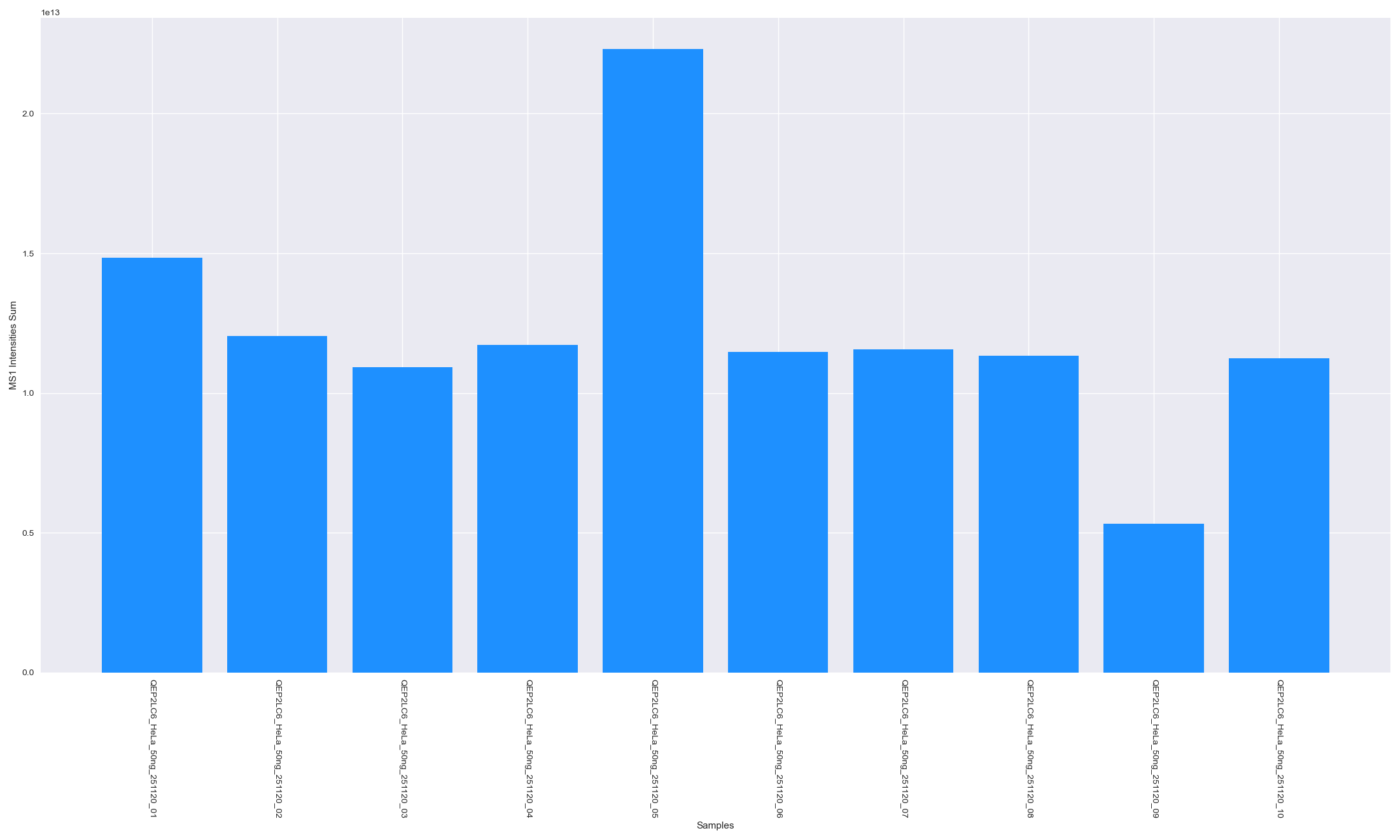

Supplement: Supplementary file 1 — pr0c00956_si_002.zip [file pr0c00956_si_002.zip › RawBeans_report/resources/images/tic-run-date-sort.png]

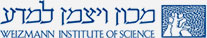

Supplement: Supplementary file 1 — pr0c00956_si_002.zip [file pr0c00956_si_002.zip › RawBeans_report/resources/images/weizmann_logo.jpg]

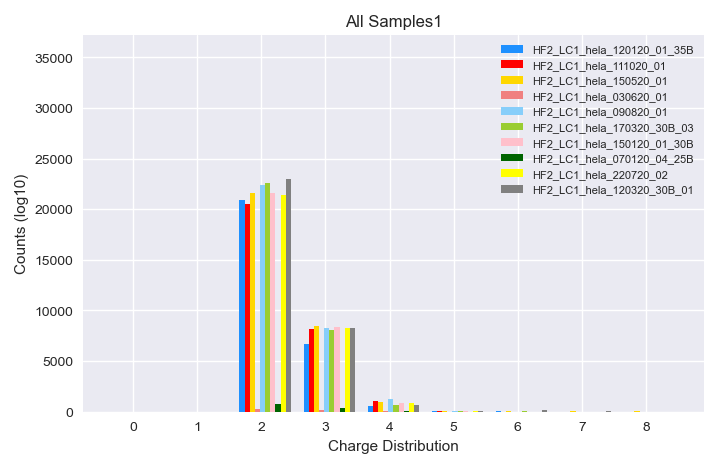

Supplement: Supplementary file 2 — pr0c00956_si_003.zip [file pr0c00956_si_003.zip › 100_samples_run1.0/resources/images/all-samples1-charge-state.png]

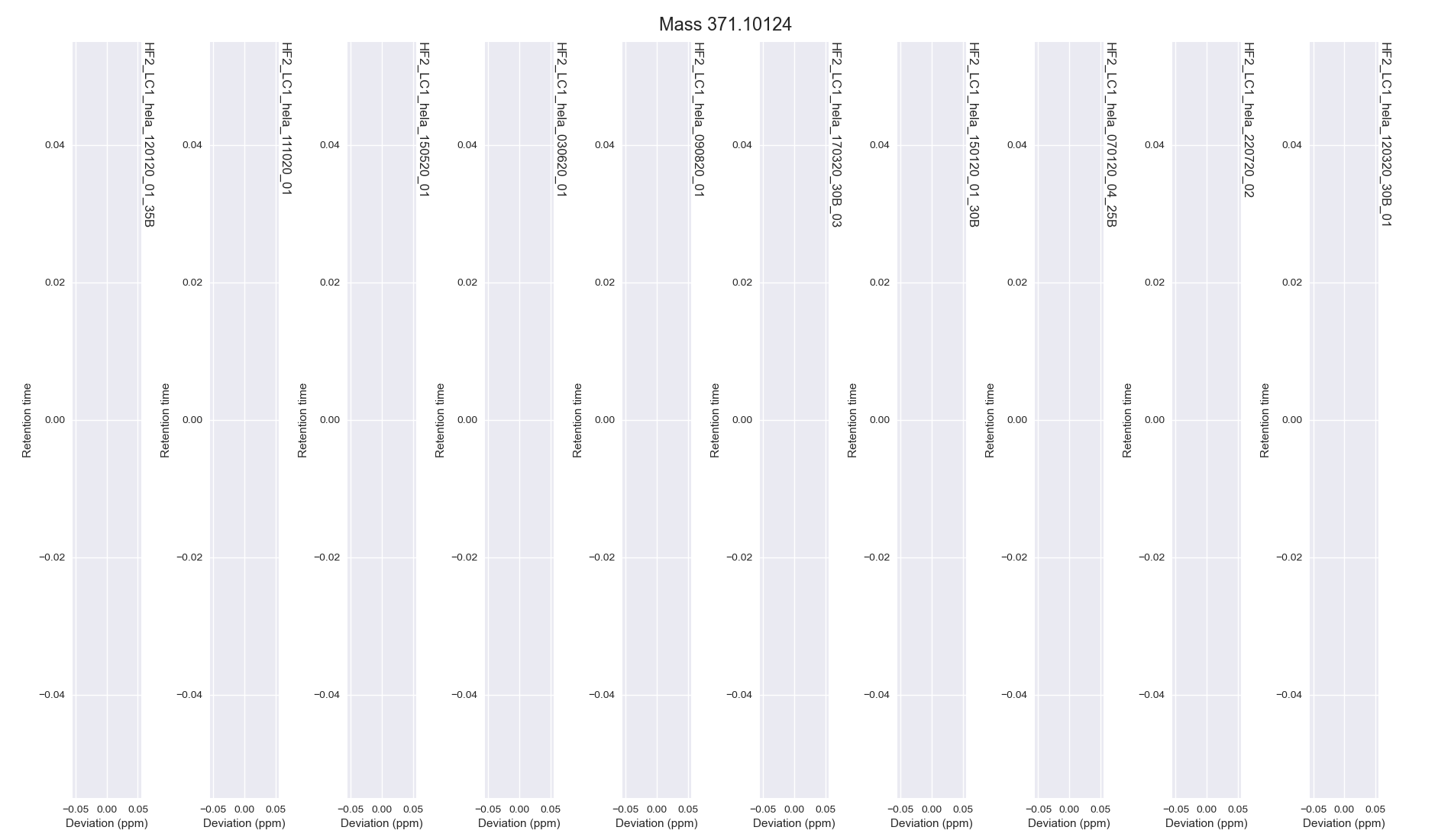

Supplement: Supplementary file 2 — pr0c00956_si_003.zip [file pr0c00956_si_003.zip › 100_samples_run1.0/resources/images/all-samples1-mass1-deviation.png]

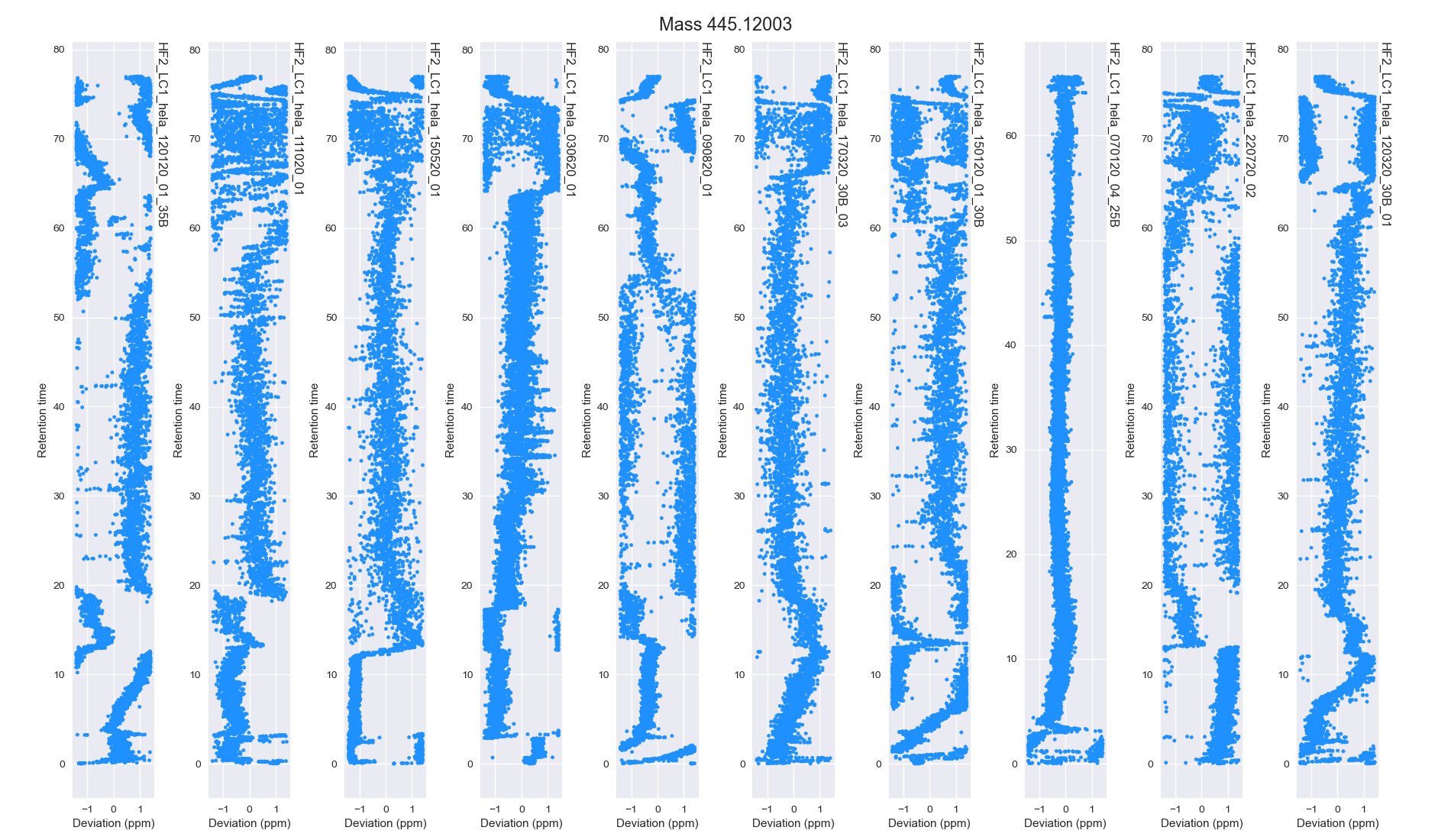

Supplement: Supplementary file 2 — pr0c00956_si_003.zip [file pr0c00956_si_003.zip › 100_samples_run1.0/resources/images/all-samples1-mass2-deviation.png]

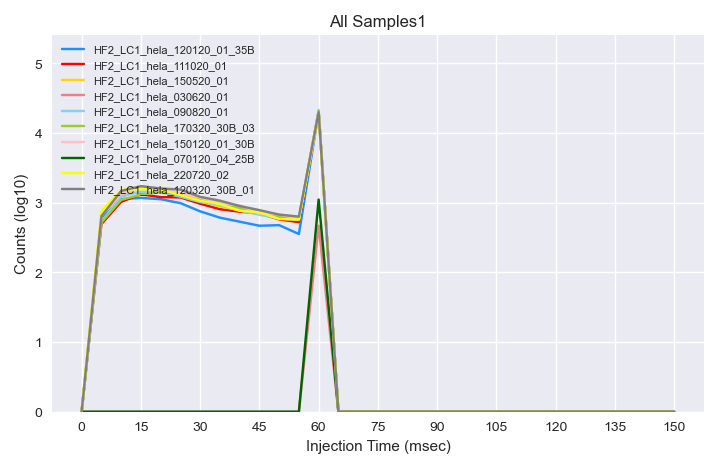

Supplement: Supplementary file 2 — pr0c00956_si_003.zip [file pr0c00956_si_003.zip › 100_samples_run1.0/resources/images/all-samples1-ms2-inject.png]

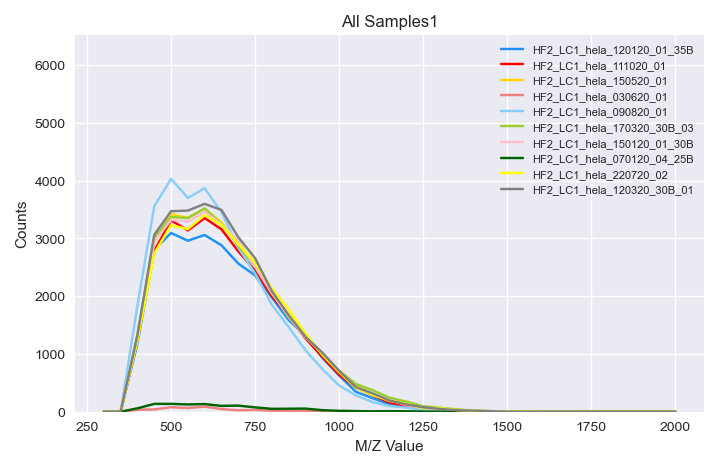

Supplement: Supplementary file 2 — pr0c00956_si_003.zip [file pr0c00956_si_003.zip › 100_samples_run1.0/resources/images/all-samples1-ms2-mz-value.png]

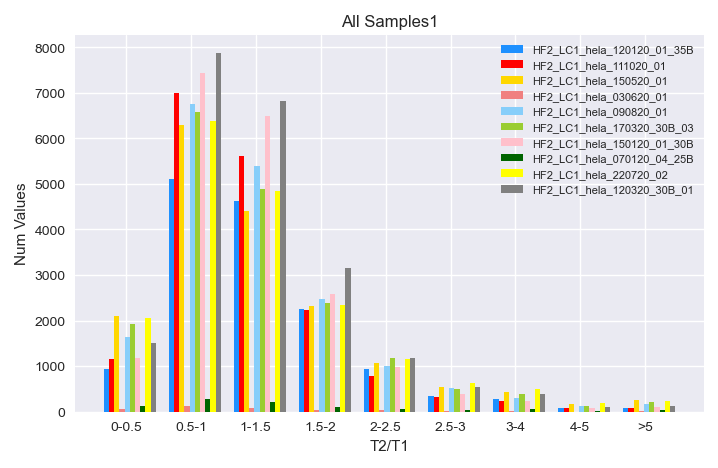

Supplement: Supplementary file 2 — pr0c00956_si_003.zip [file pr0c00956_si_003.zip › 100_samples_run1.0/resources/images/all-samples1-peak-symmetry.png]

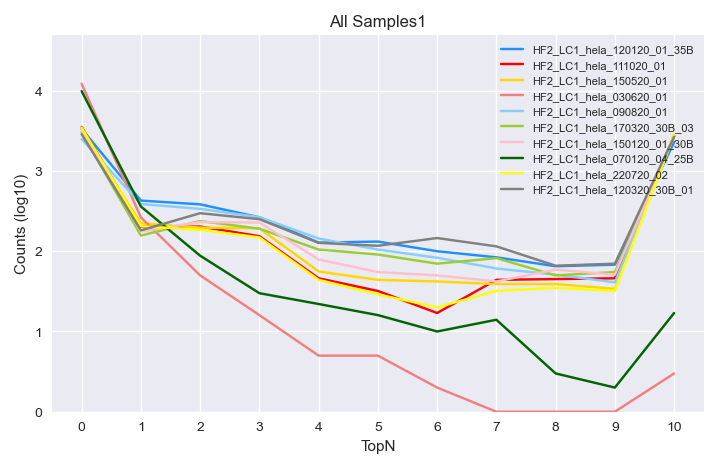

Supplement: Supplementary file 2 — pr0c00956_si_003.zip [file pr0c00956_si_003.zip › 100_samples_run1.0/resources/images/all-samples1-top-n.png]

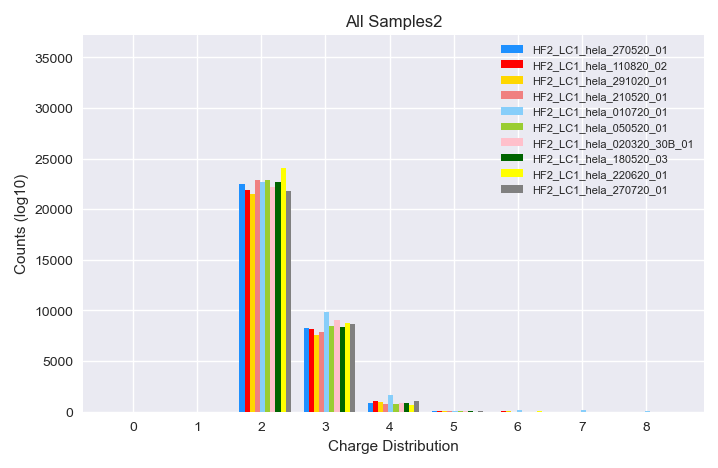

Supplement: Supplementary file 2 — pr0c00956_si_003.zip [file pr0c00956_si_003.zip › 100_samples_run1.0/resources/images/all-samples2-charge-state.png]

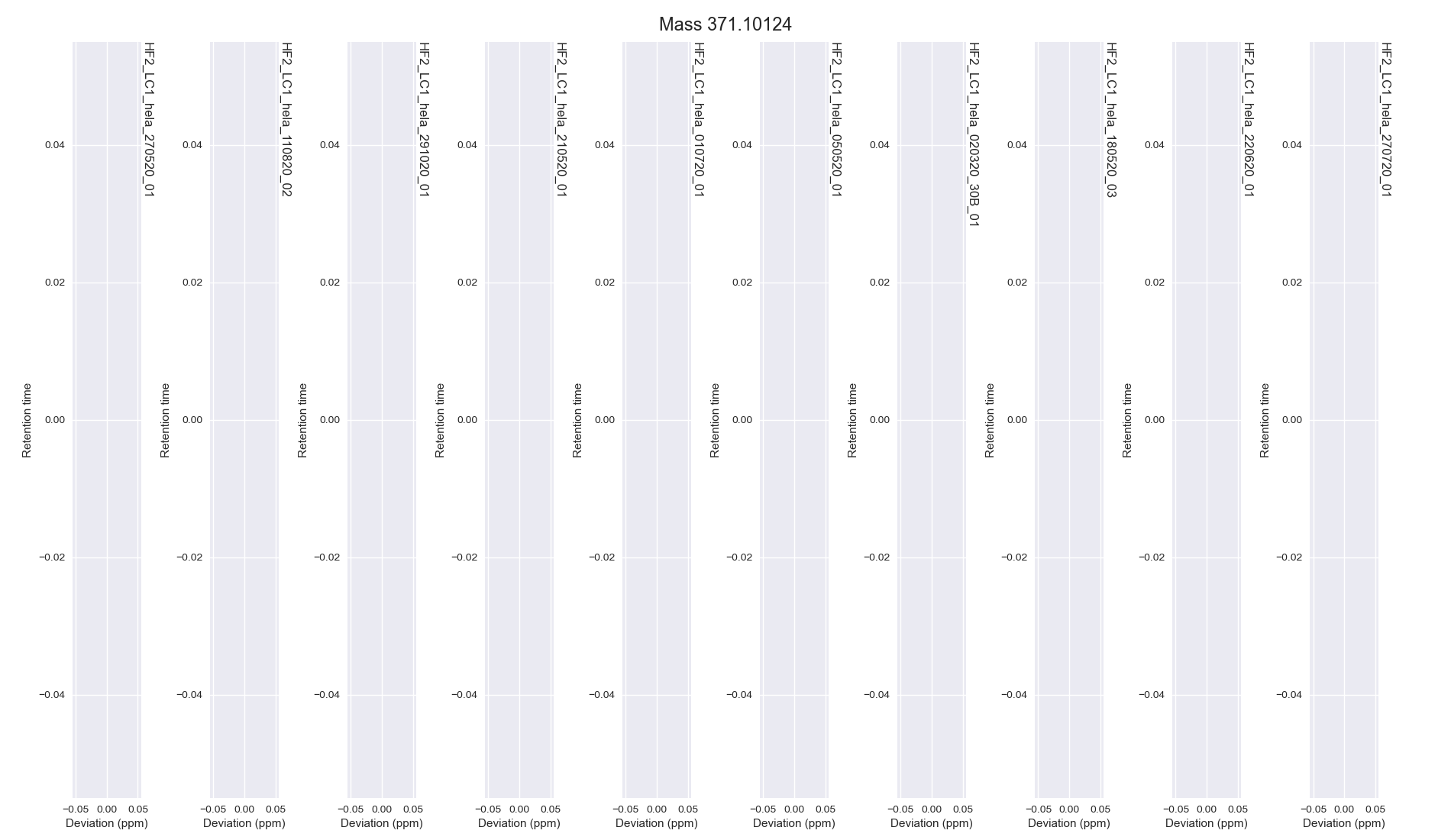

Supplement: Supplementary file 2 — pr0c00956_si_003.zip [file pr0c00956_si_003.zip › 100_samples_run1.0/resources/images/all-samples2-mass1-deviation.png]

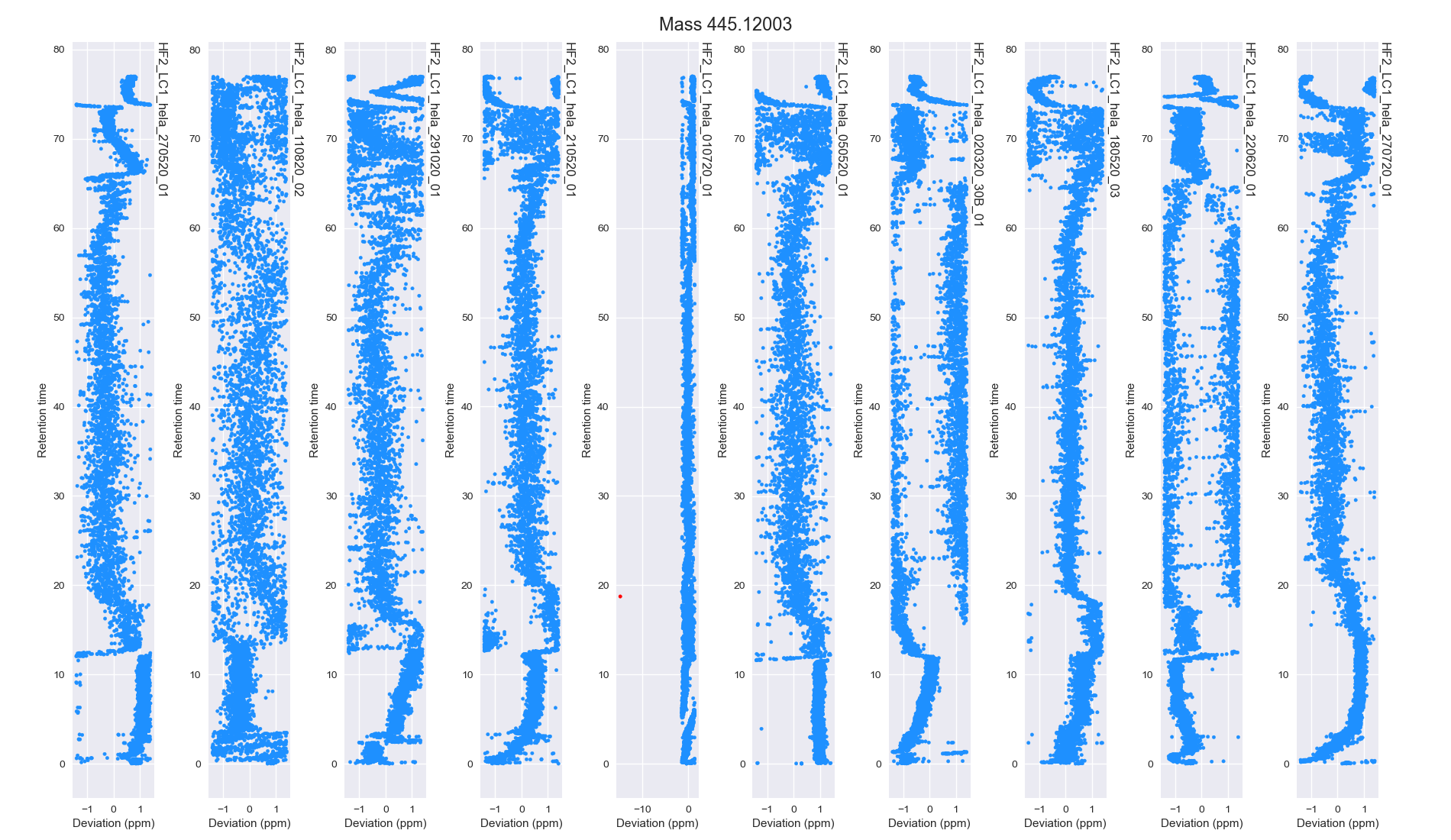

Supplement: Supplementary file 2 — pr0c00956_si_003.zip [file pr0c00956_si_003.zip › 100_samples_run1.0/resources/images/all-samples2-mass2-deviation.png]

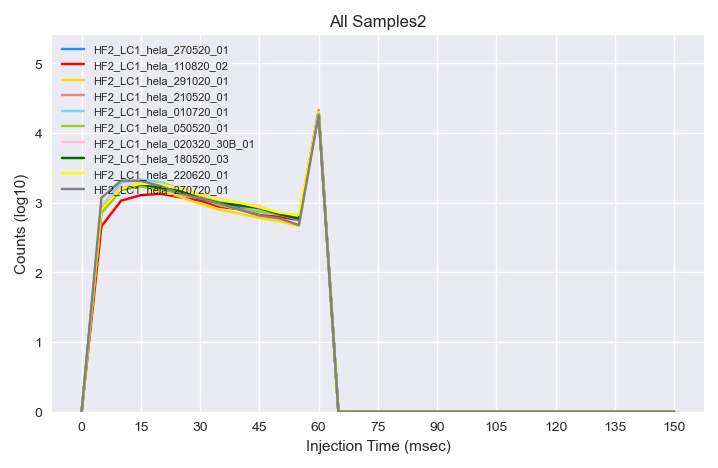

Supplement: Supplementary file 2 — pr0c00956_si_003.zip [file pr0c00956_si_003.zip › 100_samples_run1.0/resources/images/all-samples2-ms2-inject.png]

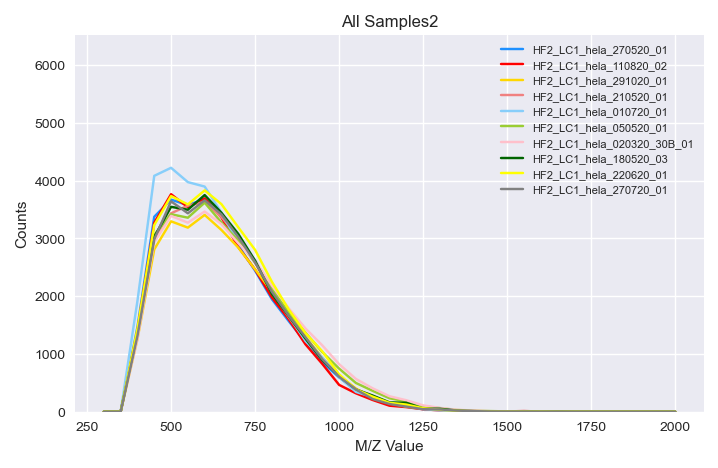

Supplement: Supplementary file 2 — pr0c00956_si_003.zip [file pr0c00956_si_003.zip › 100_samples_run1.0/resources/images/all-samples2-ms2-mz-value.png]

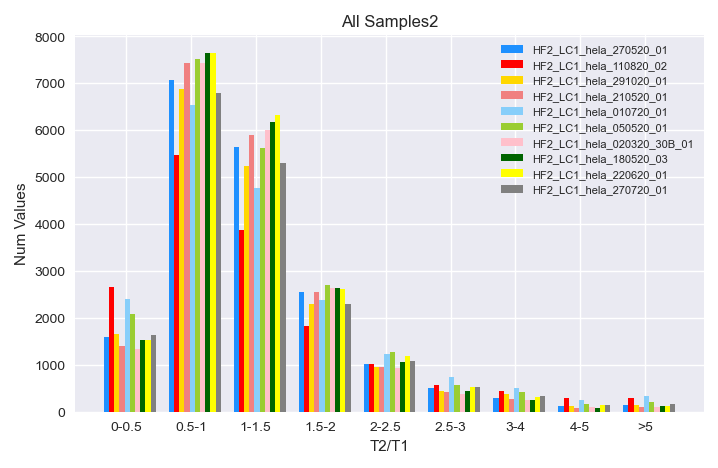

Supplement: Supplementary file 2 — pr0c00956_si_003.zip [file pr0c00956_si_003.zip › 100_samples_run1.0/resources/images/all-samples2-peak-symmetry.png]

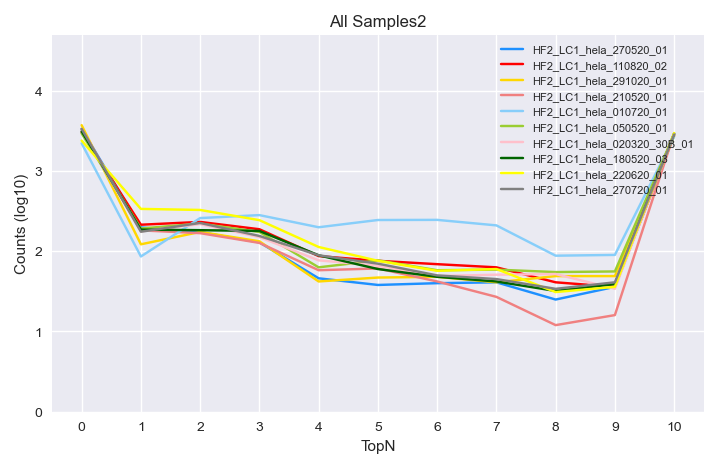

Supplement: Supplementary file 2 — pr0c00956_si_003.zip [file pr0c00956_si_003.zip › 100_samples_run1.0/resources/images/all-samples2-top-n.png]

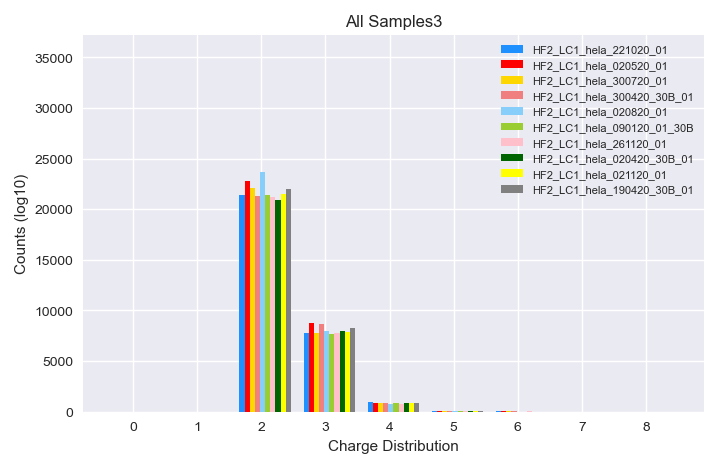

Supplement: Supplementary file 2 — pr0c00956_si_003.zip [file pr0c00956_si_003.zip › 100_samples_run1.0/resources/images/all-samples3-charge-state.png]

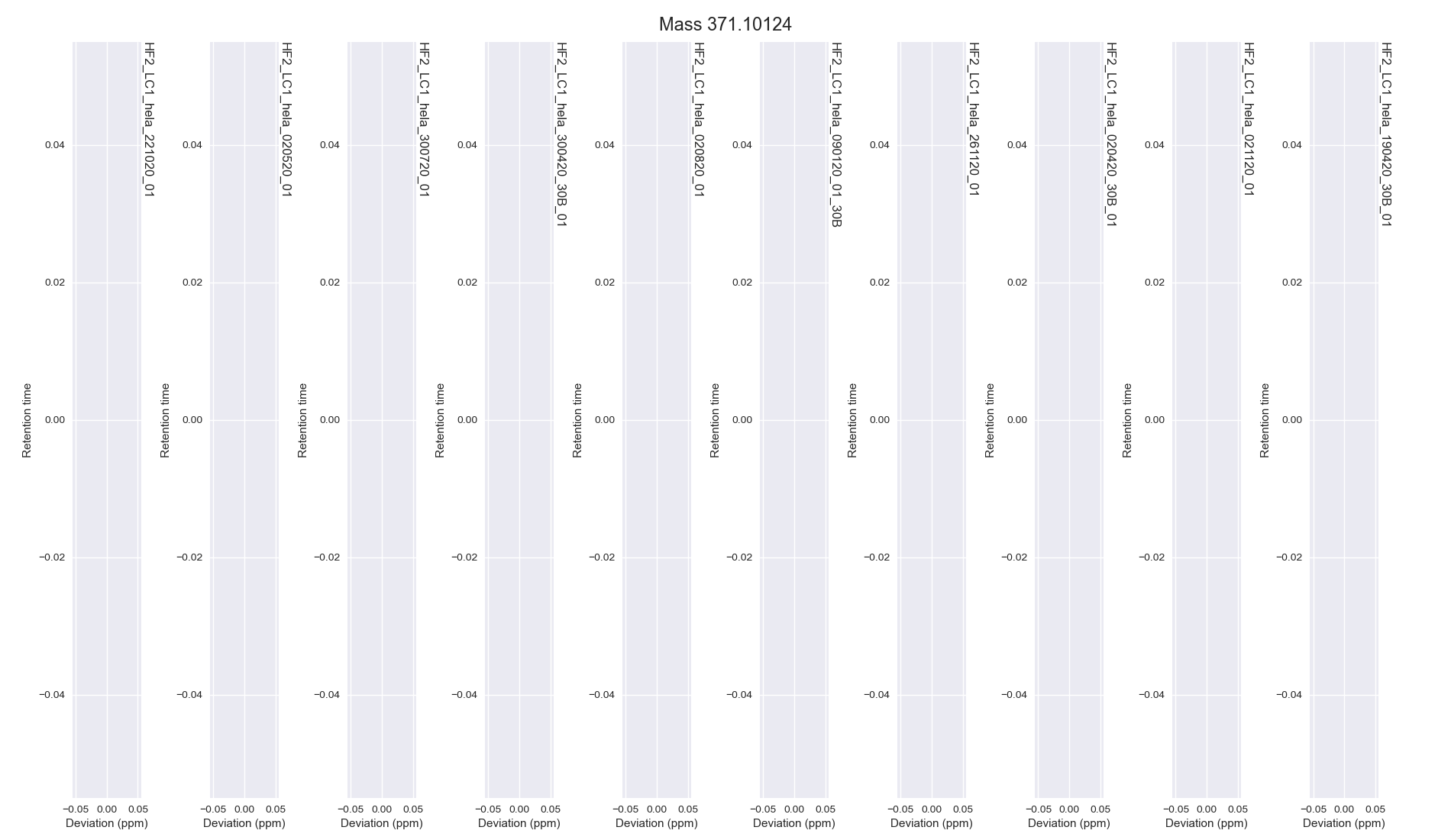

Supplement: Supplementary file 2 — pr0c00956_si_003.zip [file pr0c00956_si_003.zip › 100_samples_run1.0/resources/images/all-samples3-mass1-deviation.png]

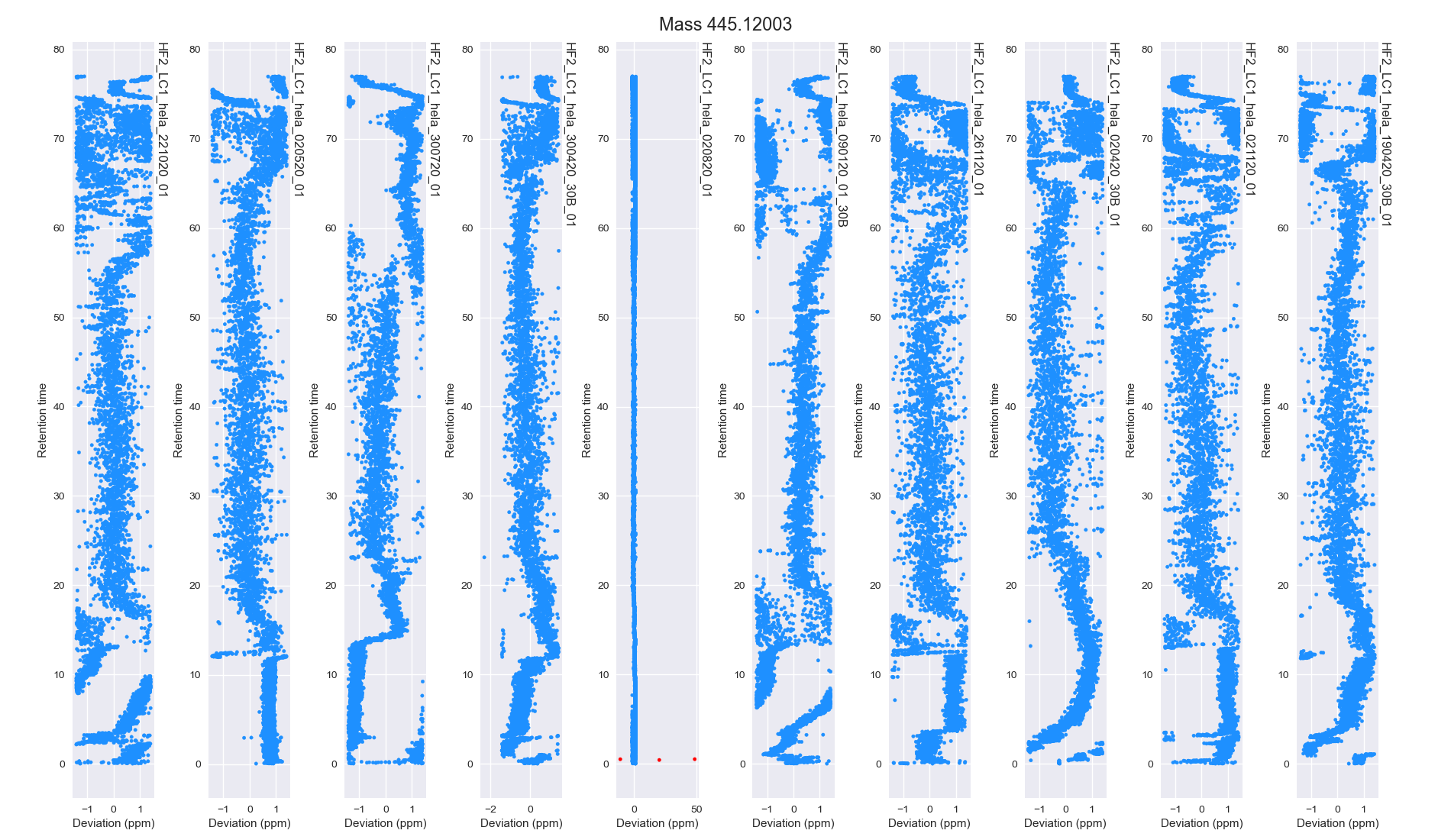

Supplement: Supplementary file 2 — pr0c00956_si_003.zip [file pr0c00956_si_003.zip › 100_samples_run1.0/resources/images/all-samples3-mass2-deviation.png]

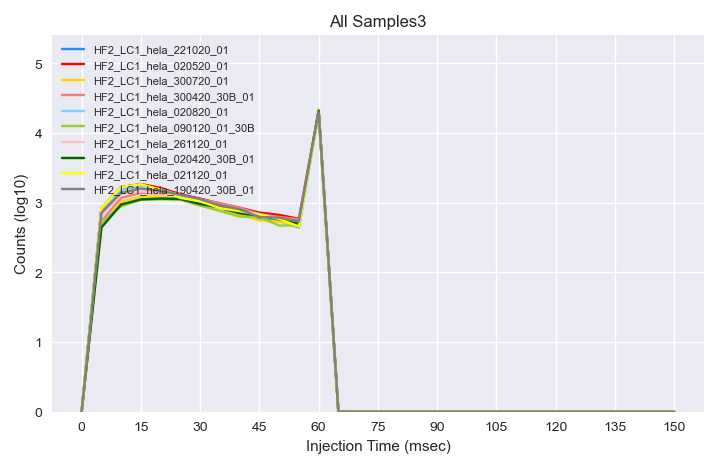

Supplement: Supplementary file 2 — pr0c00956_si_003.zip [file pr0c00956_si_003.zip › 100_samples_run1.0/resources/images/all-samples3-ms2-inject.png]

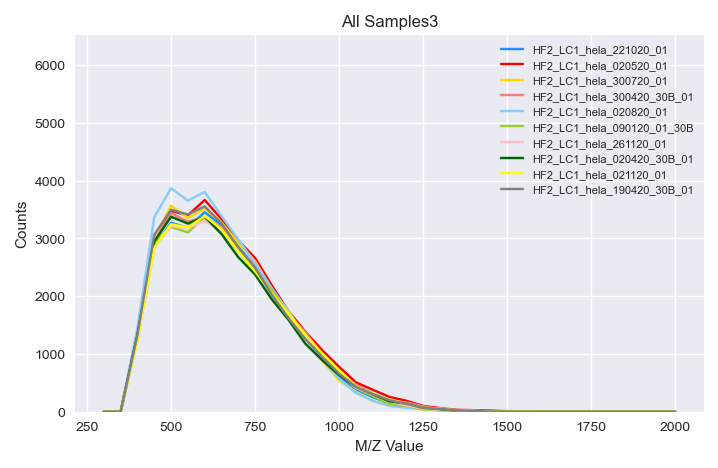

Supplement: Supplementary file 2 — pr0c00956_si_003.zip [file pr0c00956_si_003.zip › 100_samples_run1.0/resources/images/all-samples3-ms2-mz-value.png]

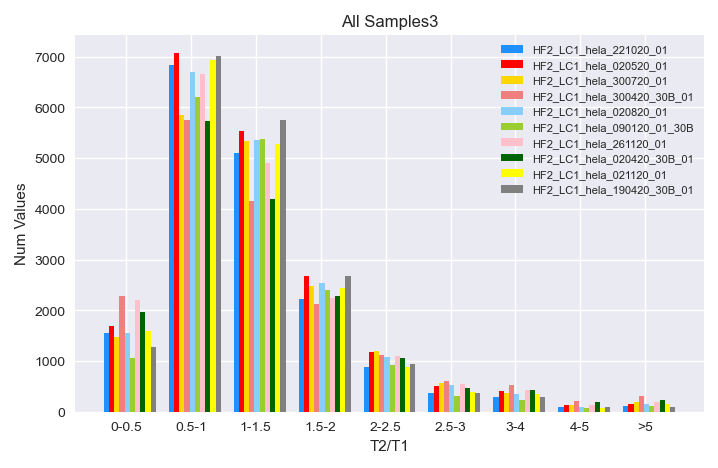

Supplement: Supplementary file 2 — pr0c00956_si_003.zip [file pr0c00956_si_003.zip › 100_samples_run1.0/resources/images/all-samples3-peak-symmetry.png]

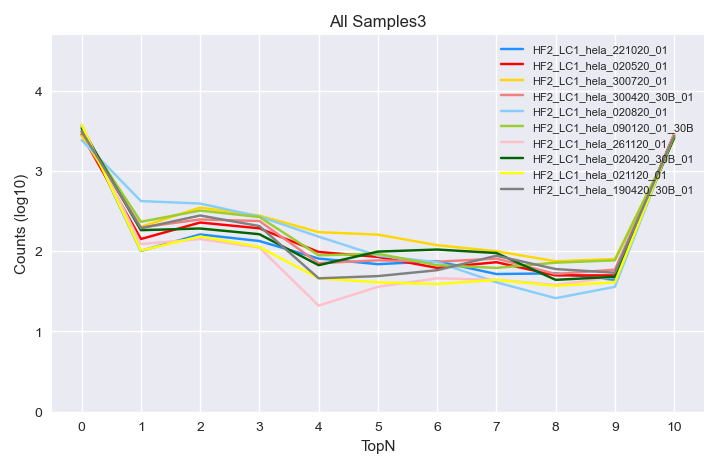

Supplement: Supplementary file 2 — pr0c00956_si_003.zip [file pr0c00956_si_003.zip › 100_samples_run1.0/resources/images/all-samples3-top-n.png]

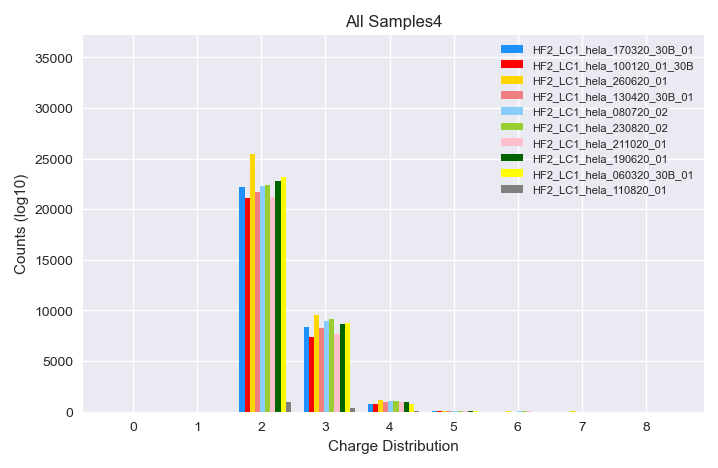

Supplement: Supplementary file 2 — pr0c00956_si_003.zip [file pr0c00956_si_003.zip › 100_samples_run1.0/resources/images/all-samples4-charge-state.png]

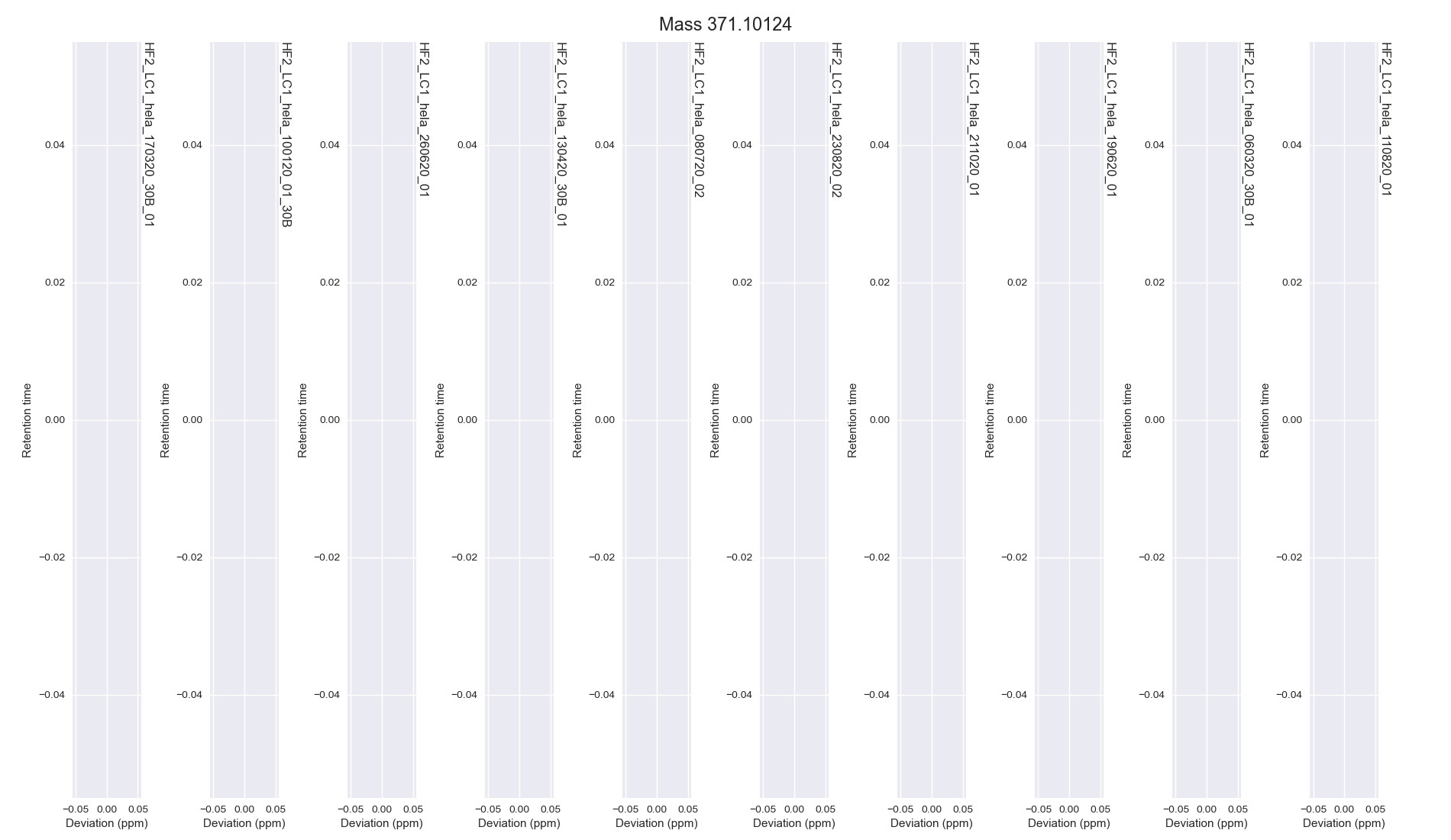

Supplement: Supplementary file 2 — pr0c00956_si_003.zip [file pr0c00956_si_003.zip › 100_samples_run1.0/resources/images/all-samples4-mass1-deviation.png]

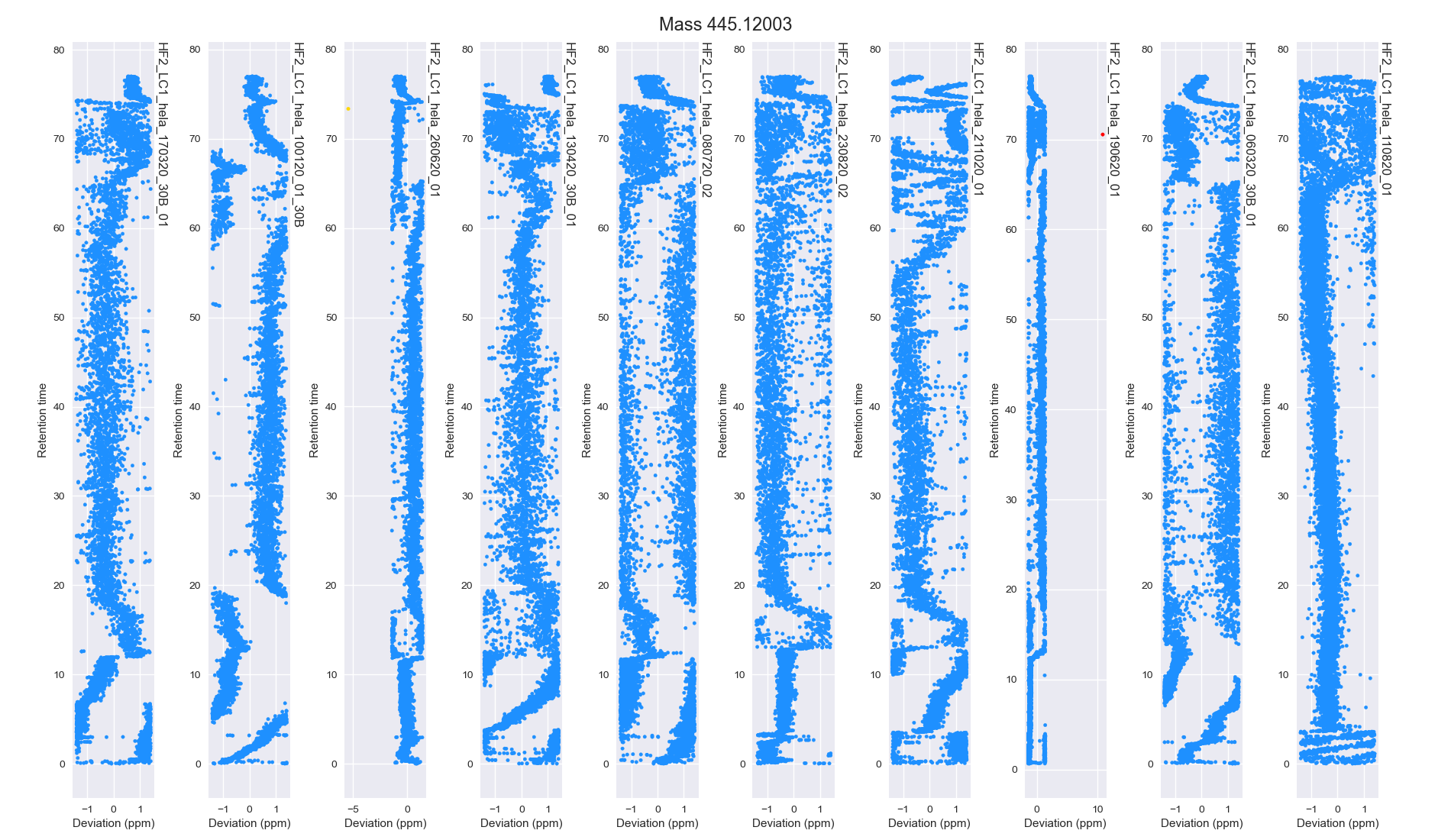

Supplement: Supplementary file 2 — pr0c00956_si_003.zip [file pr0c00956_si_003.zip › 100_samples_run1.0/resources/images/all-samples4-mass2-deviation.png]

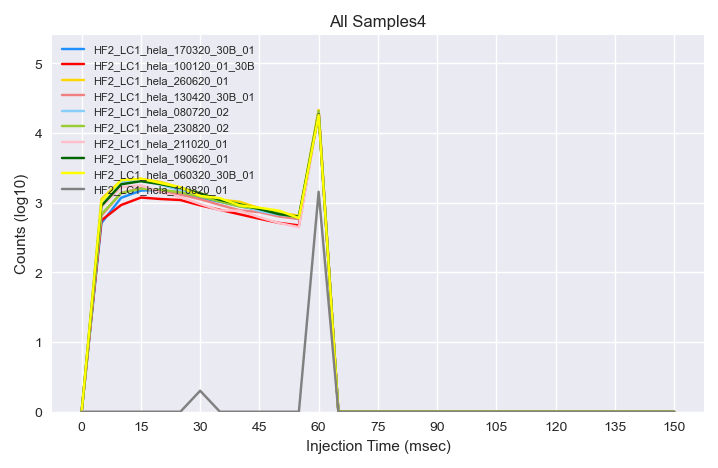

Supplement: Supplementary file 2 — pr0c00956_si_003.zip [file pr0c00956_si_003.zip › 100_samples_run1.0/resources/images/all-samples4-ms2-inject.png]

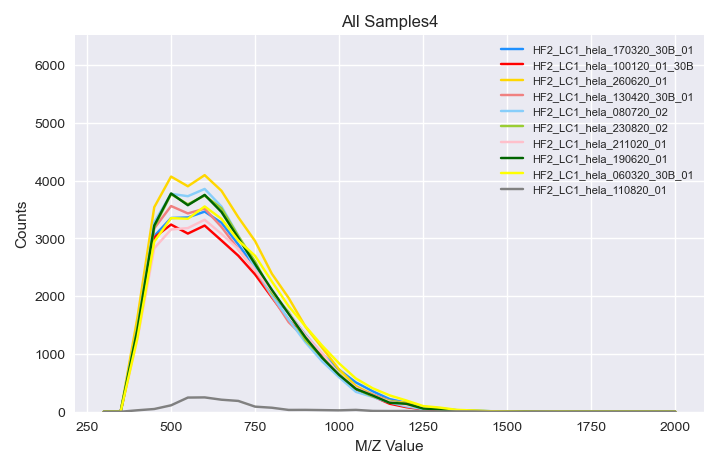

Supplement: Supplementary file 2 — pr0c00956_si_003.zip [file pr0c00956_si_003.zip › 100_samples_run1.0/resources/images/all-samples4-ms2-mz-value.png]

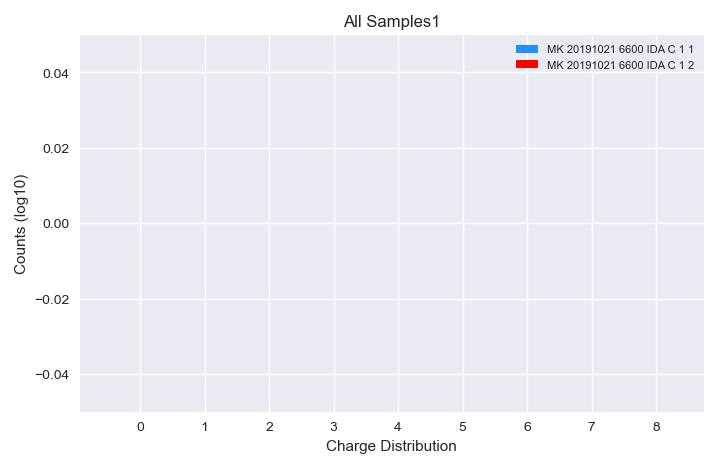

Supplement: Supplementary file 3 — pr0c00956_si_004.zip [file pr0c00956_si_004.zip › ABSciex_data/resources/images/all-samples1-charge-state.png]

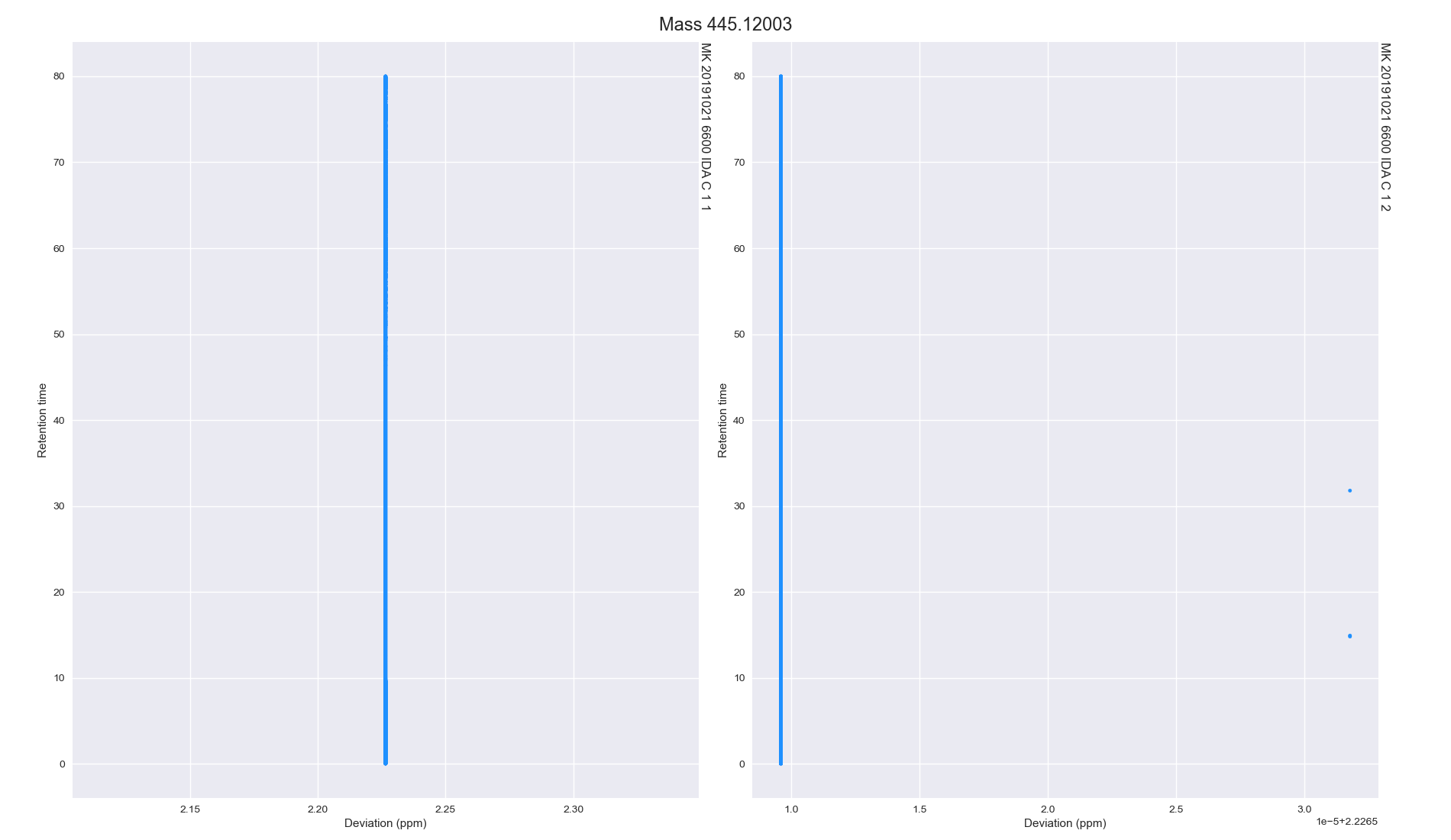

Supplement: Supplementary file 3 — pr0c00956_si_004.zip [file pr0c00956_si_004.zip › ABSciex_data/resources/images/all-samples1-mass1-deviation.png]

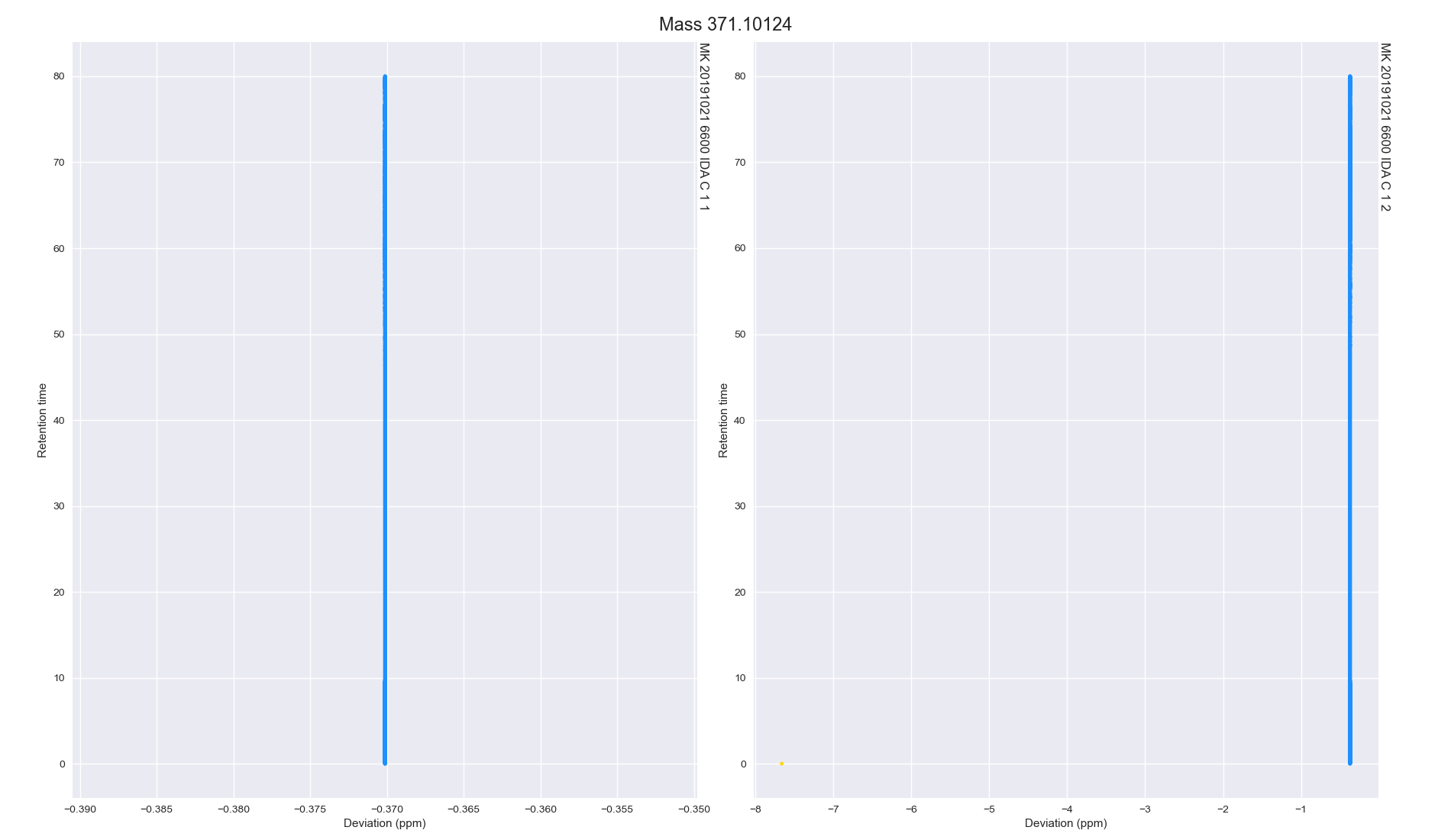

Supplement: Supplementary file 3 — pr0c00956_si_004.zip [file pr0c00956_si_004.zip › ABSciex_data/resources/images/all-samples1-mass2-deviation.png]

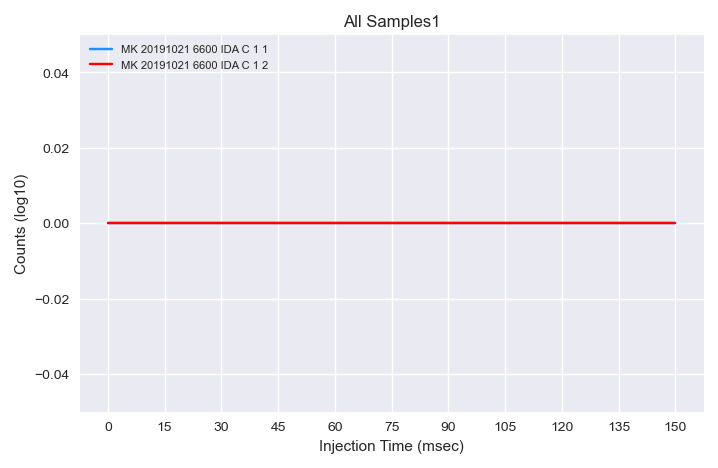

Supplement: Supplementary file 3 — pr0c00956_si_004.zip [file pr0c00956_si_004.zip › ABSciex_data/resources/images/all-samples1-ms2-inject.png]

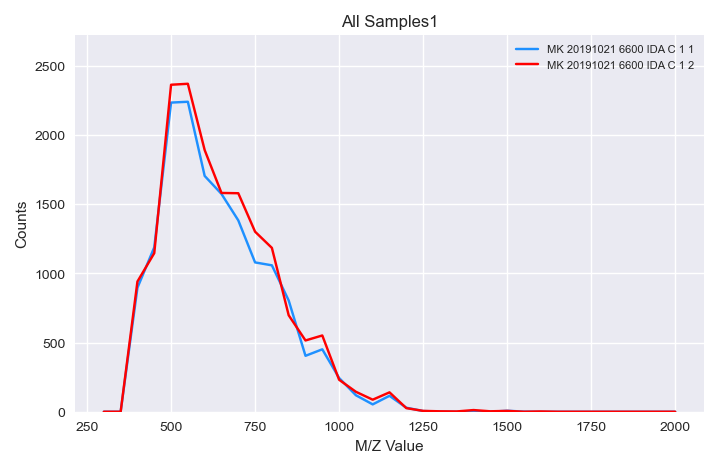

Supplement: Supplementary file 3 — pr0c00956_si_004.zip [file pr0c00956_si_004.zip › ABSciex_data/resources/images/all-samples1-ms2-mz-value.png]

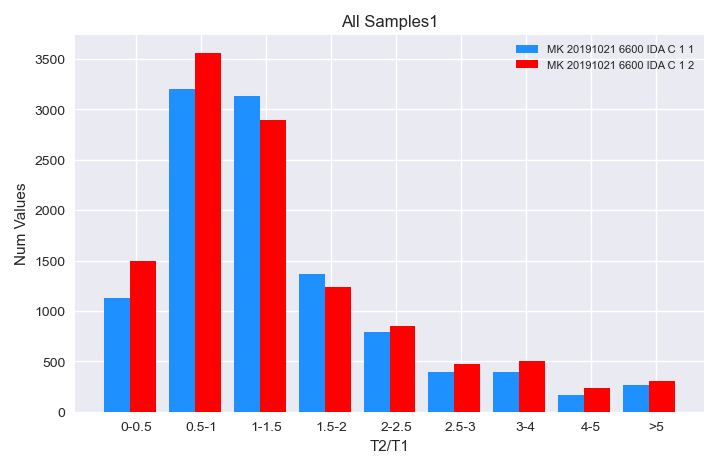

Supplement: Supplementary file 3 — pr0c00956_si_004.zip [file pr0c00956_si_004.zip › ABSciex_data/resources/images/all-samples1-peak-symmetry.png]

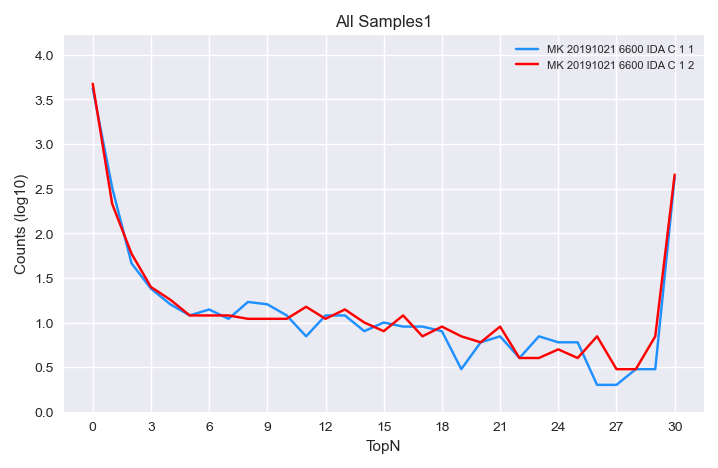

Supplement: Supplementary file 3 — pr0c00956_si_004.zip [file pr0c00956_si_004.zip › ABSciex_data/resources/images/all-samples1-top-n.png]

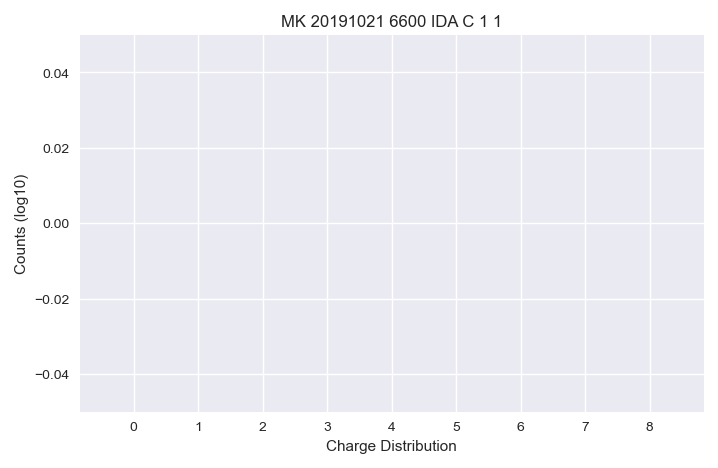

Supplement: Supplementary file 3 — pr0c00956_si_004.zip [file pr0c00956_si_004.zip › ABSciex_data/resources/images/MK 20191021 6600 IDA C 1 1-charge-state.png]

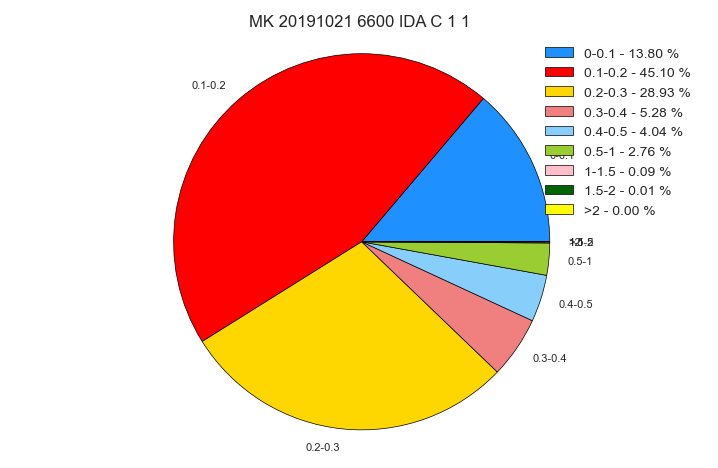

Supplement: Supplementary file 3 — pr0c00956_si_004.zip [file pr0c00956_si_004.zip › ABSciex_data/resources/images/MK 20191021 6600 IDA C 1 1-fmhw-pie.png]

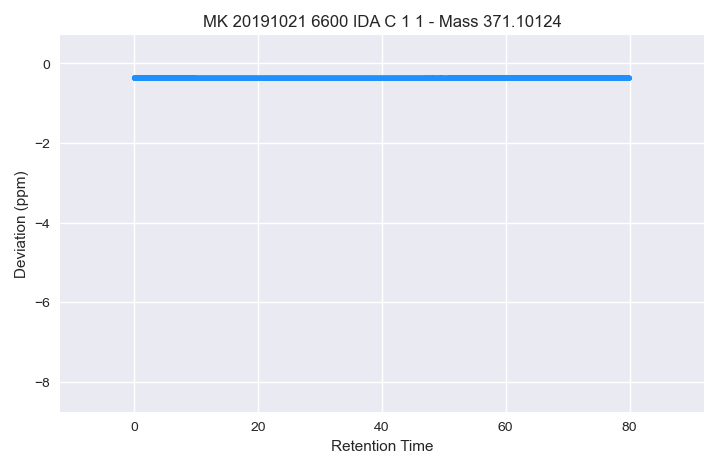

Supplement: Supplementary file 3 — pr0c00956_si_004.zip [file pr0c00956_si_004.zip › ABSciex_data/resources/images/MK 20191021 6600 IDA C 1 1-mass-deviation1.png]

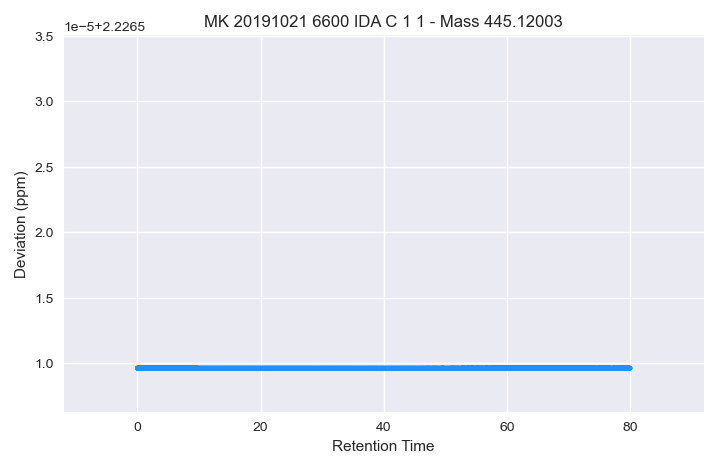

Supplement: Supplementary file 3 — pr0c00956_si_004.zip [file pr0c00956_si_004.zip › ABSciex_data/resources/images/MK 20191021 6600 IDA C 1 1-mass-deviation2.png]

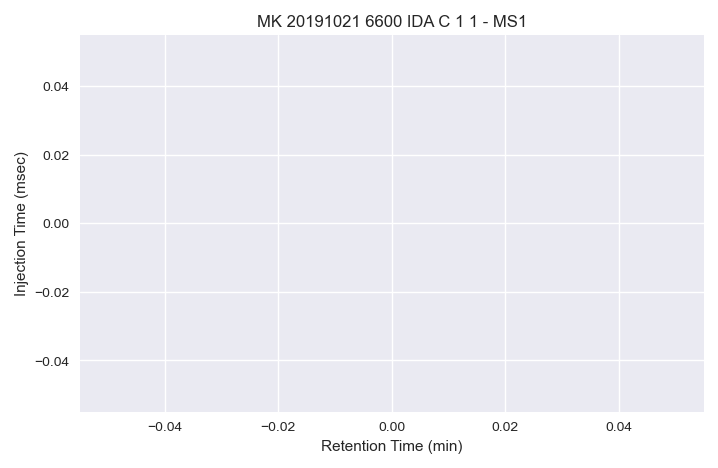

Supplement: Supplementary file 3 — pr0c00956_si_004.zip [file pr0c00956_si_004.zip › ABSciex_data/resources/images/MK 20191021 6600 IDA C 1 1-ms1-inject-vs-ret.png]

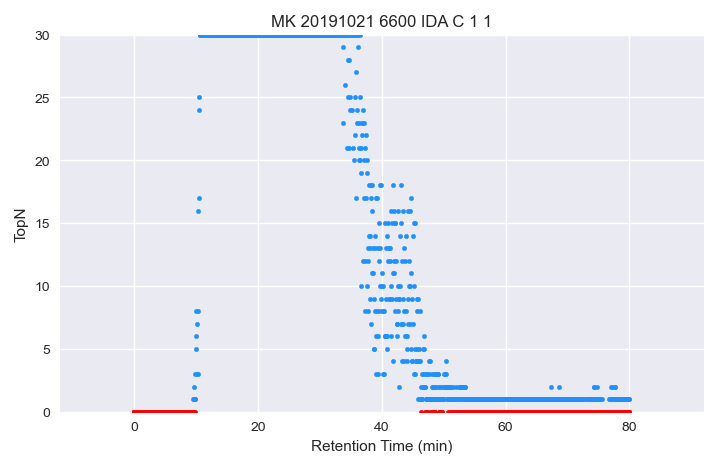

Supplement: Supplementary file 3 — pr0c00956_si_004.zip [file pr0c00956_si_004.zip › ABSciex_data/resources/images/MK 20191021 6600 IDA C 1 1-ms1-ret-vs-top-n.png]

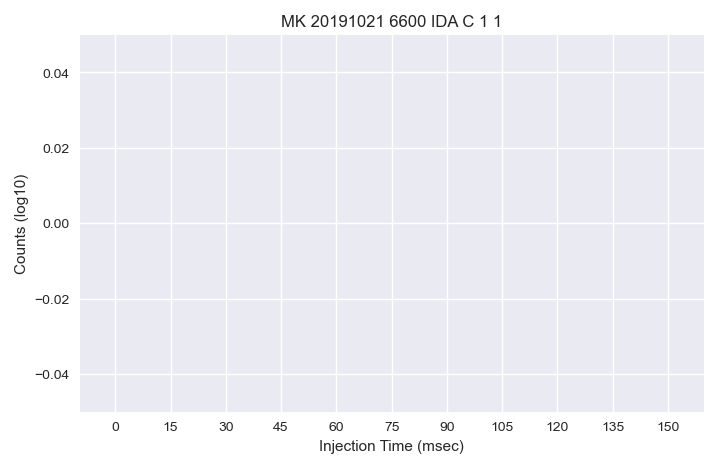

Supplement: Supplementary file 3 — pr0c00956_si_004.zip [file pr0c00956_si_004.zip › ABSciex_data/resources/images/MK 20191021 6600 IDA C 1 1-ms2-inject.png]

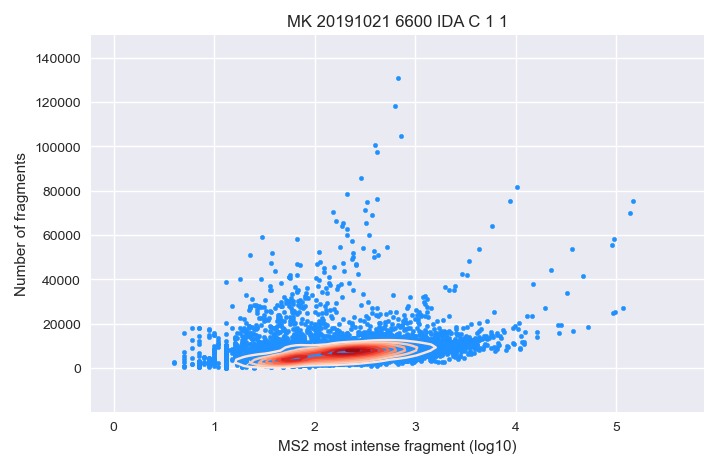

Supplement: Supplementary file 3 — pr0c00956_si_004.zip [file pr0c00956_si_004.zip › ABSciex_data/resources/images/MK 20191021 6600 IDA C 1 1-ms2-max-log-intensity-vs-ms2-num-intensities.png]

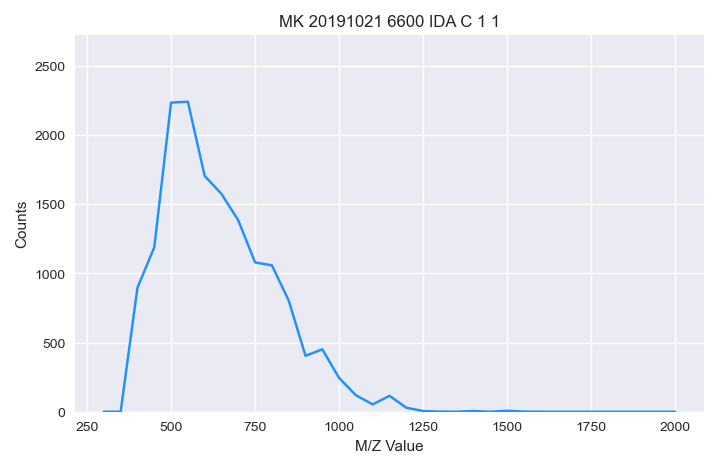

Supplement: Supplementary file 3 — pr0c00956_si_004.zip [file pr0c00956_si_004.zip › ABSciex_data/resources/images/MK 20191021 6600 IDA C 1 1-ms2-mz-value.png]

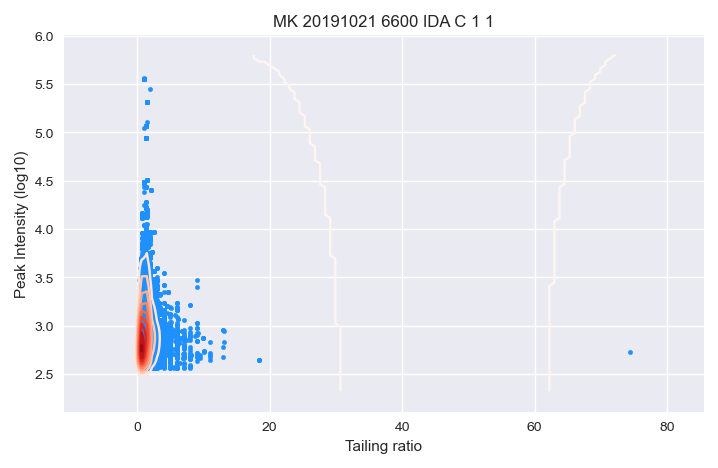

Supplement: Supplementary file 3 — pr0c00956_si_004.zip [file pr0c00956_si_004.zip › ABSciex_data/resources/images/MK 20191021 6600 IDA C 1 1-peak-intentsity-vs-t2-t1-ratio.png]

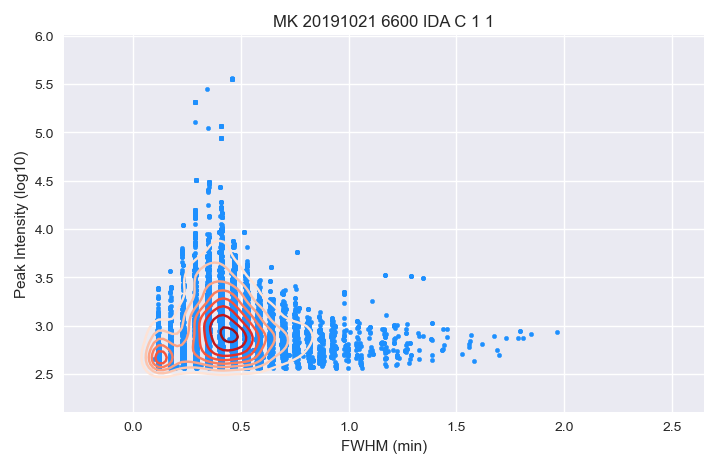

Supplement: Supplementary file 3 — pr0c00956_si_004.zip [file pr0c00956_si_004.zip › ABSciex_data/resources/images/MK 20191021 6600 IDA C 1 1-peak-intentsity-vs-t-sum.png]

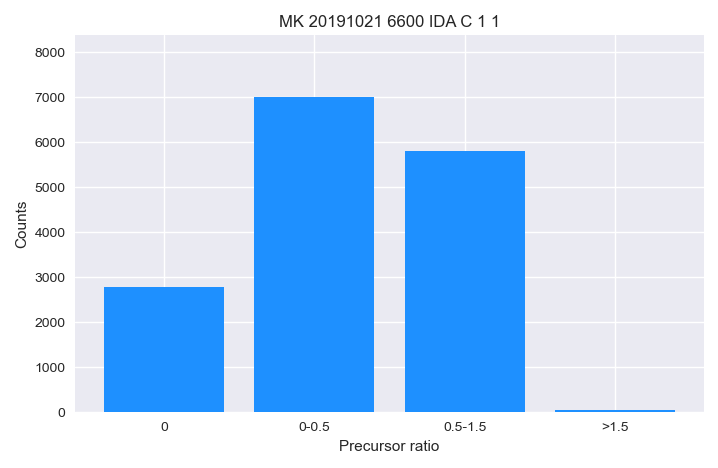

Supplement: Supplementary file 3 — pr0c00956_si_004.zip [file pr0c00956_si_004.zip › ABSciex_data/resources/images/MK 20191021 6600 IDA C 1 1-prec-ratio.png]

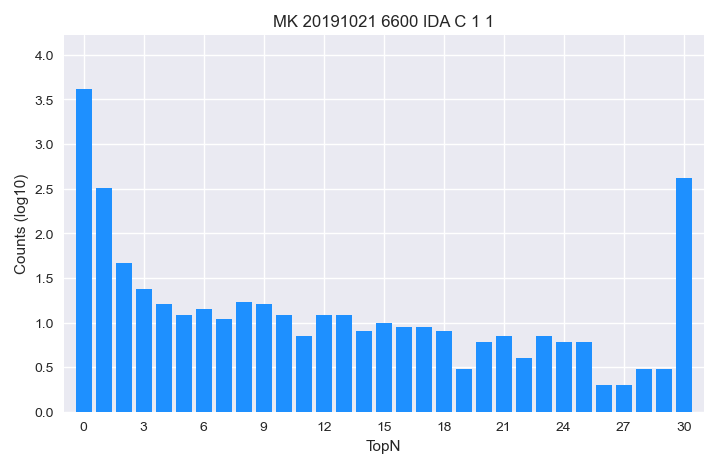

Supplement: Supplementary file 3 — pr0c00956_si_004.zip [file pr0c00956_si_004.zip › ABSciex_data/resources/images/MK 20191021 6600 IDA C 1 1-top-n.png]

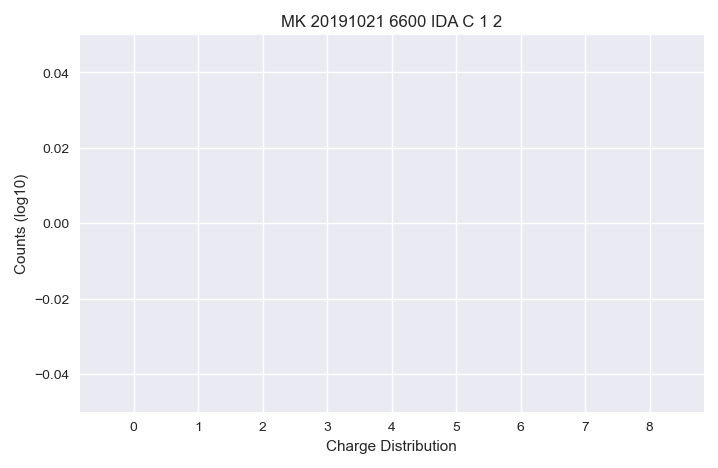

Supplement: Supplementary file 3 — pr0c00956_si_004.zip [file pr0c00956_si_004.zip › ABSciex_data/resources/images/MK 20191021 6600 IDA C 1 2-charge-state.png]

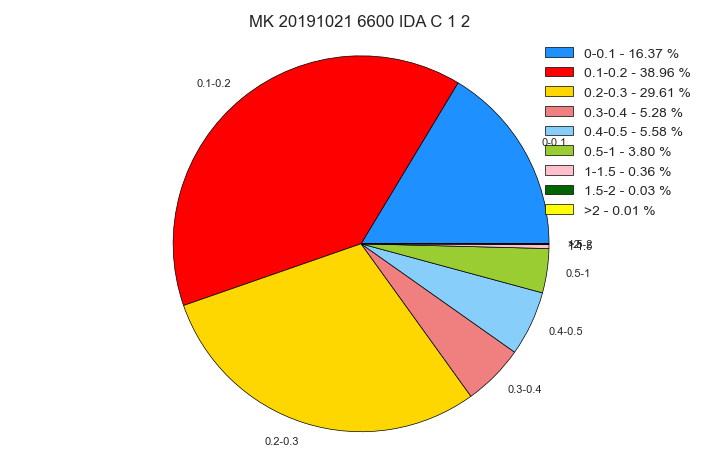

Supplement: Supplementary file 3 — pr0c00956_si_004.zip [file pr0c00956_si_004.zip › ABSciex_data/resources/images/MK 20191021 6600 IDA C 1 2-fmhw-pie.png]

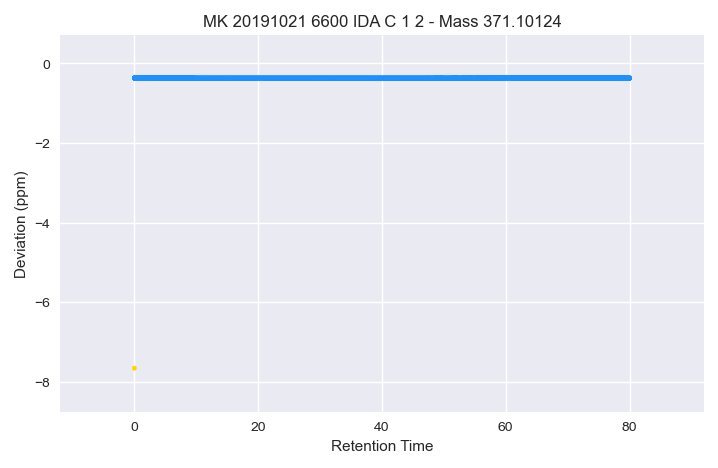

Supplement: Supplementary file 3 — pr0c00956_si_004.zip [file pr0c00956_si_004.zip › ABSciex_data/resources/images/MK 20191021 6600 IDA C 1 2-mass-deviation1.png]

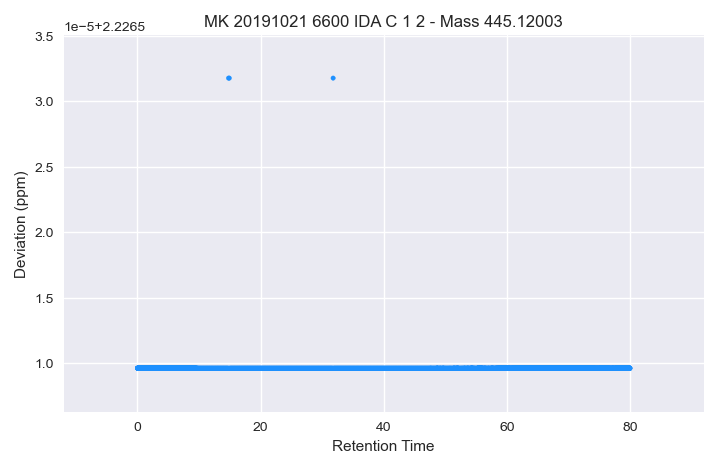

Supplement: Supplementary file 3 — pr0c00956_si_004.zip [file pr0c00956_si_004.zip › ABSciex_data/resources/images/MK 20191021 6600 IDA C 1 2-mass-deviation2.png]

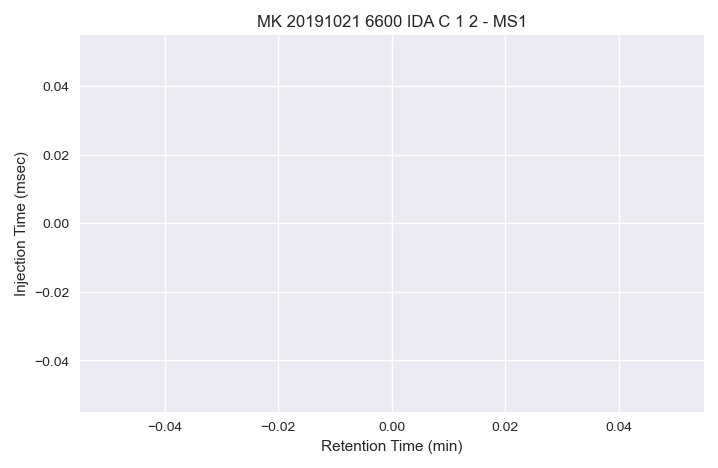

Supplement: Supplementary file 3 — pr0c00956_si_004.zip [file pr0c00956_si_004.zip › ABSciex_data/resources/images/MK 20191021 6600 IDA C 1 2-ms1-inject-vs-ret.png]

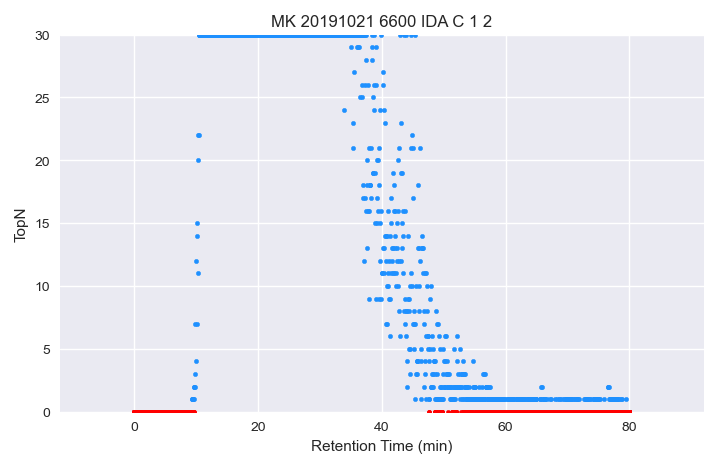

Supplement: Supplementary file 3 — pr0c00956_si_004.zip [file pr0c00956_si_004.zip › ABSciex_data/resources/images/MK 20191021 6600 IDA C 1 2-ms1-ret-vs-top-n.png]

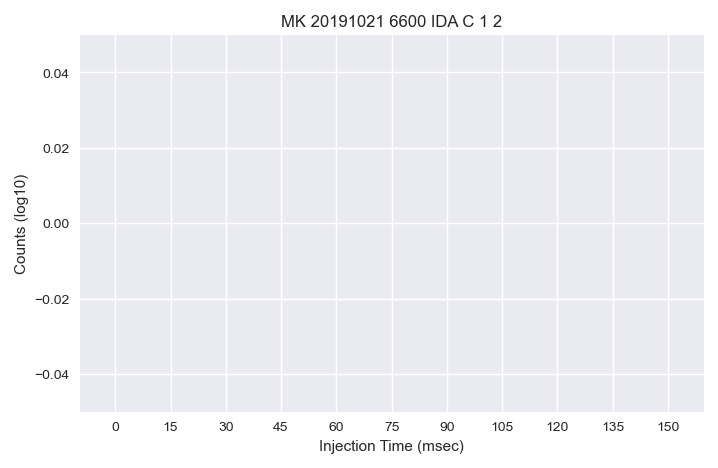

Supplement: Supplementary file 3 — pr0c00956_si_004.zip [file pr0c00956_si_004.zip › ABSciex_data/resources/images/MK 20191021 6600 IDA C 1 2-ms2-inject.png]

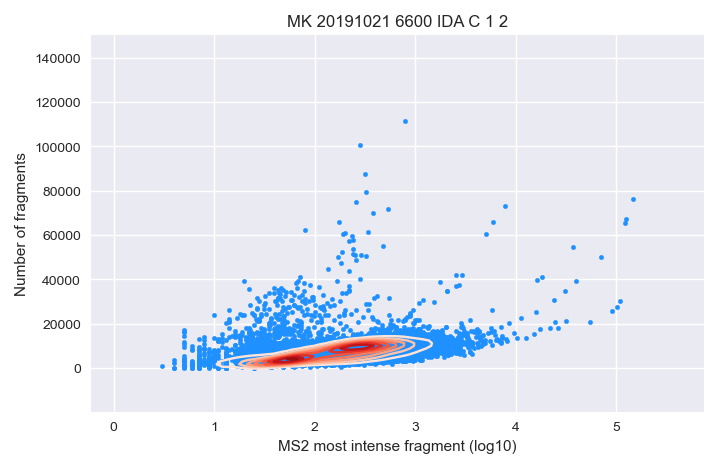

Supplement: Supplementary file 3 — pr0c00956_si_004.zip [file pr0c00956_si_004.zip › ABSciex_data/resources/images/MK 20191021 6600 IDA C 1 2-ms2-max-log-intensity-vs-ms2-num-intensities.png]

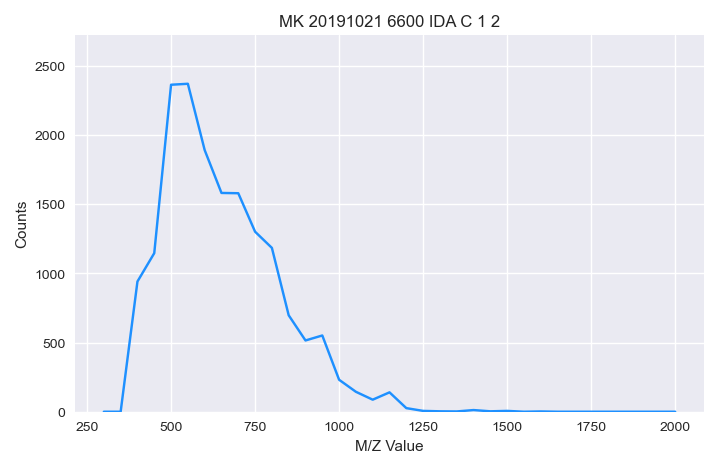

Supplement: Supplementary file 3 — pr0c00956_si_004.zip [file pr0c00956_si_004.zip › ABSciex_data/resources/images/MK 20191021 6600 IDA C 1 2-ms2-mz-value.png]
