# Supplementary figures and images for: RawBeans: A Simple, Vendor-Independent, Raw-Data Quality-Control Tool (part 1 of 3)
Source: J Proteome Res. 2021 Mar 4;20(4):2098–104. doi: 10.1021/acs.jproteome.0c00956 (PMC8041395; doi:10.1021/acs.jproteome.0c00956)

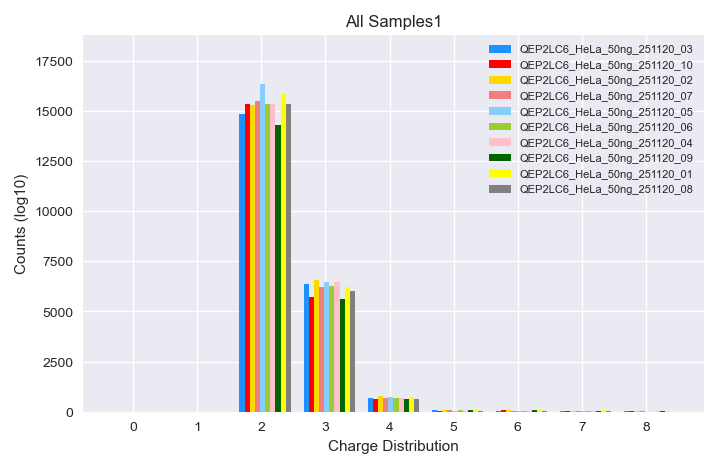

Supplement: Supplementary file 1 — pr0c00956_si_002.zip [file pr0c00956_si_002.zip › RawBeans_report/resources/images/all-samples1-charge-state.png]

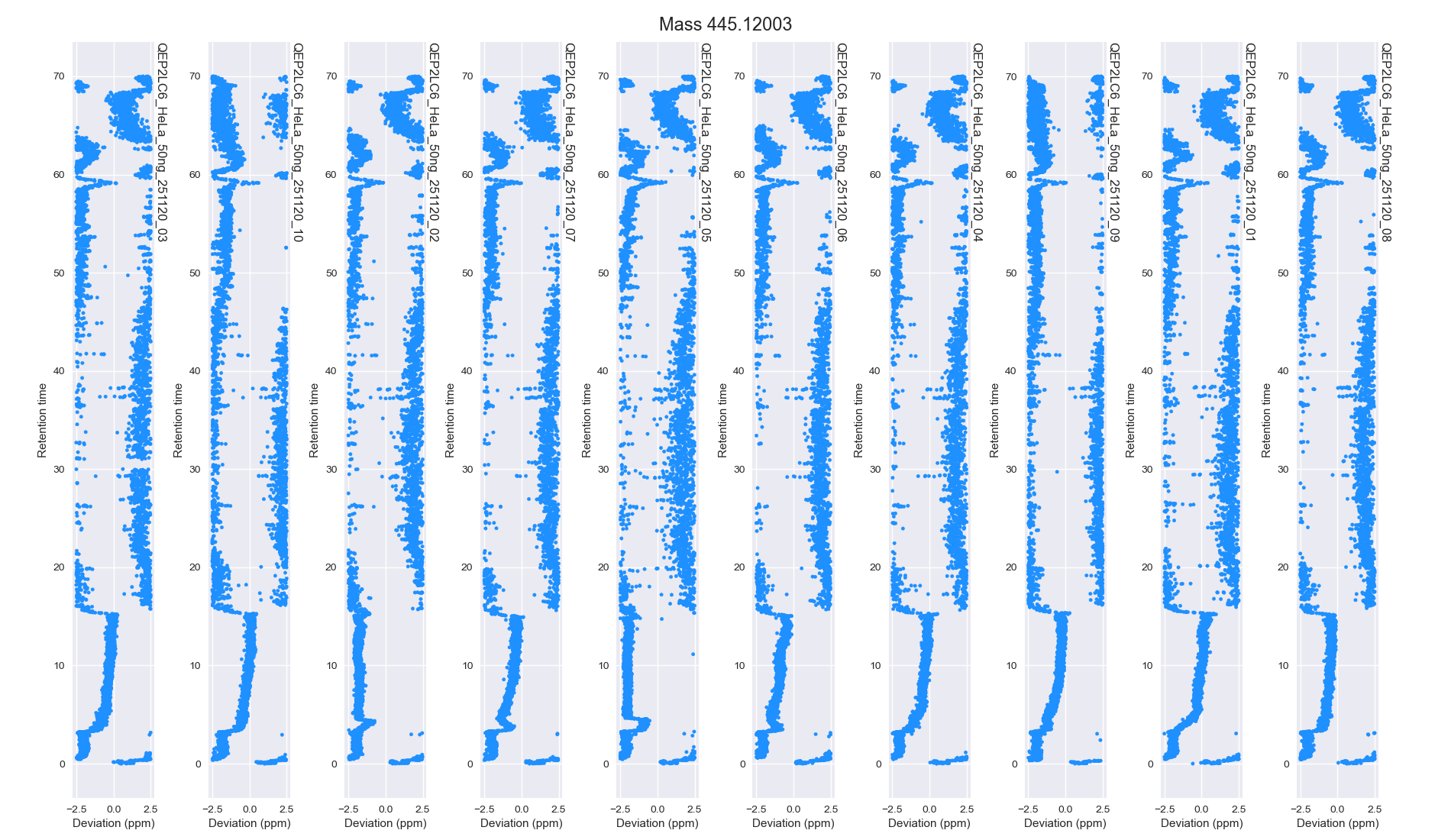

Supplement: Supplementary file 1 — pr0c00956_si_002.zip [file pr0c00956_si_002.zip › RawBeans_report/resources/images/all-samples1-mass1-deviation.png]

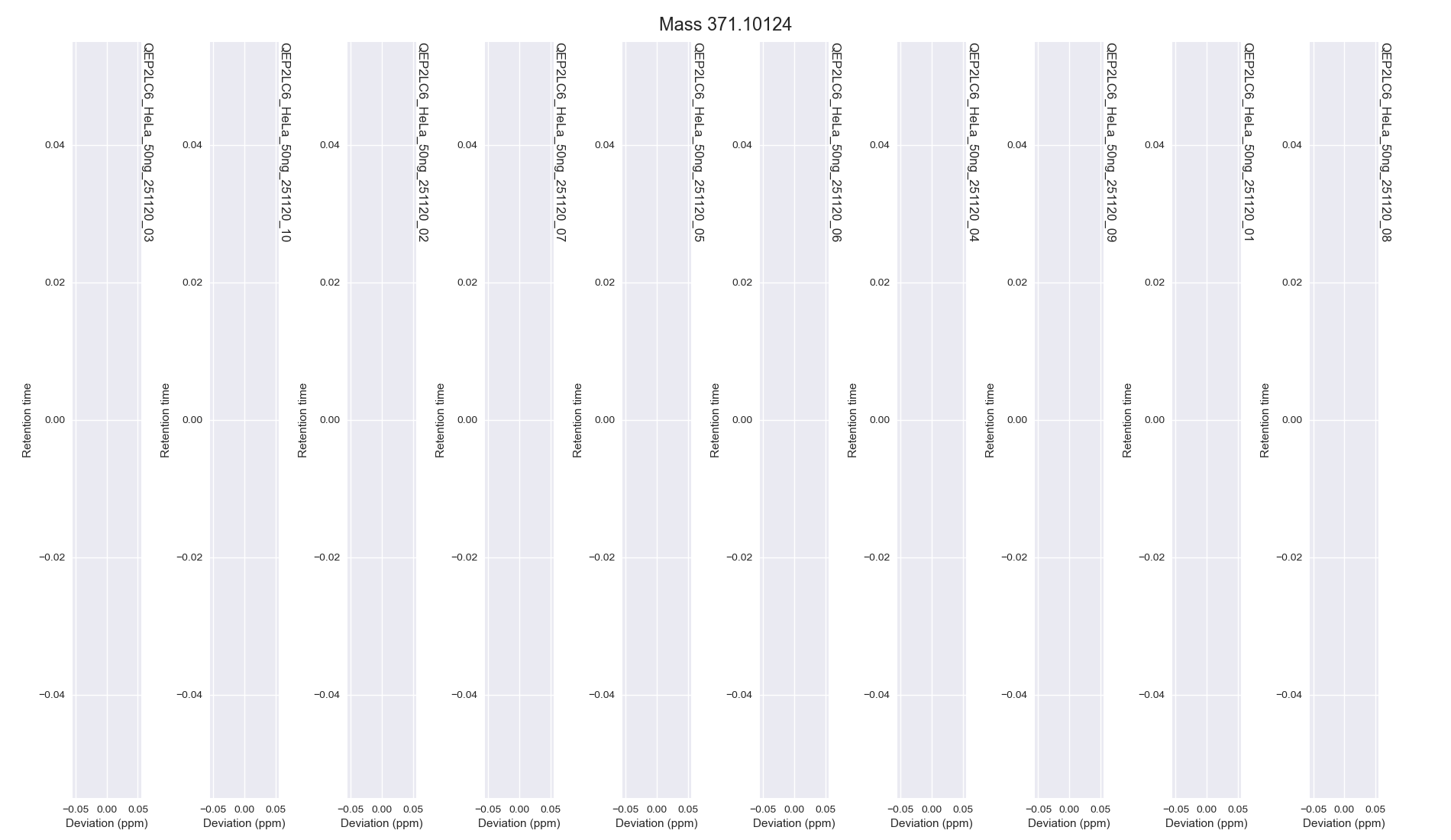

Supplement: Supplementary file 1 — pr0c00956_si_002.zip [file pr0c00956_si_002.zip › RawBeans_report/resources/images/all-samples1-mass2-deviation.png]

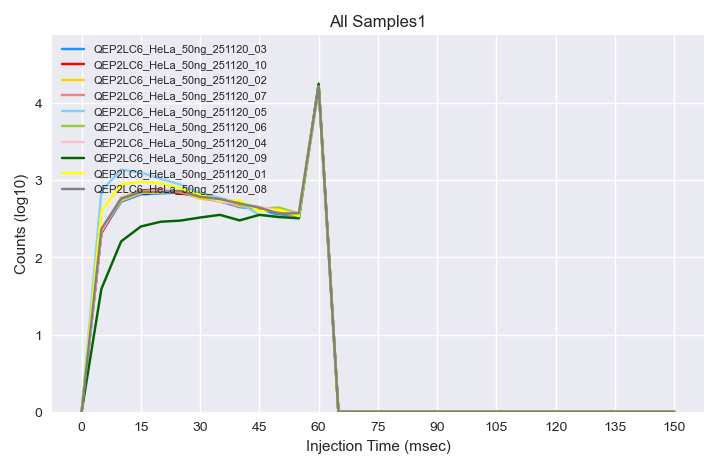

Supplement: Supplementary file 1 — pr0c00956_si_002.zip [file pr0c00956_si_002.zip › RawBeans_report/resources/images/all-samples1-ms2-inject.png]

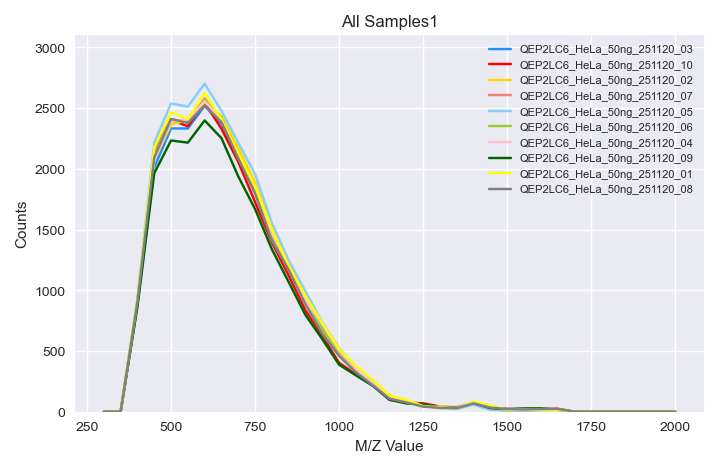

Supplement: Supplementary file 1 — pr0c00956_si_002.zip [file pr0c00956_si_002.zip › RawBeans_report/resources/images/all-samples1-ms2-mz-value.png]

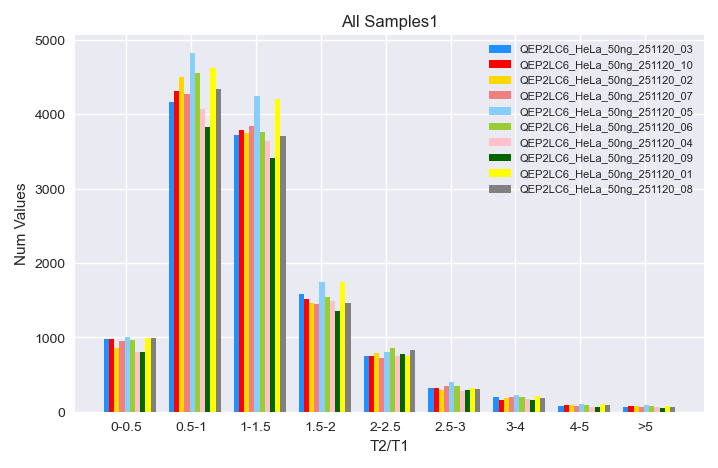

Supplement: Supplementary file 1 — pr0c00956_si_002.zip [file pr0c00956_si_002.zip › RawBeans_report/resources/images/all-samples1-peak-symmetry.png]

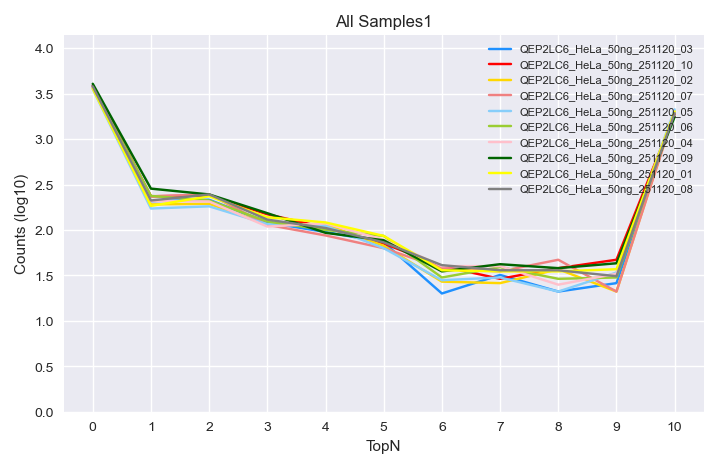

Supplement: Supplementary file 1 — pr0c00956_si_002.zip [file pr0c00956_si_002.zip › RawBeans_report/resources/images/all-samples1-top-n.png]

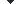

Supplement: Supplementary file 1 — pr0c00956_si_002.zip [file pr0c00956_si_002.zip › RawBeans_report/resources/images/asc.gif]

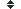

Supplement: Supplementary file 1 — pr0c00956_si_002.zip [file pr0c00956_si_002.zip › RawBeans_report/resources/images/bg.gif]

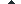

Supplement: Supplementary file 1 — pr0c00956_si_002.zip [file pr0c00956_si_002.zip › RawBeans_report/resources/images/desc.gif]

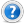

Supplement: Supplementary file 1 — pr0c00956_si_002.zip [file pr0c00956_si_002.zip › RawBeans_report/resources/images/help-icon-24.png]

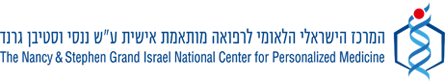

Supplement: Supplementary file 1 — pr0c00956_si_002.zip [file pr0c00956_si_002.zip › RawBeans_report/resources/images/incpm_logo.png]

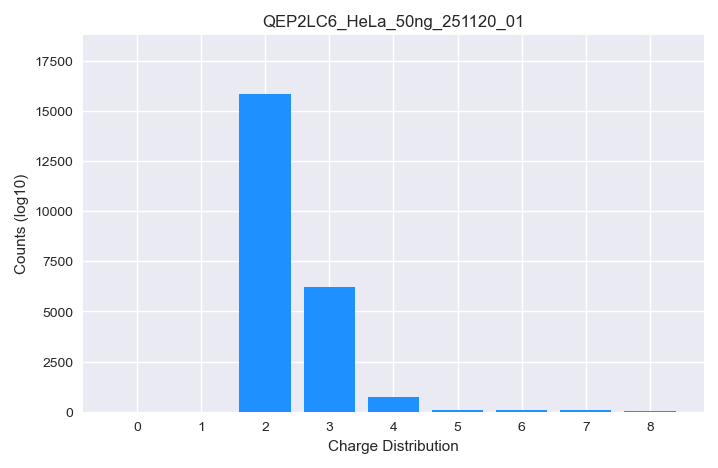

Supplement: Supplementary file 1 — pr0c00956_si_002.zip [file pr0c00956_si_002.zip › RawBeans_report/resources/images/QEP2LC6_HeLa_50ng_251120_01-charge-state.png]

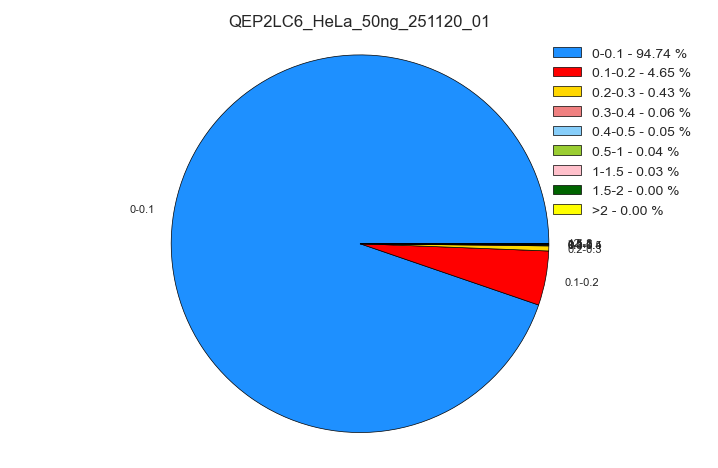

Supplement: Supplementary file 1 — pr0c00956_si_002.zip [file pr0c00956_si_002.zip › RawBeans_report/resources/images/QEP2LC6_HeLa_50ng_251120_01-fmhw-pie.png]

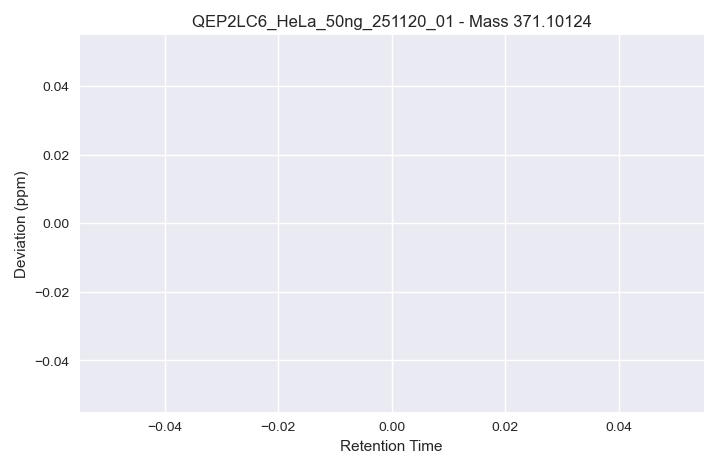

Supplement: Supplementary file 1 — pr0c00956_si_002.zip [file pr0c00956_si_002.zip › RawBeans_report/resources/images/QEP2LC6_HeLa_50ng_251120_01-mass-deviation1.png]

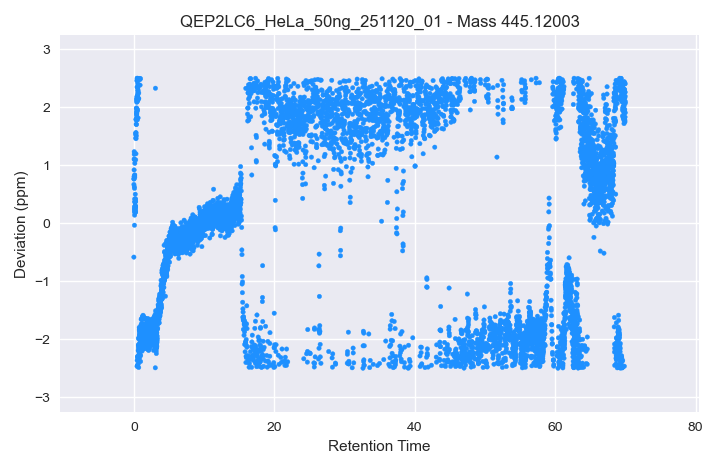

Supplement: Supplementary file 1 — pr0c00956_si_002.zip [file pr0c00956_si_002.zip › RawBeans_report/resources/images/QEP2LC6_HeLa_50ng_251120_01-mass-deviation2.png]

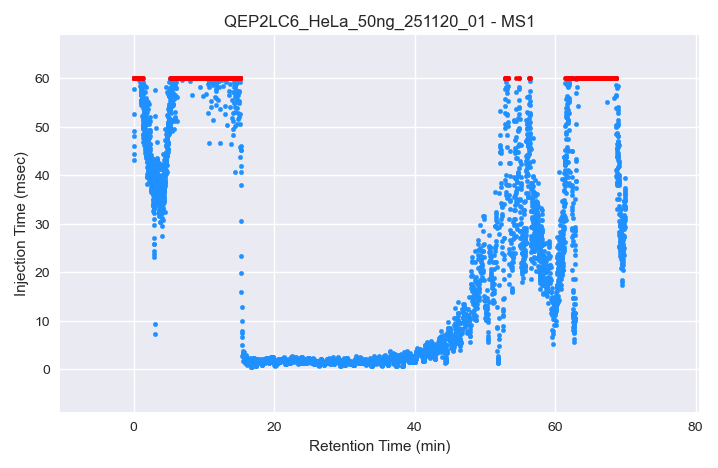

Supplement: Supplementary file 1 — pr0c00956_si_002.zip [file pr0c00956_si_002.zip › RawBeans_report/resources/images/QEP2LC6_HeLa_50ng_251120_01-ms1-inject-vs-ret.png]

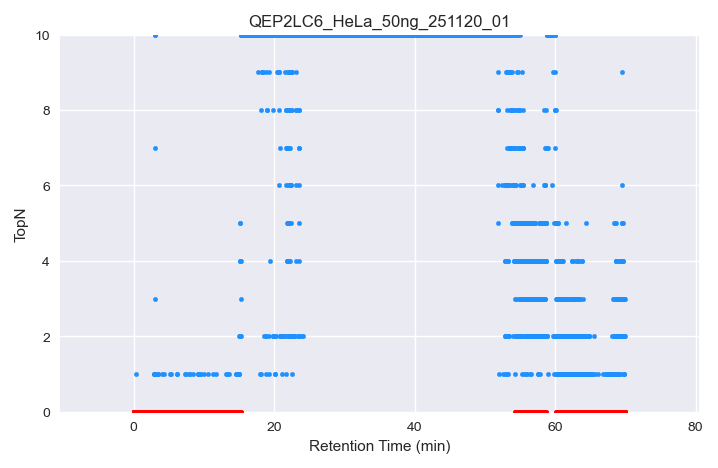

Supplement: Supplementary file 1 — pr0c00956_si_002.zip [file pr0c00956_si_002.zip › RawBeans_report/resources/images/QEP2LC6_HeLa_50ng_251120_01-ms1-ret-vs-top-n.png]

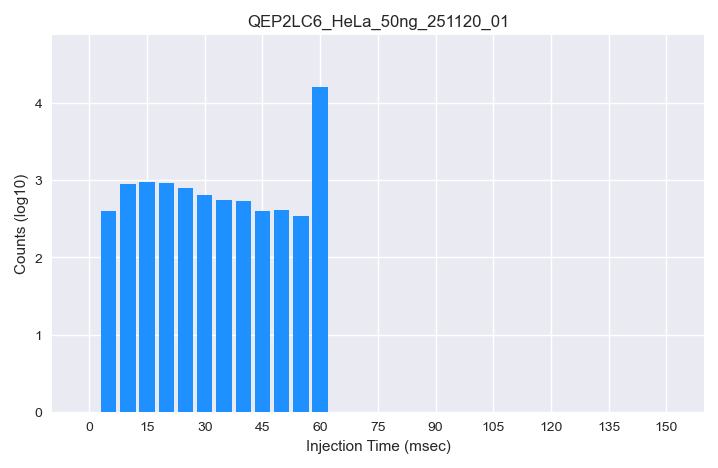

Supplement: Supplementary file 1 — pr0c00956_si_002.zip [file pr0c00956_si_002.zip › RawBeans_report/resources/images/QEP2LC6_HeLa_50ng_251120_01-ms2-inject.png]

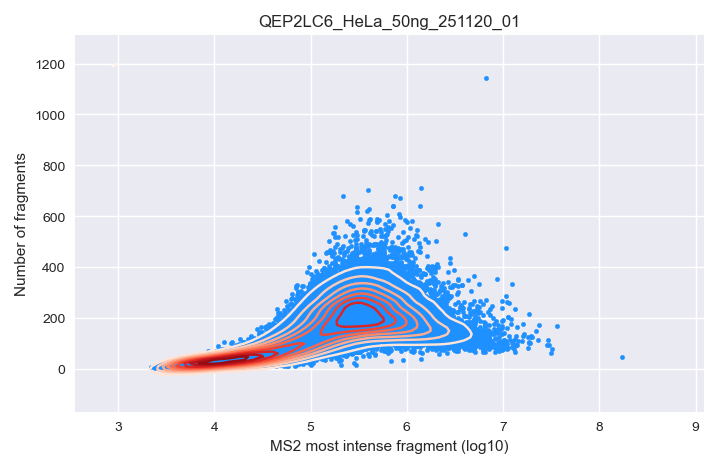

Supplement: Supplementary file 1 — pr0c00956_si_002.zip [file pr0c00956_si_002.zip › RawBeans_report/resources/images/QEP2LC6_HeLa_50ng_251120_01-ms2-max-log-intensity-vs-ms2-num-intensities.png]

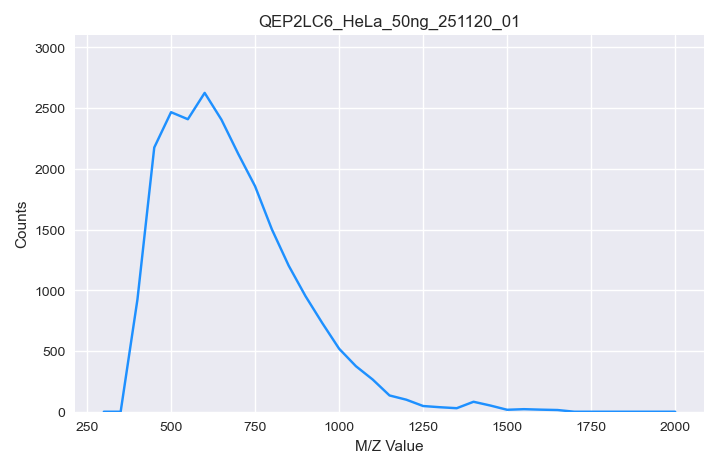

Supplement: Supplementary file 1 — pr0c00956_si_002.zip [file pr0c00956_si_002.zip › RawBeans_report/resources/images/QEP2LC6_HeLa_50ng_251120_01-ms2-mz-value.png]

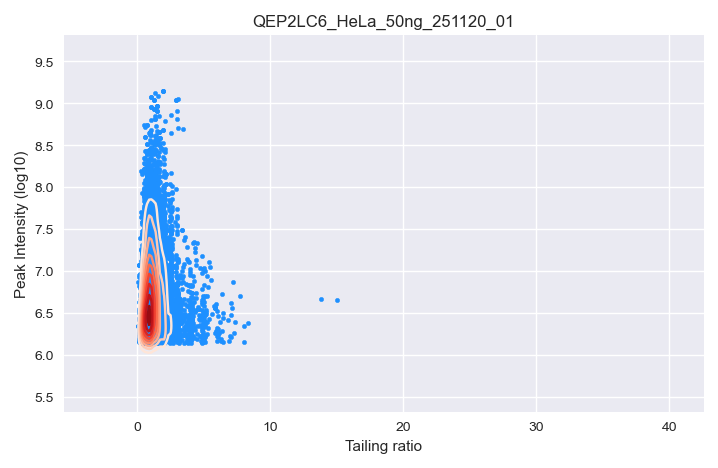

Supplement: Supplementary file 1 — pr0c00956_si_002.zip [file pr0c00956_si_002.zip › RawBeans_report/resources/images/QEP2LC6_HeLa_50ng_251120_01-peak-intentsity-vs-t2-t1-ratio.png]

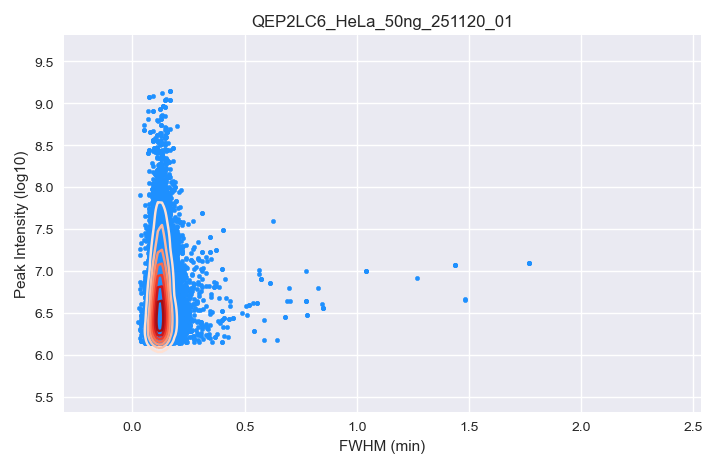

Supplement: Supplementary file 1 — pr0c00956_si_002.zip [file pr0c00956_si_002.zip › RawBeans_report/resources/images/QEP2LC6_HeLa_50ng_251120_01-peak-intentsity-vs-t-sum.png]

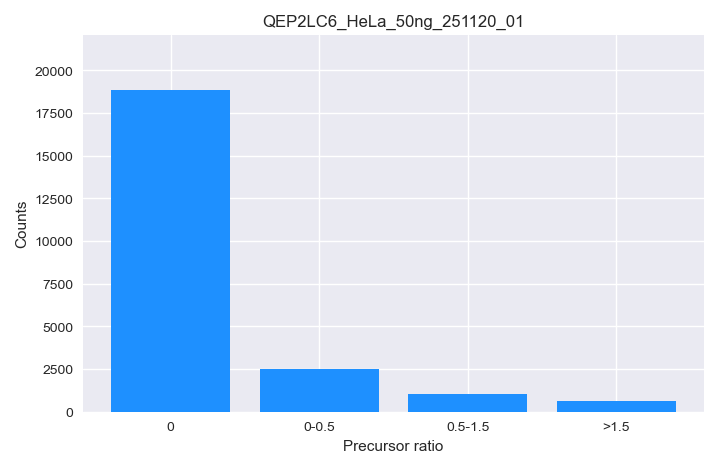

Supplement: Supplementary file 1 — pr0c00956_si_002.zip [file pr0c00956_si_002.zip › RawBeans_report/resources/images/QEP2LC6_HeLa_50ng_251120_01-prec-ratio.png]

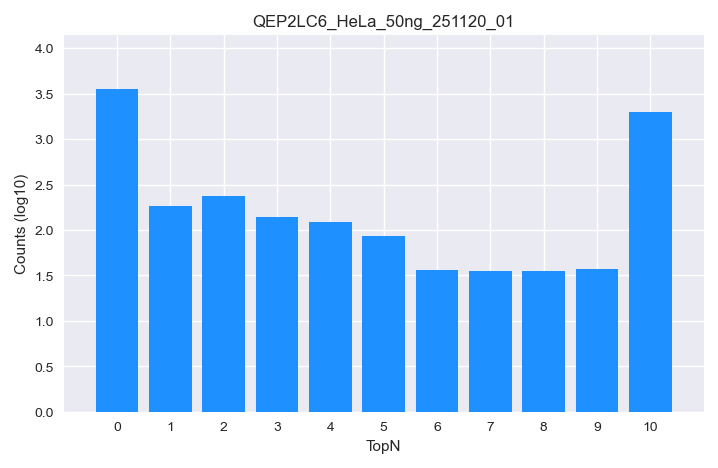

Supplement: Supplementary file 1 — pr0c00956_si_002.zip [file pr0c00956_si_002.zip › RawBeans_report/resources/images/QEP2LC6_HeLa_50ng_251120_01-top-n.png]

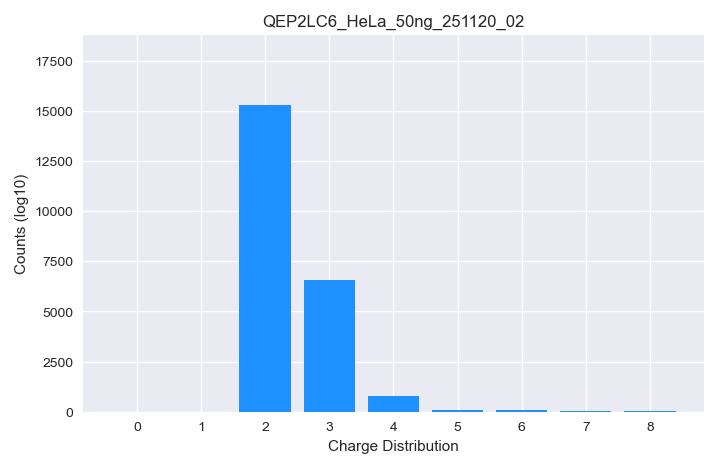

Supplement: Supplementary file 1 — pr0c00956_si_002.zip [file pr0c00956_si_002.zip › RawBeans_report/resources/images/QEP2LC6_HeLa_50ng_251120_02-charge-state.png]

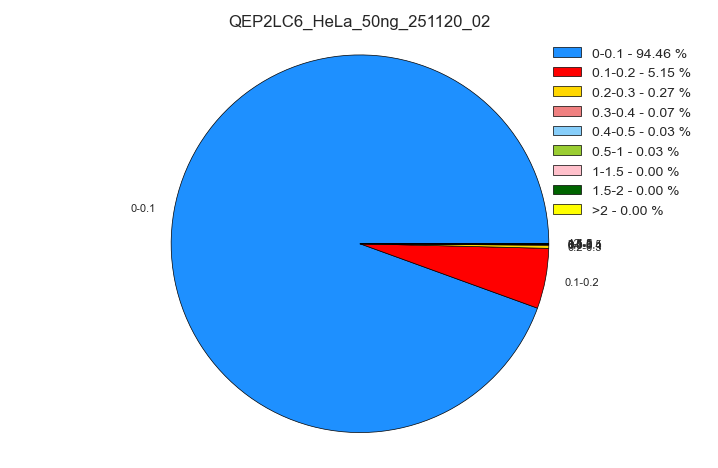

Supplement: Supplementary file 1 — pr0c00956_si_002.zip [file pr0c00956_si_002.zip › RawBeans_report/resources/images/QEP2LC6_HeLa_50ng_251120_02-fmhw-pie.png]

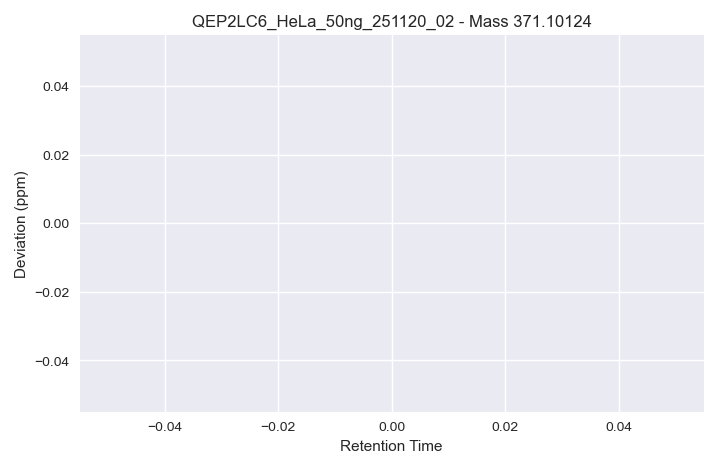

Supplement: Supplementary file 1 — pr0c00956_si_002.zip [file pr0c00956_si_002.zip › RawBeans_report/resources/images/QEP2LC6_HeLa_50ng_251120_02-mass-deviation1.png]

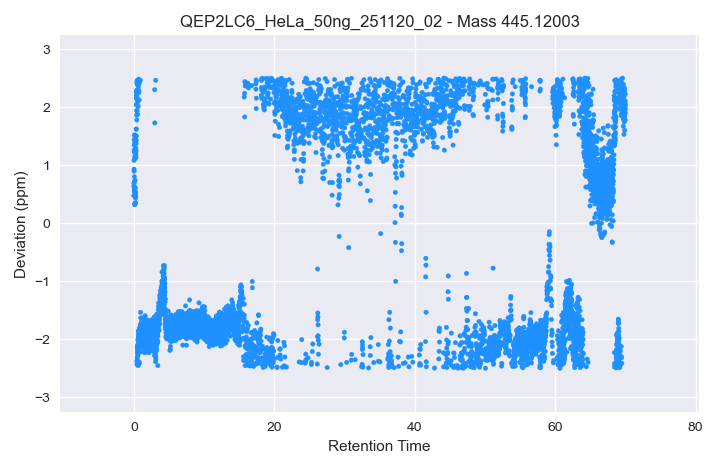

Supplement: Supplementary file 1 — pr0c00956_si_002.zip [file pr0c00956_si_002.zip › RawBeans_report/resources/images/QEP2LC6_HeLa_50ng_251120_02-mass-deviation2.png]

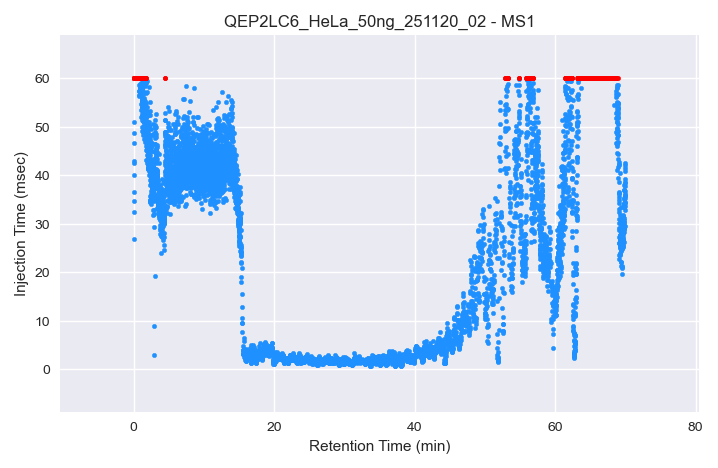

Supplement: Supplementary file 1 — pr0c00956_si_002.zip [file pr0c00956_si_002.zip › RawBeans_report/resources/images/QEP2LC6_HeLa_50ng_251120_02-ms1-inject-vs-ret.png]

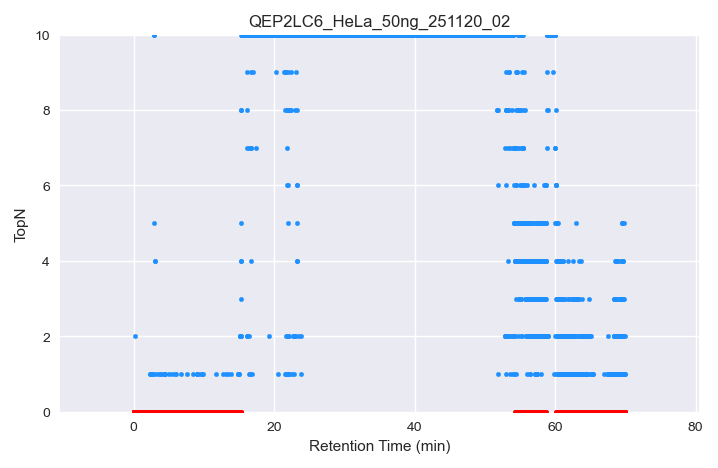

Supplement: Supplementary file 1 — pr0c00956_si_002.zip [file pr0c00956_si_002.zip › RawBeans_report/resources/images/QEP2LC6_HeLa_50ng_251120_02-ms1-ret-vs-top-n.png]

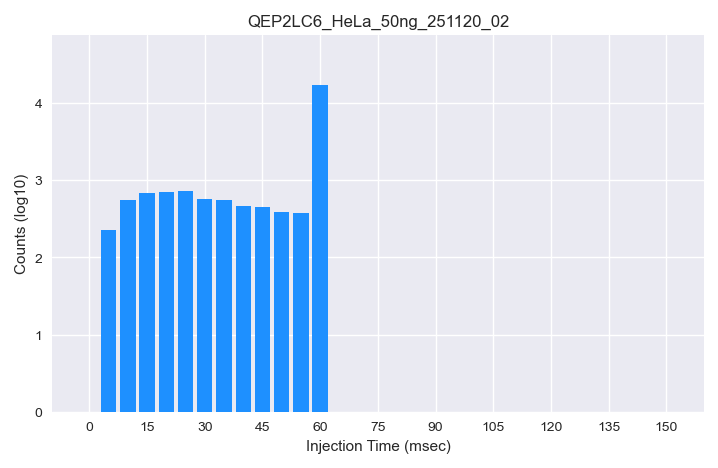

Supplement: Supplementary file 1 — pr0c00956_si_002.zip [file pr0c00956_si_002.zip › RawBeans_report/resources/images/QEP2LC6_HeLa_50ng_251120_02-ms2-inject.png]

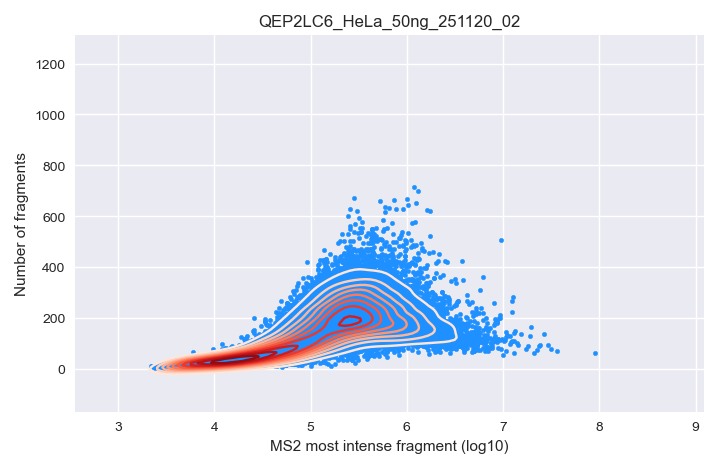

Supplement: Supplementary file 1 — pr0c00956_si_002.zip [file pr0c00956_si_002.zip › RawBeans_report/resources/images/QEP2LC6_HeLa_50ng_251120_02-ms2-max-log-intensity-vs-ms2-num-intensities.png]

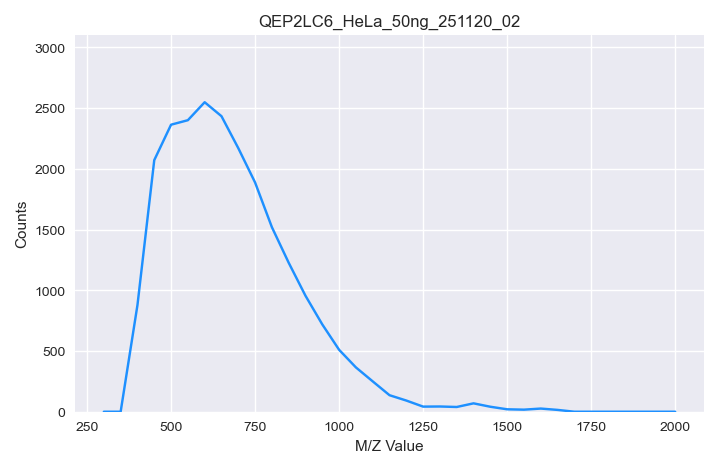

Supplement: Supplementary file 1 — pr0c00956_si_002.zip [file pr0c00956_si_002.zip › RawBeans_report/resources/images/QEP2LC6_HeLa_50ng_251120_02-ms2-mz-value.png]

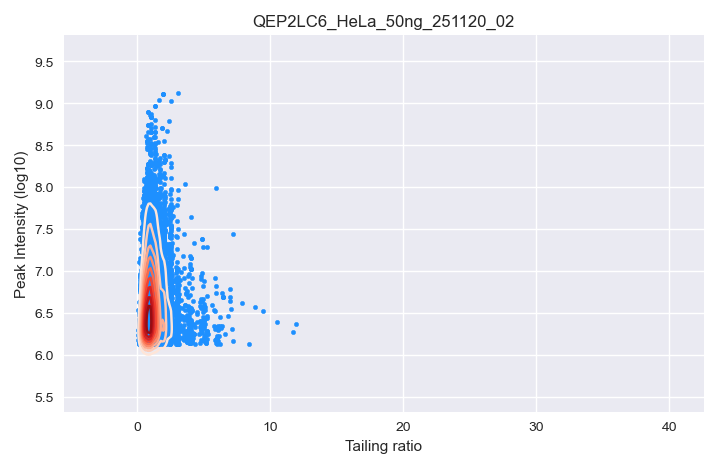

Supplement: Supplementary file 1 — pr0c00956_si_002.zip [file pr0c00956_si_002.zip › RawBeans_report/resources/images/QEP2LC6_HeLa_50ng_251120_02-peak-intentsity-vs-t2-t1-ratio.png]

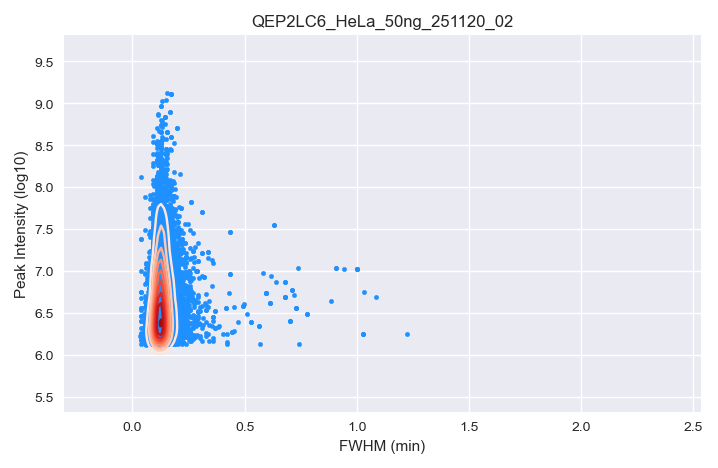

Supplement: Supplementary file 1 — pr0c00956_si_002.zip [file pr0c00956_si_002.zip › RawBeans_report/resources/images/QEP2LC6_HeLa_50ng_251120_02-peak-intentsity-vs-t-sum.png]

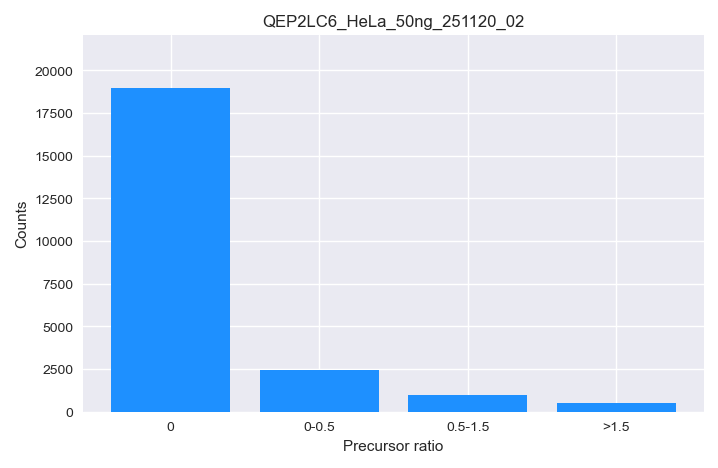

Supplement: Supplementary file 1 — pr0c00956_si_002.zip [file pr0c00956_si_002.zip › RawBeans_report/resources/images/QEP2LC6_HeLa_50ng_251120_02-prec-ratio.png]

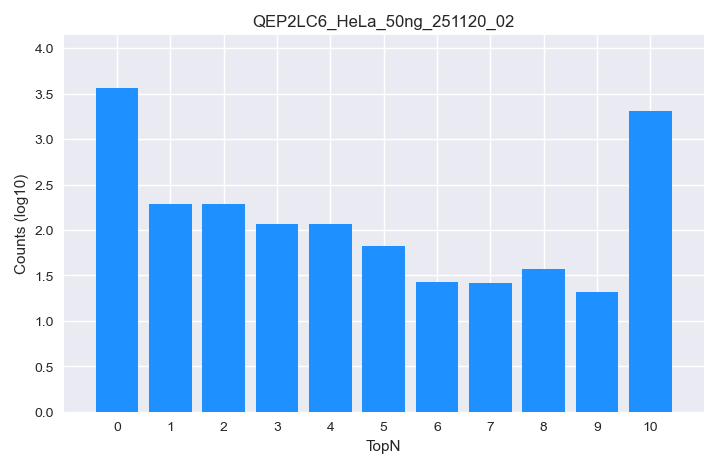

Supplement: Supplementary file 1 — pr0c00956_si_002.zip [file pr0c00956_si_002.zip › RawBeans_report/resources/images/QEP2LC6_HeLa_50ng_251120_02-top-n.png]

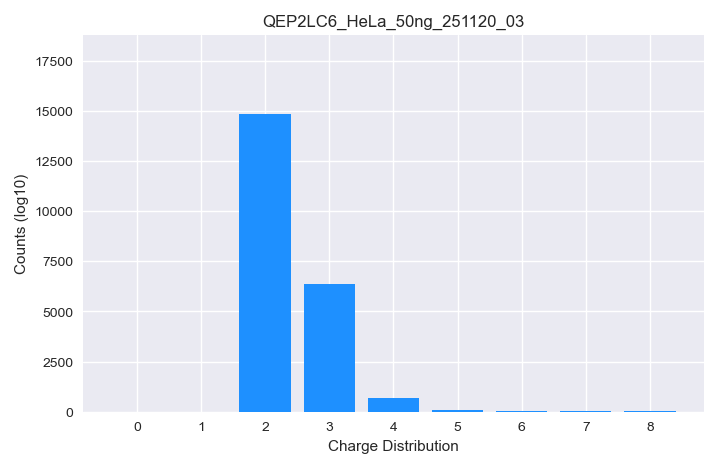

Supplement: Supplementary file 1 — pr0c00956_si_002.zip [file pr0c00956_si_002.zip › RawBeans_report/resources/images/QEP2LC6_HeLa_50ng_251120_03-charge-state.png]

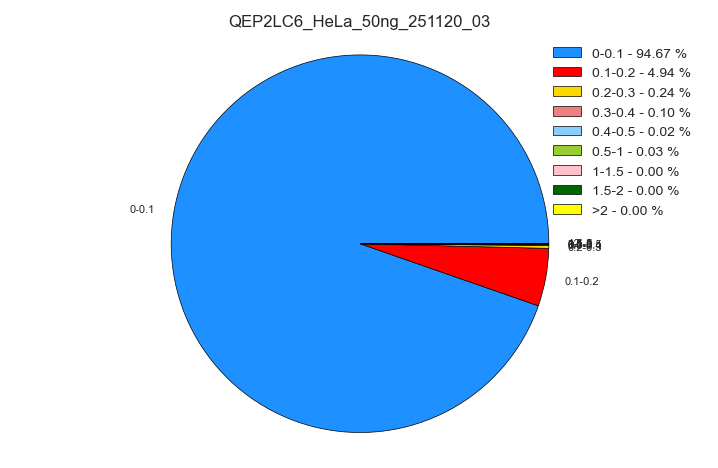

Supplement: Supplementary file 1 — pr0c00956_si_002.zip [file pr0c00956_si_002.zip › RawBeans_report/resources/images/QEP2LC6_HeLa_50ng_251120_03-fmhw-pie.png]

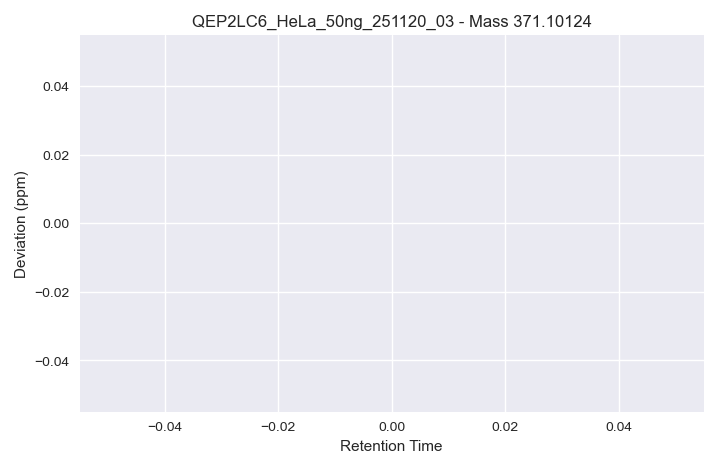

Supplement: Supplementary file 1 — pr0c00956_si_002.zip [file pr0c00956_si_002.zip › RawBeans_report/resources/images/QEP2LC6_HeLa_50ng_251120_03-mass-deviation1.png]

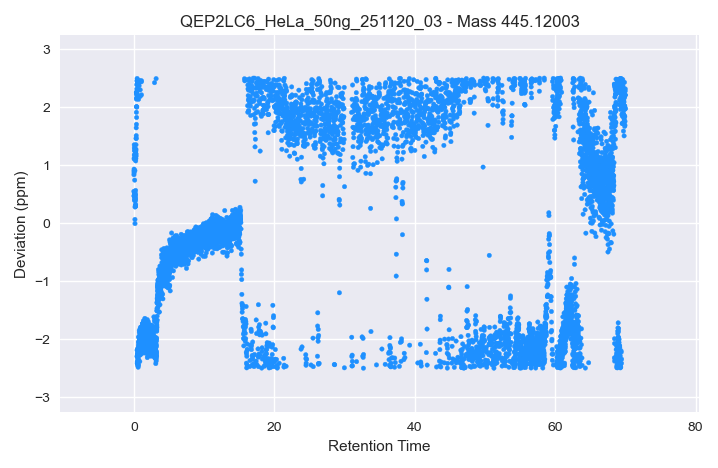

Supplement: Supplementary file 1 — pr0c00956_si_002.zip [file pr0c00956_si_002.zip › RawBeans_report/resources/images/QEP2LC6_HeLa_50ng_251120_03-mass-deviation2.png]

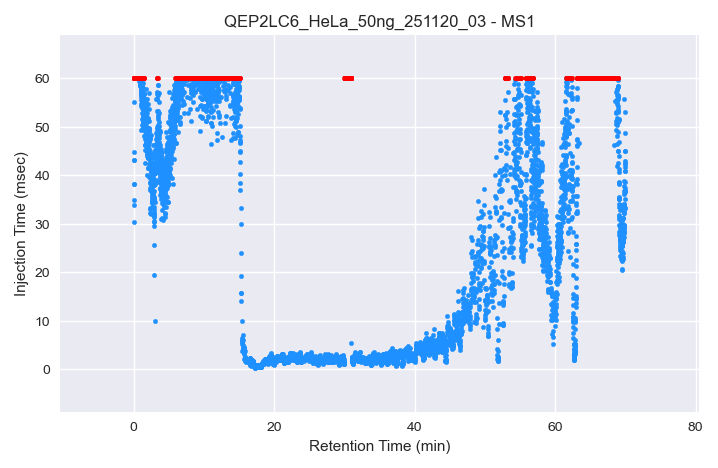

Supplement: Supplementary file 1 — pr0c00956_si_002.zip [file pr0c00956_si_002.zip › RawBeans_report/resources/images/QEP2LC6_HeLa_50ng_251120_03-ms1-inject-vs-ret.png]

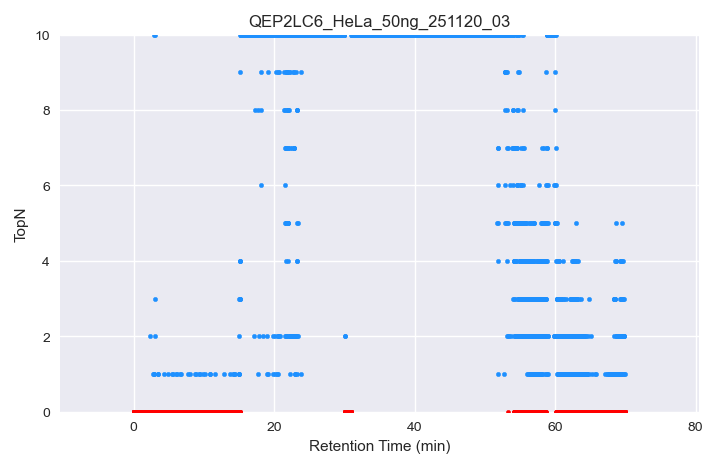

Supplement: Supplementary file 1 — pr0c00956_si_002.zip [file pr0c00956_si_002.zip › RawBeans_report/resources/images/QEP2LC6_HeLa_50ng_251120_03-ms1-ret-vs-top-n.png]

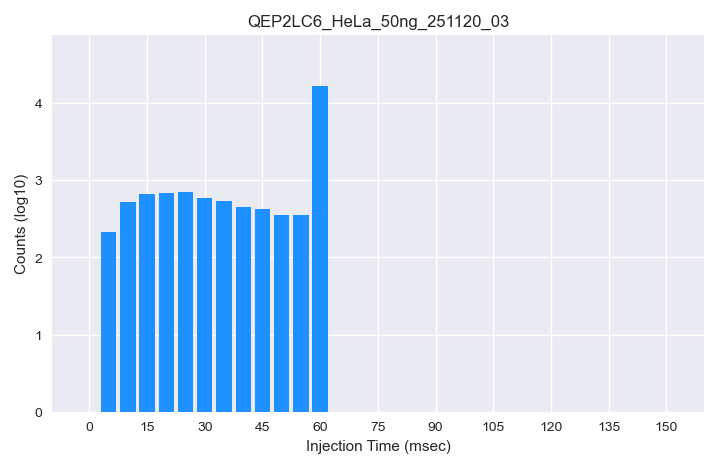

Supplement: Supplementary file 1 — pr0c00956_si_002.zip [file pr0c00956_si_002.zip › RawBeans_report/resources/images/QEP2LC6_HeLa_50ng_251120_03-ms2-inject.png]

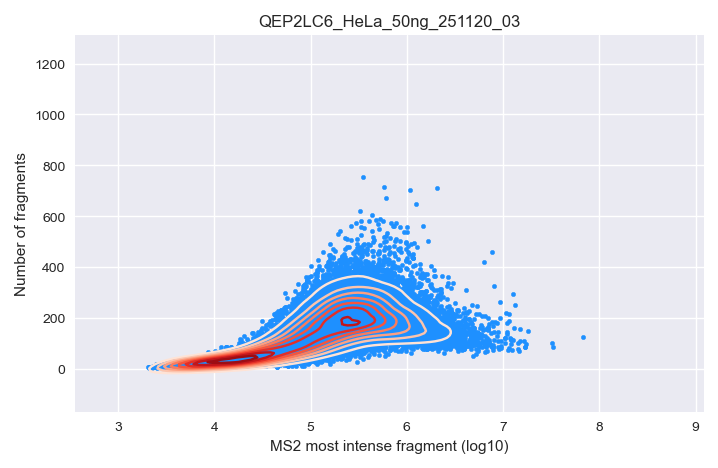

Supplement: Supplementary file 1 — pr0c00956_si_002.zip [file pr0c00956_si_002.zip › RawBeans_report/resources/images/QEP2LC6_HeLa_50ng_251120_03-ms2-max-log-intensity-vs-ms2-num-intensities.png]

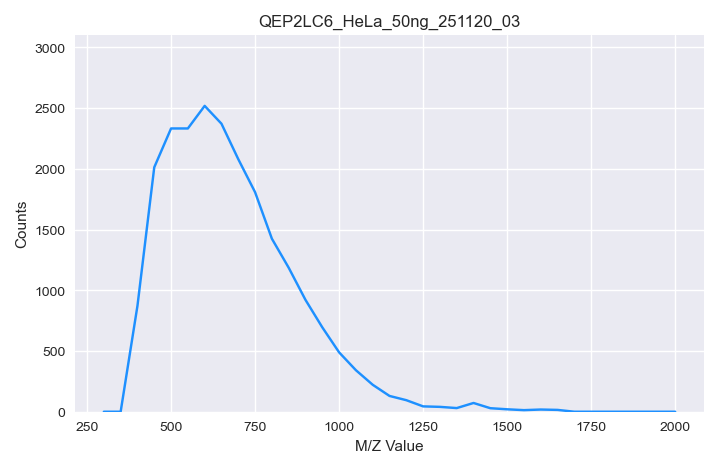

Supplement: Supplementary file 1 — pr0c00956_si_002.zip [file pr0c00956_si_002.zip › RawBeans_report/resources/images/QEP2LC6_HeLa_50ng_251120_03-ms2-mz-value.png]

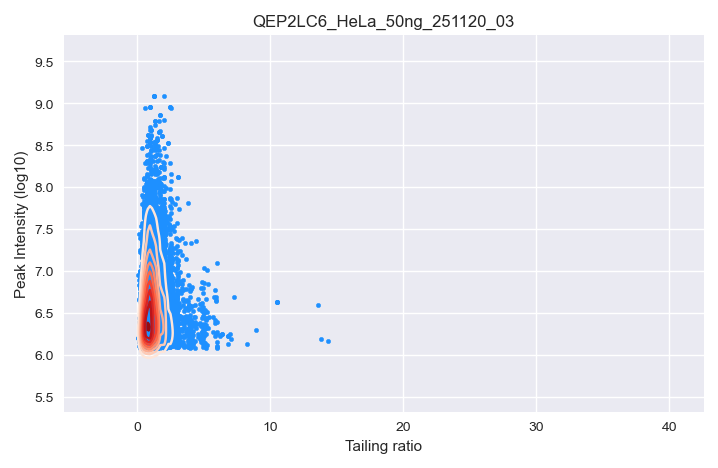

Supplement: Supplementary file 1 — pr0c00956_si_002.zip [file pr0c00956_si_002.zip › RawBeans_report/resources/images/QEP2LC6_HeLa_50ng_251120_03-peak-intentsity-vs-t2-t1-ratio.png]

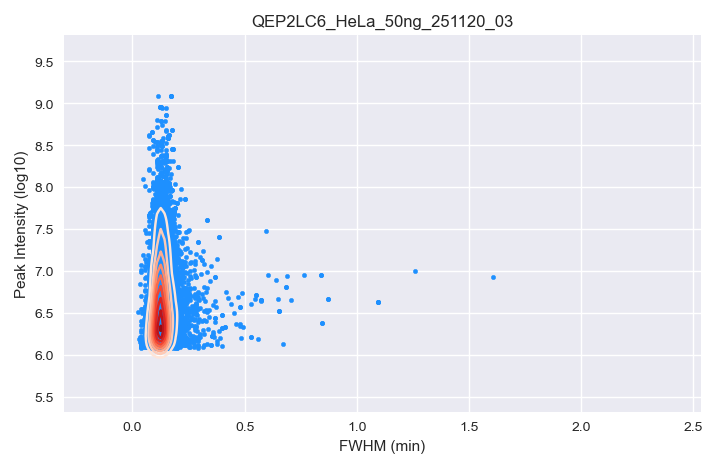

Supplement: Supplementary file 1 — pr0c00956_si_002.zip [file pr0c00956_si_002.zip › RawBeans_report/resources/images/QEP2LC6_HeLa_50ng_251120_03-peak-intentsity-vs-t-sum.png]

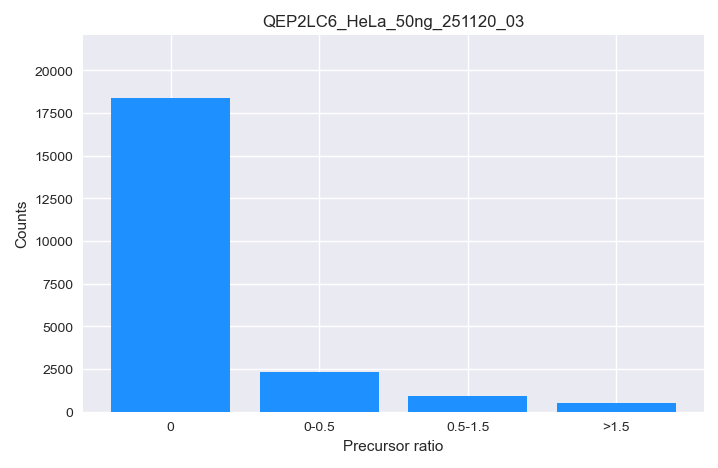

Supplement: Supplementary file 1 — pr0c00956_si_002.zip [file pr0c00956_si_002.zip › RawBeans_report/resources/images/QEP2LC6_HeLa_50ng_251120_03-prec-ratio.png]

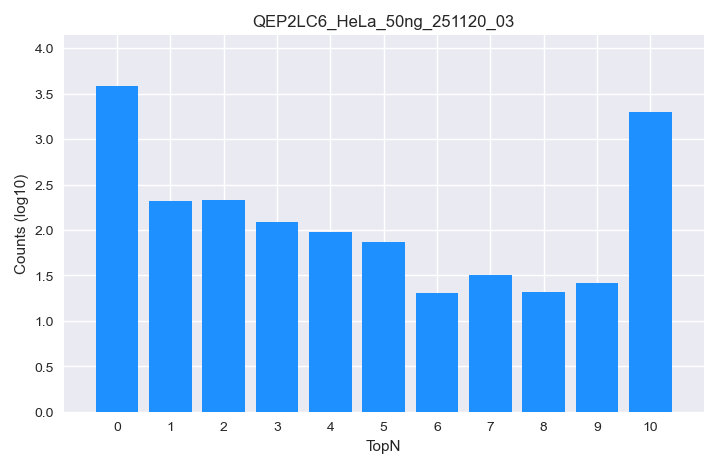

Supplement: Supplementary file 1 — pr0c00956_si_002.zip [file pr0c00956_si_002.zip › RawBeans_report/resources/images/QEP2LC6_HeLa_50ng_251120_03-top-n.png]

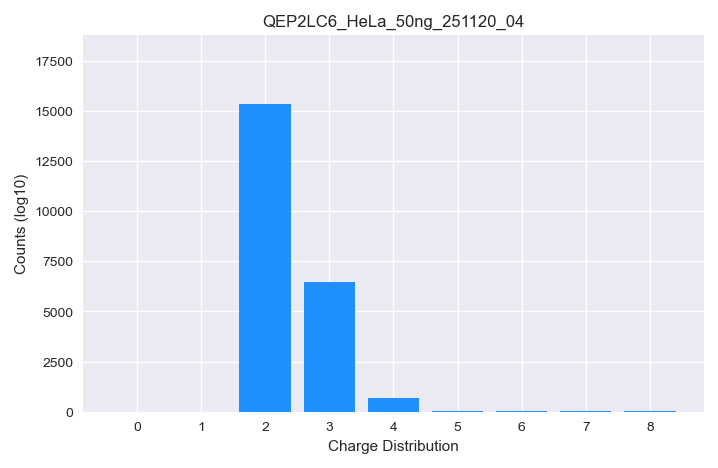

Supplement: Supplementary file 1 — pr0c00956_si_002.zip [file pr0c00956_si_002.zip › RawBeans_report/resources/images/QEP2LC6_HeLa_50ng_251120_04-charge-state.png]

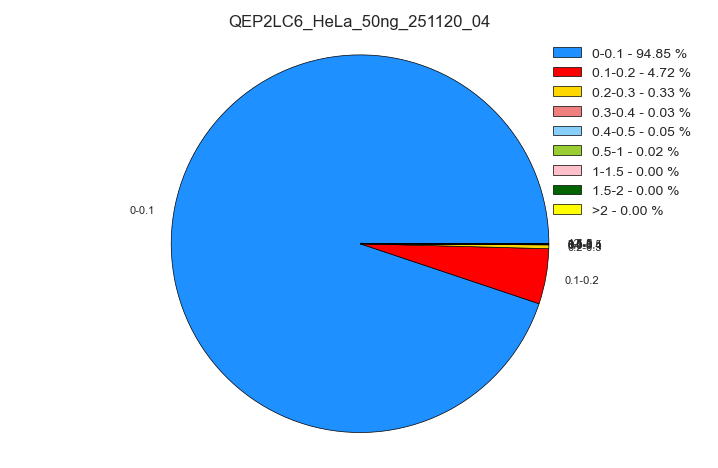

Supplement: Supplementary file 1 — pr0c00956_si_002.zip [file pr0c00956_si_002.zip › RawBeans_report/resources/images/QEP2LC6_HeLa_50ng_251120_04-fmhw-pie.png]

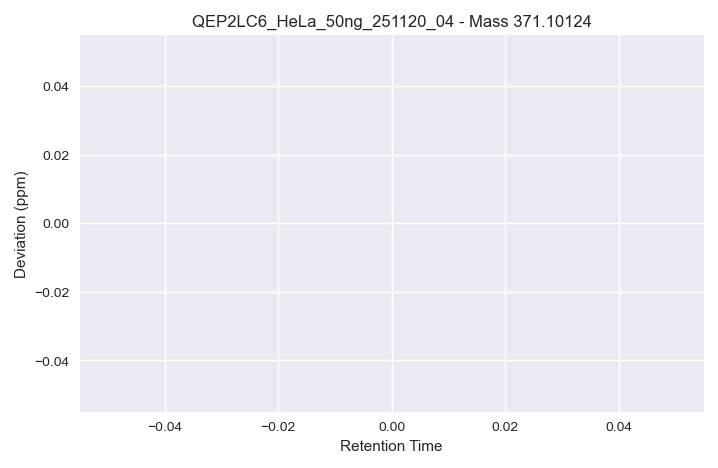

Supplement: Supplementary file 1 — pr0c00956_si_002.zip [file pr0c00956_si_002.zip › RawBeans_report/resources/images/QEP2LC6_HeLa_50ng_251120_04-mass-deviation1.png]

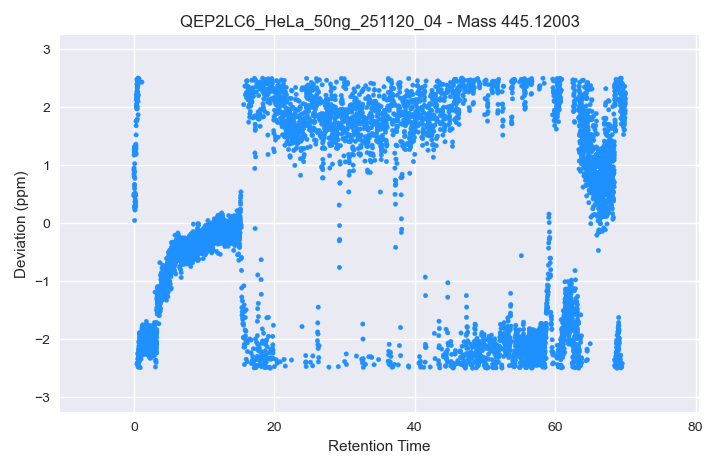

Supplement: Supplementary file 1 — pr0c00956_si_002.zip [file pr0c00956_si_002.zip › RawBeans_report/resources/images/QEP2LC6_HeLa_50ng_251120_04-mass-deviation2.png]

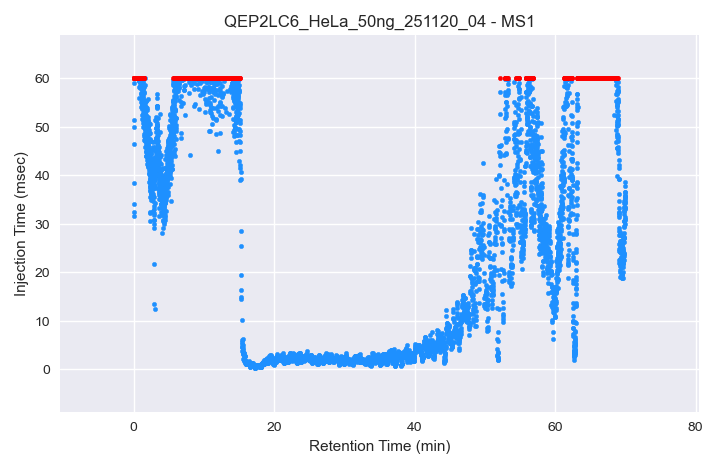

Supplement: Supplementary file 1 — pr0c00956_si_002.zip [file pr0c00956_si_002.zip › RawBeans_report/resources/images/QEP2LC6_HeLa_50ng_251120_04-ms1-inject-vs-ret.png]

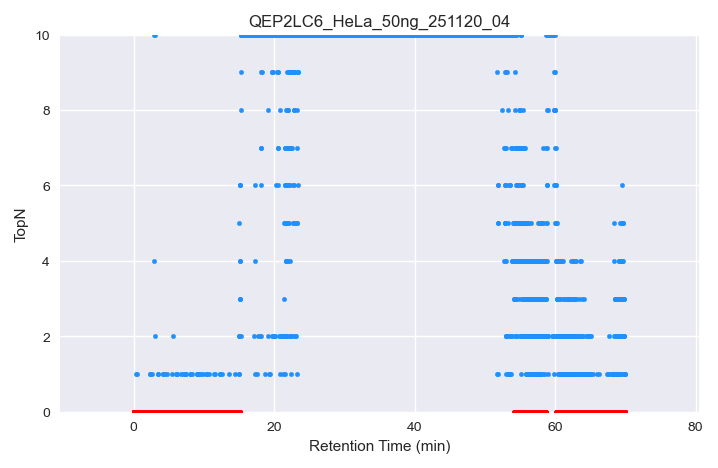

Supplement: Supplementary file 1 — pr0c00956_si_002.zip [file pr0c00956_si_002.zip › RawBeans_report/resources/images/QEP2LC6_HeLa_50ng_251120_04-ms1-ret-vs-top-n.png]

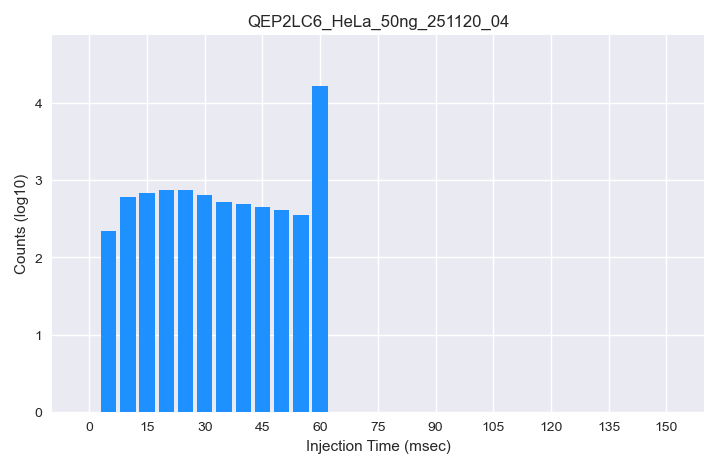

Supplement: Supplementary file 1 — pr0c00956_si_002.zip [file pr0c00956_si_002.zip › RawBeans_report/resources/images/QEP2LC6_HeLa_50ng_251120_04-ms2-inject.png]

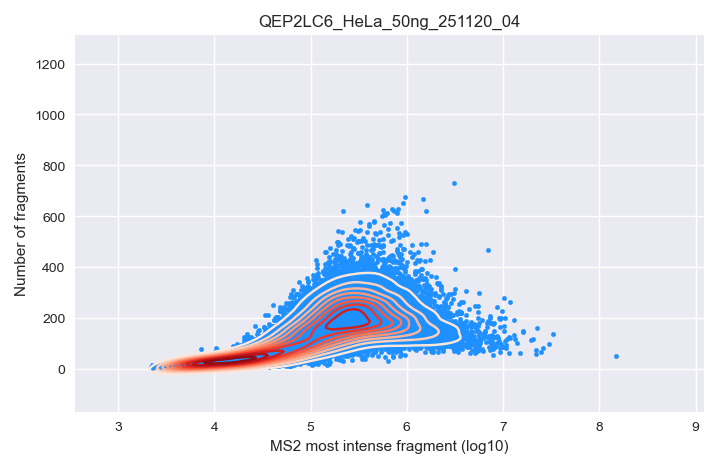

Supplement: Supplementary file 1 — pr0c00956_si_002.zip [file pr0c00956_si_002.zip › RawBeans_report/resources/images/QEP2LC6_HeLa_50ng_251120_04-ms2-max-log-intensity-vs-ms2-num-intensities.png]

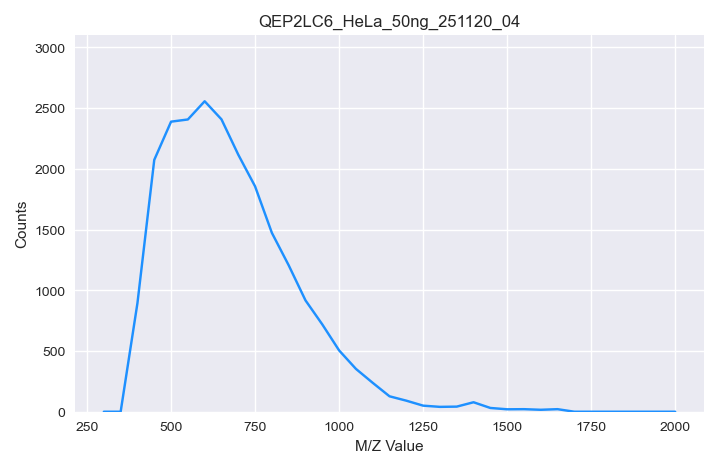

Supplement: Supplementary file 1 — pr0c00956_si_002.zip [file pr0c00956_si_002.zip › RawBeans_report/resources/images/QEP2LC6_HeLa_50ng_251120_04-ms2-mz-value.png]

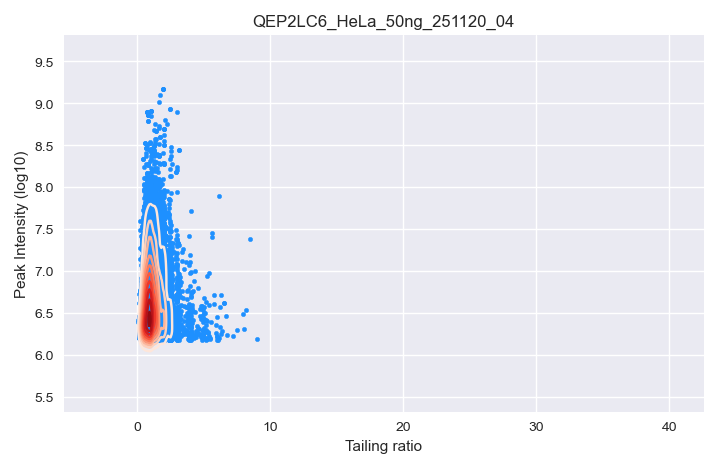

Supplement: Supplementary file 1 — pr0c00956_si_002.zip [file pr0c00956_si_002.zip › RawBeans_report/resources/images/QEP2LC6_HeLa_50ng_251120_04-peak-intentsity-vs-t2-t1-ratio.png]

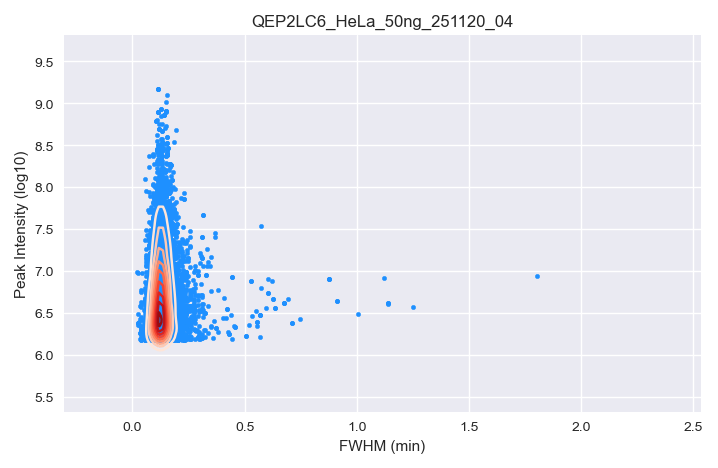

Supplement: Supplementary file 1 — pr0c00956_si_002.zip [file pr0c00956_si_002.zip › RawBeans_report/resources/images/QEP2LC6_HeLa_50ng_251120_04-peak-intentsity-vs-t-sum.png]

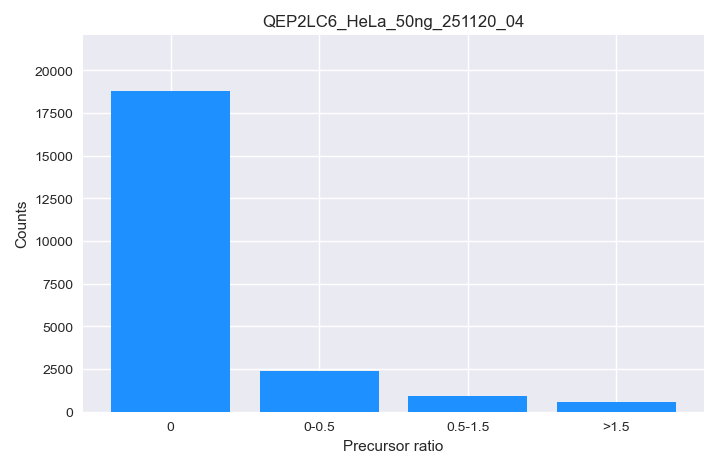

Supplement: Supplementary file 1 — pr0c00956_si_002.zip [file pr0c00956_si_002.zip › RawBeans_report/resources/images/QEP2LC6_HeLa_50ng_251120_04-prec-ratio.png]

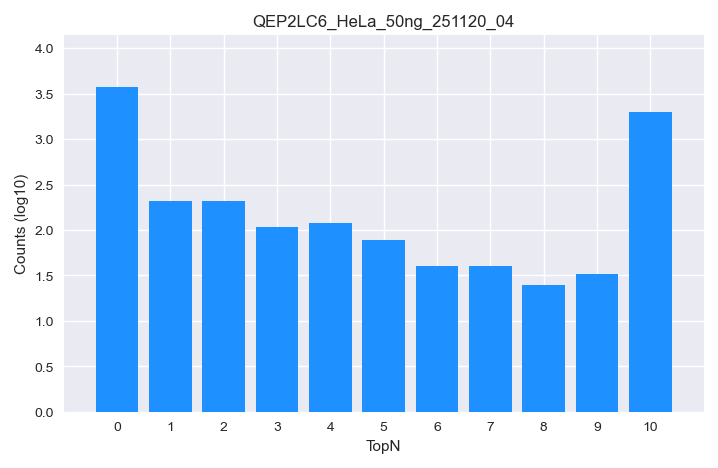

Supplement: Supplementary file 1 — pr0c00956_si_002.zip [file pr0c00956_si_002.zip › RawBeans_report/resources/images/QEP2LC6_HeLa_50ng_251120_04-top-n.png]

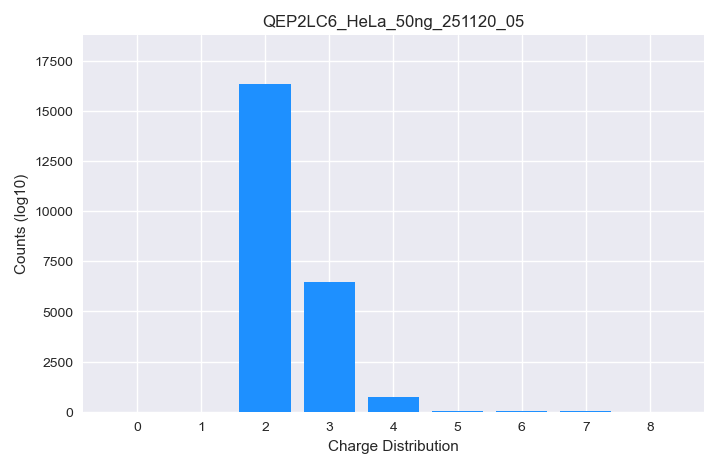

Supplement: Supplementary file 1 — pr0c00956_si_002.zip [file pr0c00956_si_002.zip › RawBeans_report/resources/images/QEP2LC6_HeLa_50ng_251120_05-charge-state.png]

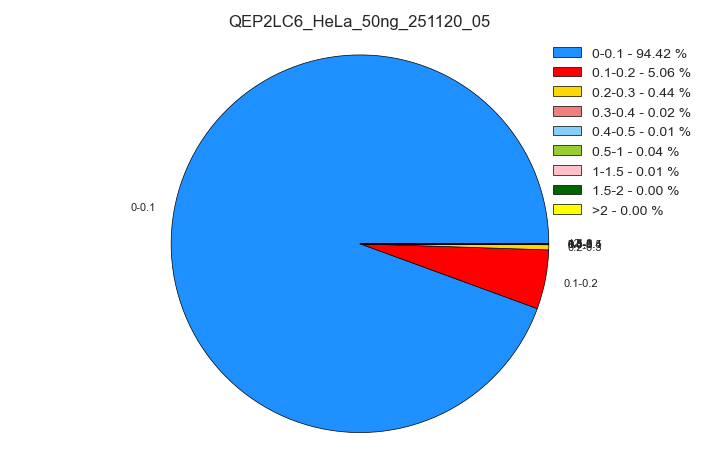

Supplement: Supplementary file 1 — pr0c00956_si_002.zip [file pr0c00956_si_002.zip › RawBeans_report/resources/images/QEP2LC6_HeLa_50ng_251120_05-fmhw-pie.png]

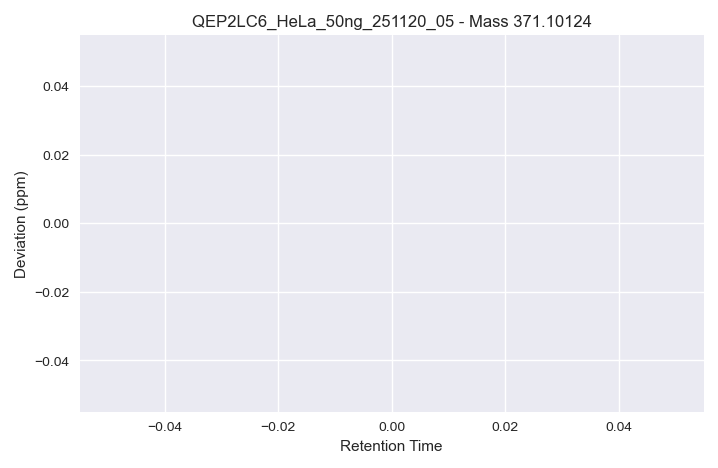

Supplement: Supplementary file 1 — pr0c00956_si_002.zip [file pr0c00956_si_002.zip › RawBeans_report/resources/images/QEP2LC6_HeLa_50ng_251120_05-mass-deviation1.png]

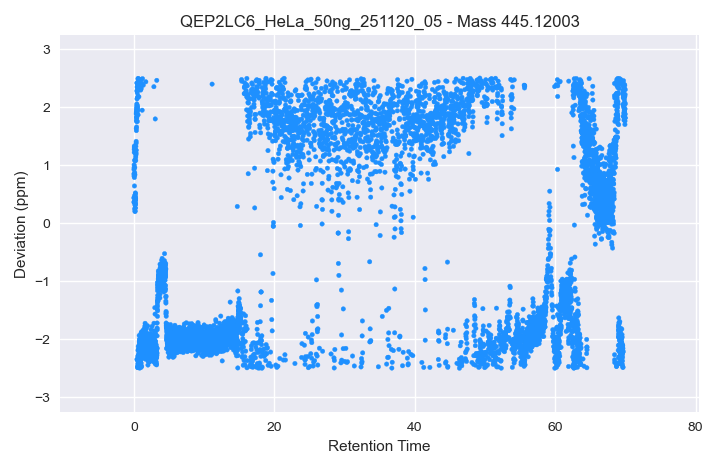

Supplement: Supplementary file 1 — pr0c00956_si_002.zip [file pr0c00956_si_002.zip › RawBeans_report/resources/images/QEP2LC6_HeLa_50ng_251120_05-mass-deviation2.png]

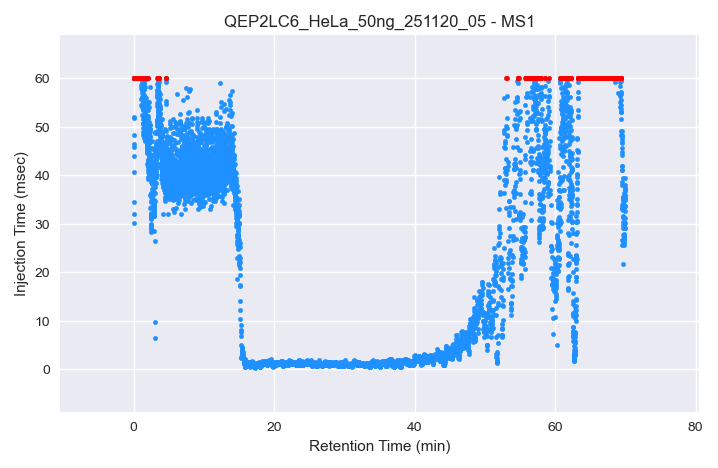

Supplement: Supplementary file 1 — pr0c00956_si_002.zip [file pr0c00956_si_002.zip › RawBeans_report/resources/images/QEP2LC6_HeLa_50ng_251120_05-ms1-inject-vs-ret.png]

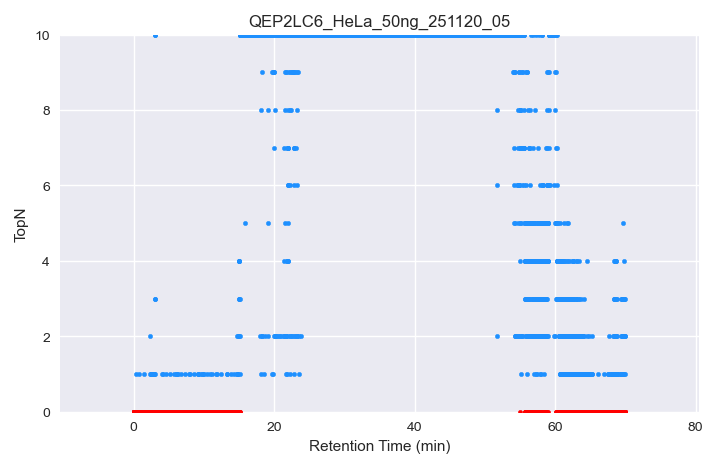

Supplement: Supplementary file 1 — pr0c00956_si_002.zip [file pr0c00956_si_002.zip › RawBeans_report/resources/images/QEP2LC6_HeLa_50ng_251120_05-ms1-ret-vs-top-n.png]

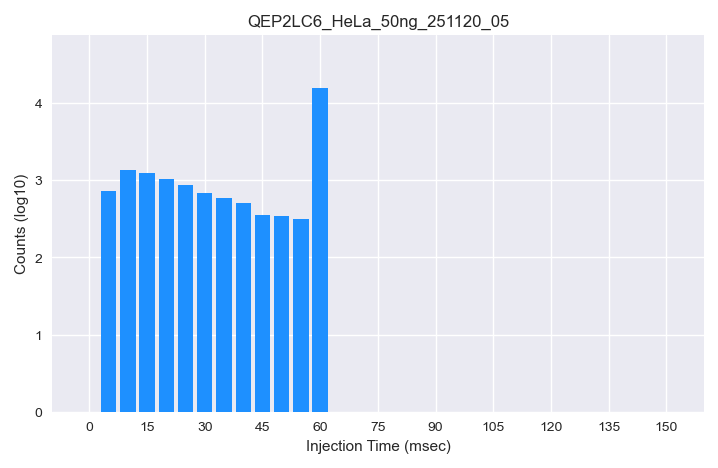

Supplement: Supplementary file 1 — pr0c00956_si_002.zip [file pr0c00956_si_002.zip › RawBeans_report/resources/images/QEP2LC6_HeLa_50ng_251120_05-ms2-inject.png]

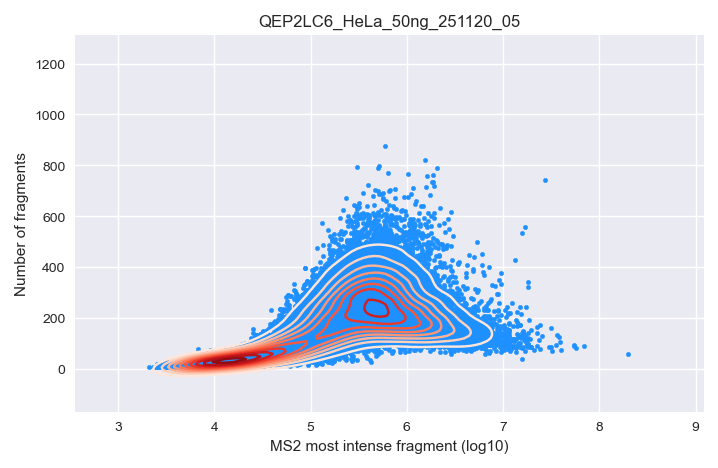

Supplement: Supplementary file 1 — pr0c00956_si_002.zip [file pr0c00956_si_002.zip › RawBeans_report/resources/images/QEP2LC6_HeLa_50ng_251120_05-ms2-max-log-intensity-vs-ms2-num-intensities.png]

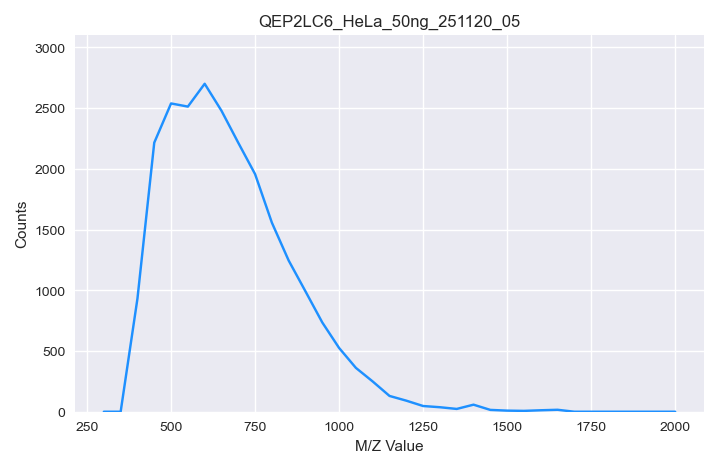

Supplement: Supplementary file 1 — pr0c00956_si_002.zip [file pr0c00956_si_002.zip › RawBeans_report/resources/images/QEP2LC6_HeLa_50ng_251120_05-ms2-mz-value.png]

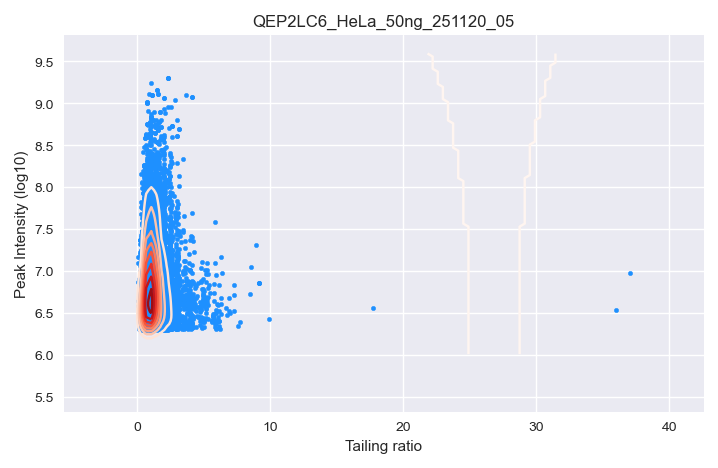

Supplement: Supplementary file 1 — pr0c00956_si_002.zip [file pr0c00956_si_002.zip › RawBeans_report/resources/images/QEP2LC6_HeLa_50ng_251120_05-peak-intentsity-vs-t2-t1-ratio.png]

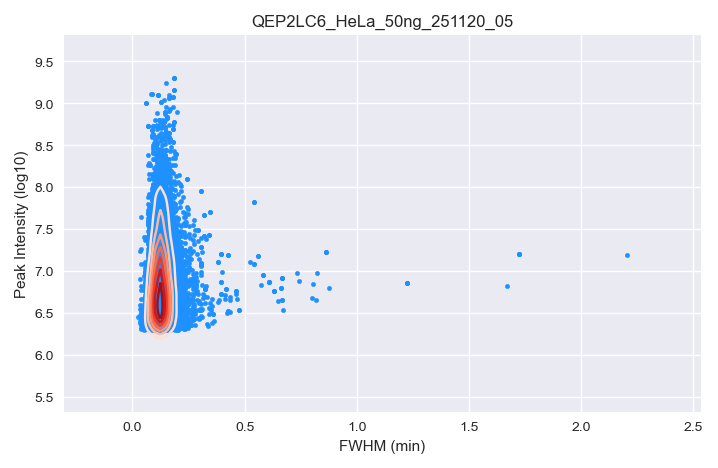

Supplement: Supplementary file 1 — pr0c00956_si_002.zip [file pr0c00956_si_002.zip › RawBeans_report/resources/images/QEP2LC6_HeLa_50ng_251120_05-peak-intentsity-vs-t-sum.png]

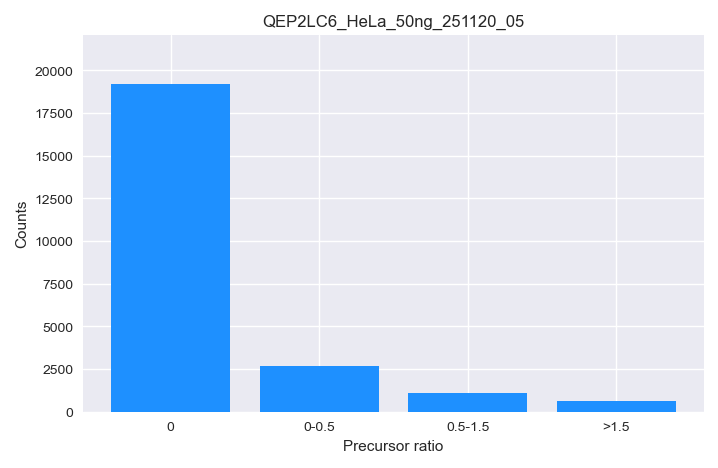

Supplement: Supplementary file 1 — pr0c00956_si_002.zip [file pr0c00956_si_002.zip › RawBeans_report/resources/images/QEP2LC6_HeLa_50ng_251120_05-prec-ratio.png]

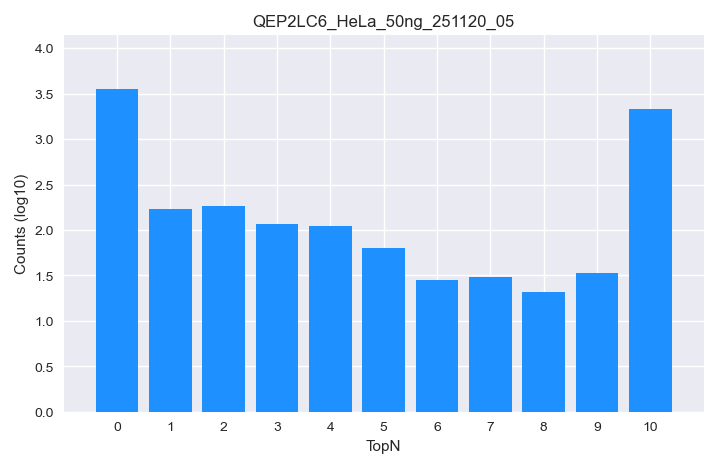

Supplement: Supplementary file 1 — pr0c00956_si_002.zip [file pr0c00956_si_002.zip › RawBeans_report/resources/images/QEP2LC6_HeLa_50ng_251120_05-top-n.png]

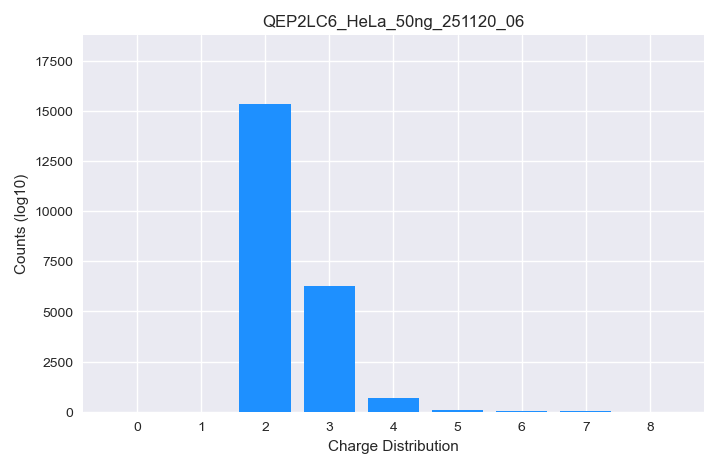

Supplement: Supplementary file 1 — pr0c00956_si_002.zip [file pr0c00956_si_002.zip › RawBeans_report/resources/images/QEP2LC6_HeLa_50ng_251120_06-charge-state.png]

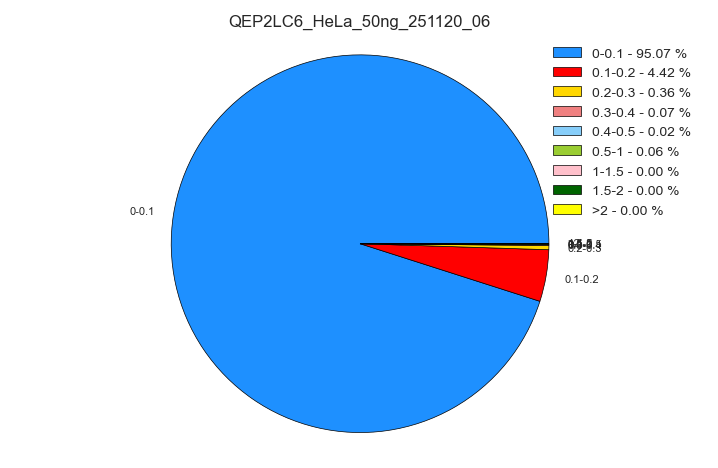

Supplement: Supplementary file 1 — pr0c00956_si_002.zip [file pr0c00956_si_002.zip › RawBeans_report/resources/images/QEP2LC6_HeLa_50ng_251120_06-fmhw-pie.png]

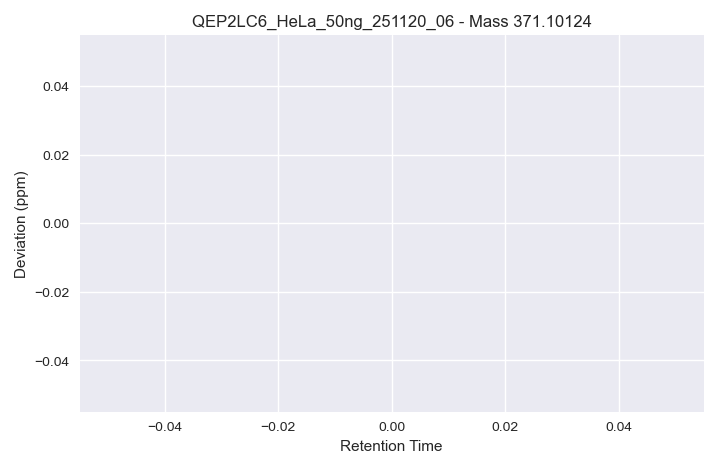

Supplement: Supplementary file 1 — pr0c00956_si_002.zip [file pr0c00956_si_002.zip › RawBeans_report/resources/images/QEP2LC6_HeLa_50ng_251120_06-mass-deviation1.png]

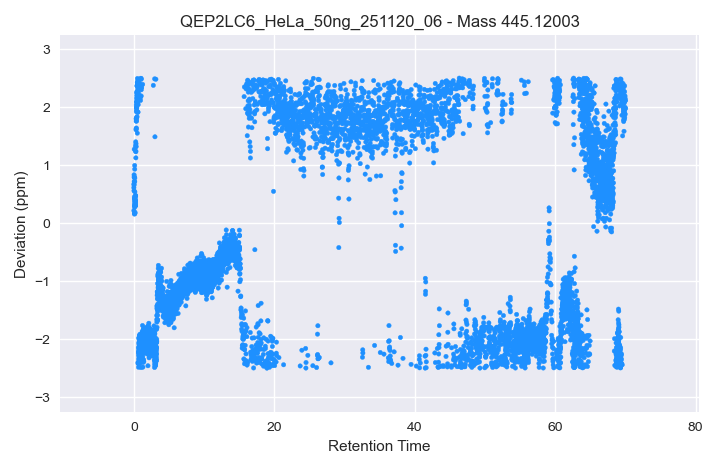

Supplement: Supplementary file 1 — pr0c00956_si_002.zip [file pr0c00956_si_002.zip › RawBeans_report/resources/images/QEP2LC6_HeLa_50ng_251120_06-mass-deviation2.png]

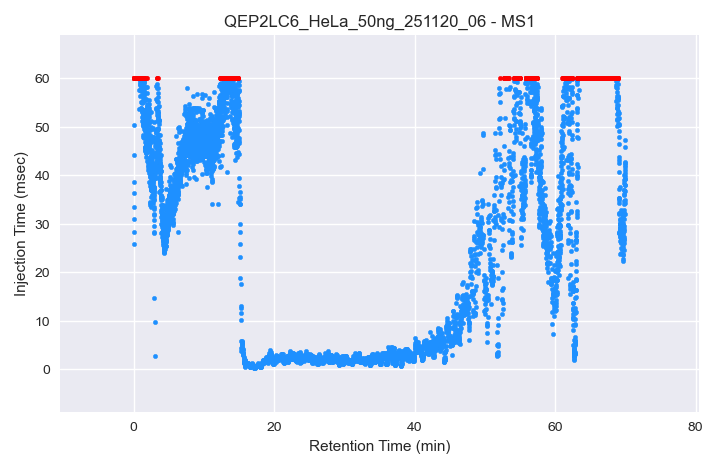

Supplement: Supplementary file 1 — pr0c00956_si_002.zip [file pr0c00956_si_002.zip › RawBeans_report/resources/images/QEP2LC6_HeLa_50ng_251120_06-ms1-inject-vs-ret.png]

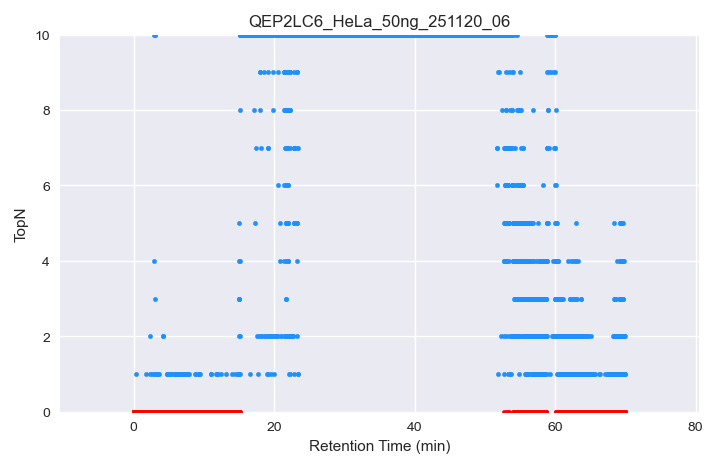

Supplement: Supplementary file 1 — pr0c00956_si_002.zip [file pr0c00956_si_002.zip › RawBeans_report/resources/images/QEP2LC6_HeLa_50ng_251120_06-ms1-ret-vs-top-n.png]

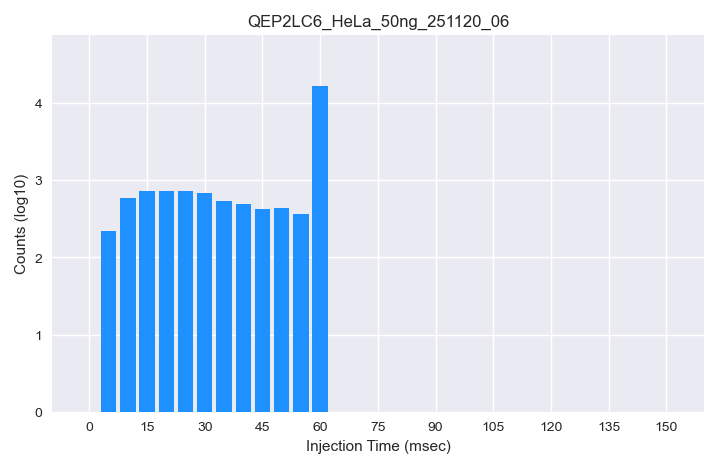

Supplement: Supplementary file 1 — pr0c00956_si_002.zip [file pr0c00956_si_002.zip › RawBeans_report/resources/images/QEP2LC6_HeLa_50ng_251120_06-ms2-inject.png]

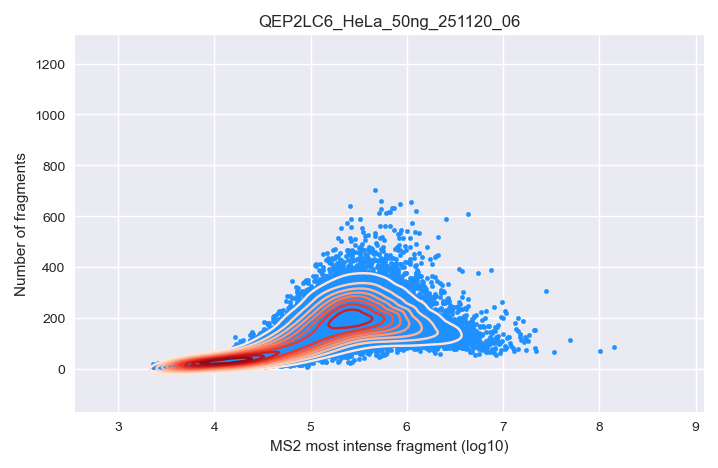

Supplement: Supplementary file 1 — pr0c00956_si_002.zip [file pr0c00956_si_002.zip › RawBeans_report/resources/images/QEP2LC6_HeLa_50ng_251120_06-ms2-max-log-intensity-vs-ms2-num-intensities.png]

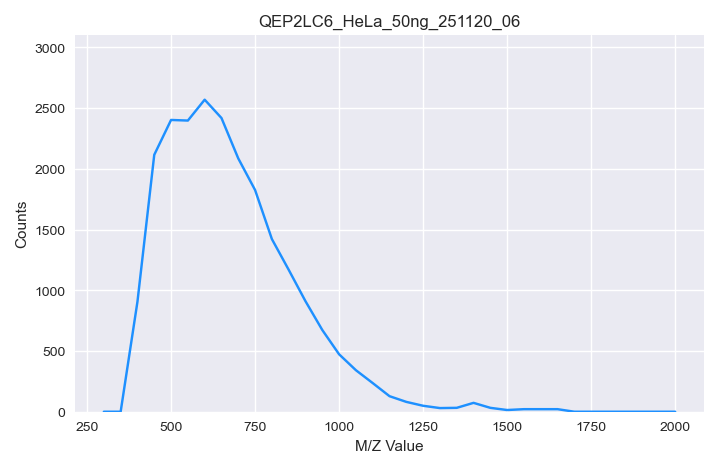

Supplement: Supplementary file 1 — pr0c00956_si_002.zip [file pr0c00956_si_002.zip › RawBeans_report/resources/images/QEP2LC6_HeLa_50ng_251120_06-ms2-mz-value.png]

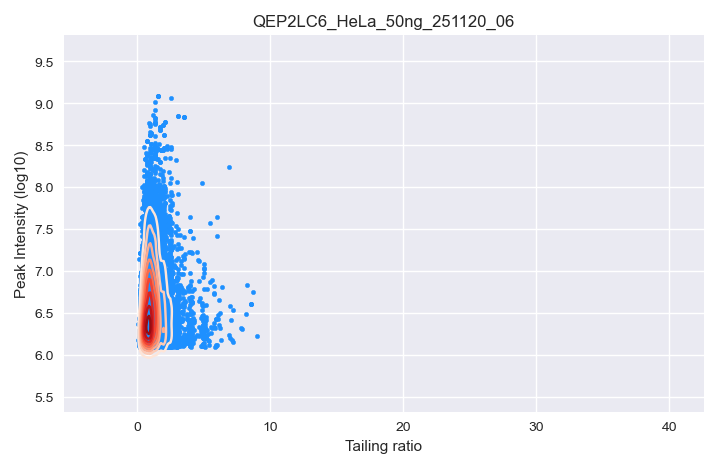

Supplement: Supplementary file 1 — pr0c00956_si_002.zip [file pr0c00956_si_002.zip › RawBeans_report/resources/images/QEP2LC6_HeLa_50ng_251120_06-peak-intentsity-vs-t2-t1-ratio.png]

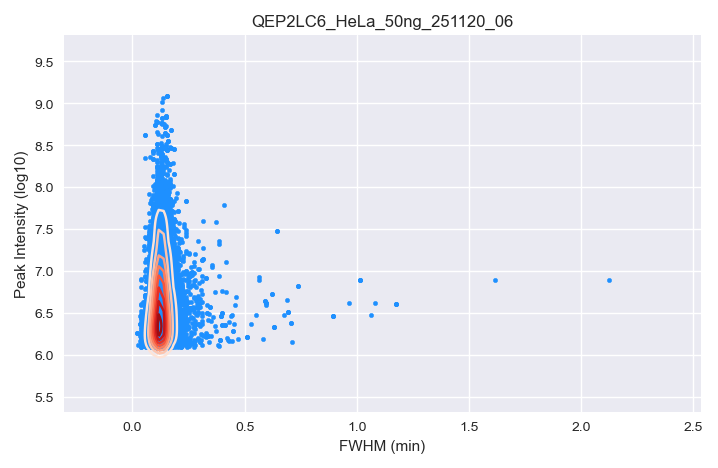

Supplement: Supplementary file 1 — pr0c00956_si_002.zip [file pr0c00956_si_002.zip › RawBeans_report/resources/images/QEP2LC6_HeLa_50ng_251120_06-peak-intentsity-vs-t-sum.png]

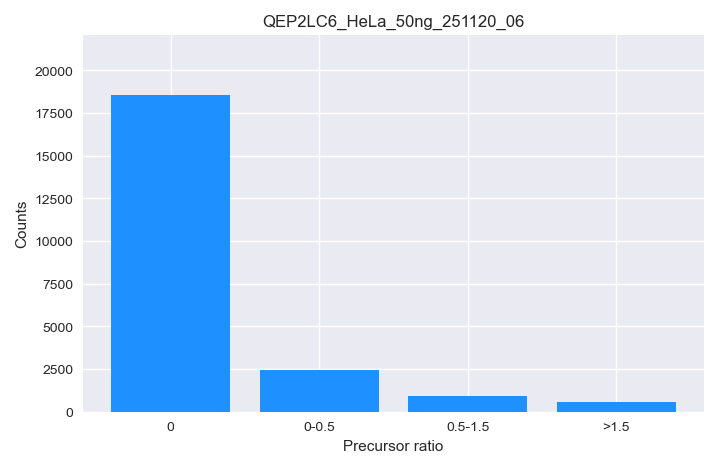

Supplement: Supplementary file 1 — pr0c00956_si_002.zip [file pr0c00956_si_002.zip › RawBeans_report/resources/images/QEP2LC6_HeLa_50ng_251120_06-prec-ratio.png]

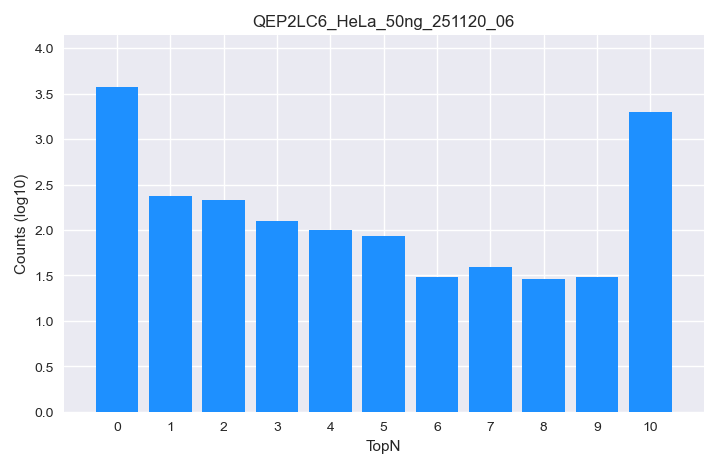

Supplement: Supplementary file 1 — pr0c00956_si_002.zip [file pr0c00956_si_002.zip › RawBeans_report/resources/images/QEP2LC6_HeLa_50ng_251120_06-top-n.png]

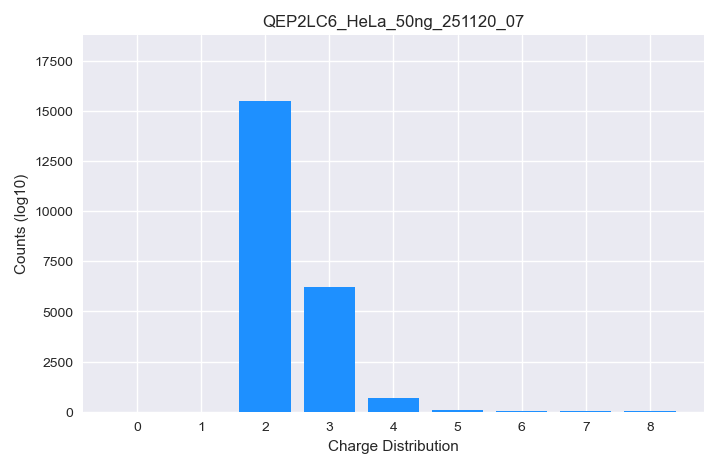

Supplement: Supplementary file 1 — pr0c00956_si_002.zip [file pr0c00956_si_002.zip › RawBeans_report/resources/images/QEP2LC6_HeLa_50ng_251120_07-charge-state.png]

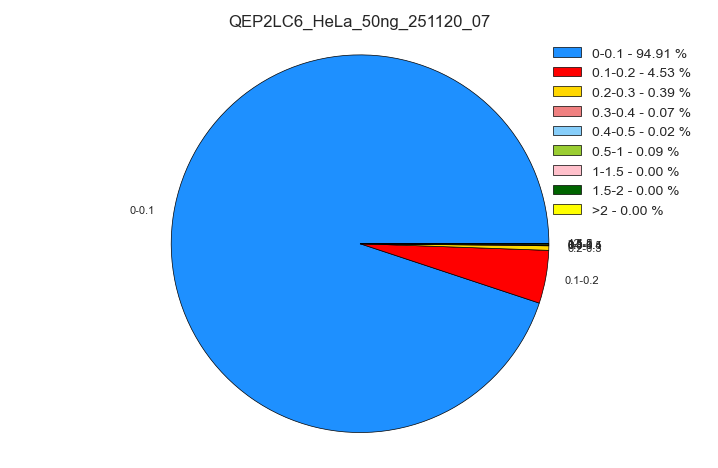

Supplement: Supplementary file 1 — pr0c00956_si_002.zip [file pr0c00956_si_002.zip › RawBeans_report/resources/images/QEP2LC6_HeLa_50ng_251120_07-fmhw-pie.png]

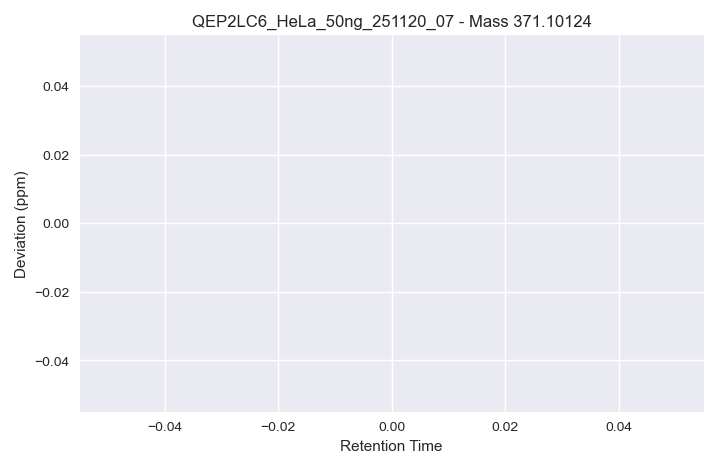

Supplement: Supplementary file 1 — pr0c00956_si_002.zip [file pr0c00956_si_002.zip › RawBeans_report/resources/images/QEP2LC6_HeLa_50ng_251120_07-mass-deviation1.png]

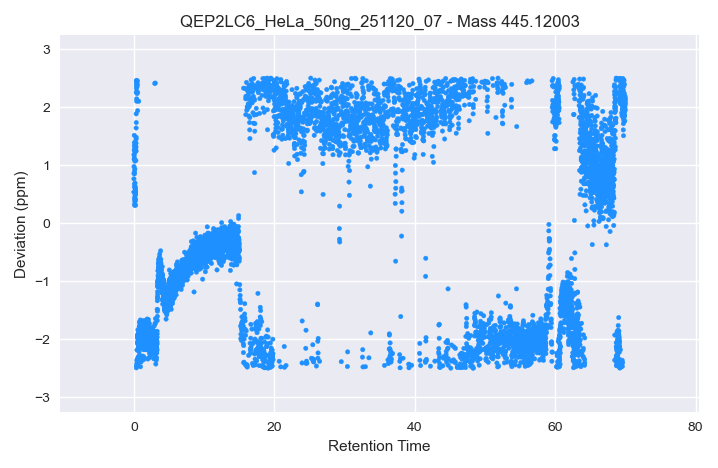

Supplement: Supplementary file 1 — pr0c00956_si_002.zip [file pr0c00956_si_002.zip › RawBeans_report/resources/images/QEP2LC6_HeLa_50ng_251120_07-mass-deviation2.png]

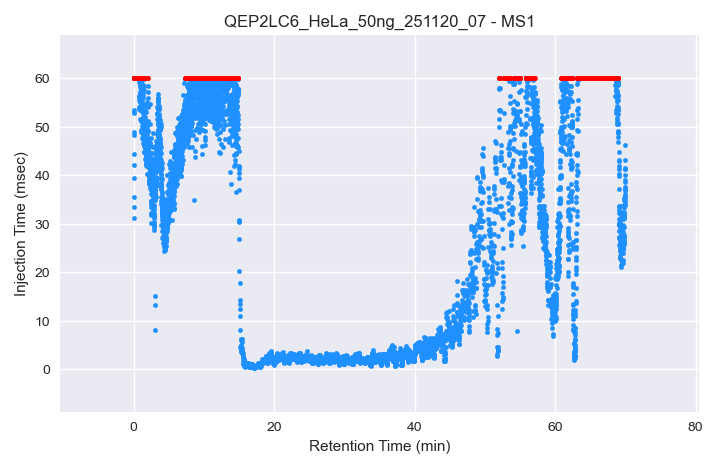

Supplement: Supplementary file 1 — pr0c00956_si_002.zip [file pr0c00956_si_002.zip › RawBeans_report/resources/images/QEP2LC6_HeLa_50ng_251120_07-ms1-inject-vs-ret.png]

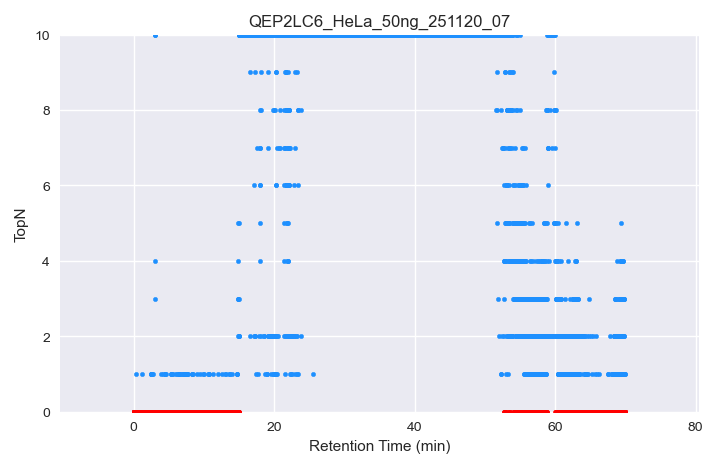

Supplement: Supplementary file 1 — pr0c00956_si_002.zip [file pr0c00956_si_002.zip › RawBeans_report/resources/images/QEP2LC6_HeLa_50ng_251120_07-ms1-ret-vs-top-n.png]

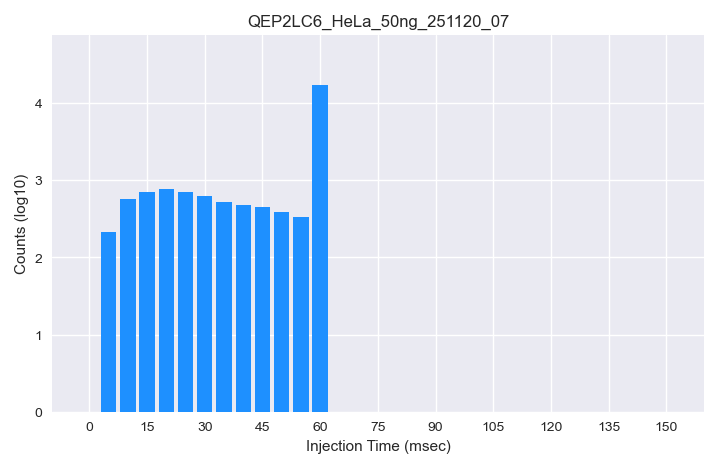

Supplement: Supplementary file 1 — pr0c00956_si_002.zip [file pr0c00956_si_002.zip › RawBeans_report/resources/images/QEP2LC6_HeLa_50ng_251120_07-ms2-inject.png]

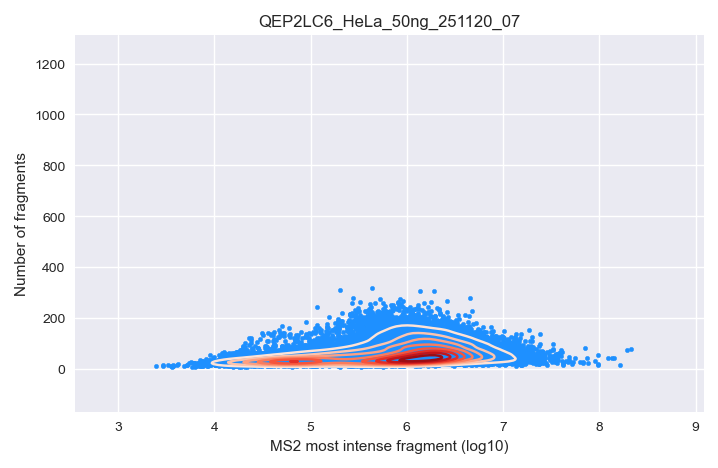

Supplement: Supplementary file 1 — pr0c00956_si_002.zip [file pr0c00956_si_002.zip › RawBeans_report/resources/images/QEP2LC6_HeLa_50ng_251120_07-ms2-max-log-intensity-vs-ms2-num-intensities.png]

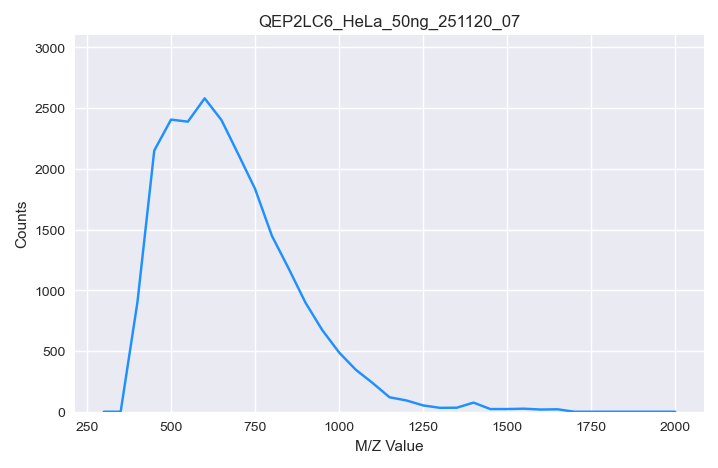

Supplement: Supplementary file 1 — pr0c00956_si_002.zip [file pr0c00956_si_002.zip › RawBeans_report/resources/images/QEP2LC6_HeLa_50ng_251120_07-ms2-mz-value.png]

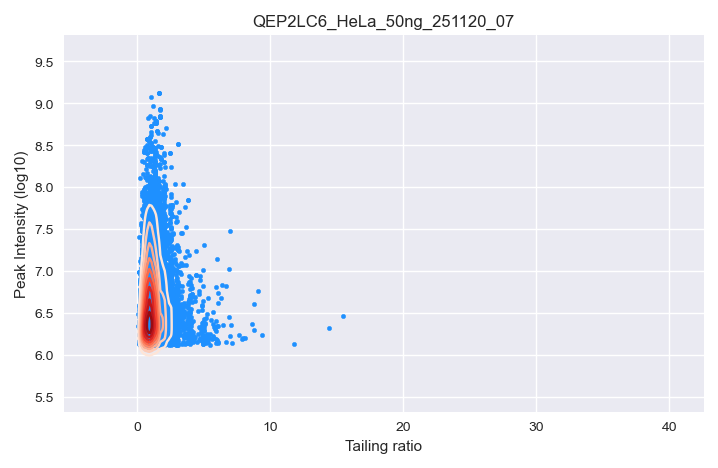

Supplement: Supplementary file 1 — pr0c00956_si_002.zip [file pr0c00956_si_002.zip › RawBeans_report/resources/images/QEP2LC6_HeLa_50ng_251120_07-peak-intentsity-vs-t2-t1-ratio.png]
