# Supplementary material for: RawBeans: A Simple, Vendor-Independent, Raw-Data Quality-Control Tool
Source: J Proteome Res. 2021 Mar 4;20(4):2098–104. doi: 10.1021/acs.jproteome.0c00956 (PMC8041395; doi:10.1021/acs.jproteome.0c00956)
Supplement: Supplementary file 2 — pr0c00956_si_003.zip [file pr0c00956_si_003.zip › 100_samples_run1.0/qc-report.html]

QC Report


# Raw Beans

MS2 Counts
Top-N
Charge Distribution
Injection Time
Retention vs. Top-N
Injection vs. Retention
Total Ion Current
MS2-Intensities
MS2 Precursor Ratio
Triggered M/Z Distribution
FWHM
Peak Symmetry
Mass Deviation
Help

## Number of MS/MS Triggered per File

| Sample Name | # MS/MS Triggered | Peak Split? |
| --- | --- | --- |
| HF2\_LC1\_hela\_120120\_01\_35B | 28248 | False |
| HF2\_LC1\_hela\_111020\_01 | 29803 | False |
| HF2\_LC1\_hela\_150520\_01 | 31288 | False |
| HF2\_LC1\_hela\_030620\_01 | 468 | False |
| HF2\_LC1\_hela\_090820\_01 | 31958 | False |
| HF2\_LC1\_hela\_170320\_30B\_03 | 31445 | False |
| HF2\_LC1\_hela\_150120\_01\_30B | 30933 | False |
| HF2\_LC1\_hela\_070120\_04\_25B | 1110 | False |
| HF2\_LC1\_hela\_220720\_02 | 30650 | False |
| HF2\_LC1\_hela\_120320\_30B\_01 | 32148 | False |
| HF2\_LC1\_hela\_270520\_01 | 31671 | False |
| HF2\_LC1\_hela\_110820\_02 | 31202 | False |
| HF2\_LC1\_hela\_291020\_01 | 30045 | False |
| HF2\_LC1\_hela\_210520\_01 | 31532 | False |
| HF2\_LC1\_hela\_010720\_01 | 34576 | False |
| HF2\_LC1\_hela\_050520\_01 | 32123 | False |
| HF2\_LC1\_hela\_020320\_30B\_01 | 32199 | False |
| HF2\_LC1\_hela\_180520\_03 | 31949 | False |
| HF2\_LC1\_hela\_220620\_01 | 33554 | False |
| HF2\_LC1\_hela\_270720\_01 | 31567 | False |
| HF2\_LC1\_hela\_221020\_01 | 30285 | False |
| HF2\_LC1\_hela\_020520\_01 | 32572 | False |
| HF2\_LC1\_hela\_300720\_01 | 30891 | False |
| HF2\_LC1\_hela\_300420\_30B\_01 | 31022 | False |
| HF2\_LC1\_hela\_020820\_01 | 32501 | False |
| HF2\_LC1\_hela\_090120\_01\_30B | 30007 | False |
| HF2\_LC1\_hela\_261120\_01 | 29779 | False |
| HF2\_LC1\_hela\_020420\_30B\_01 | 29873 | False |
| HF2\_LC1\_hela\_021120\_01 | 30348 | False |
| HF2\_LC1\_hela\_190420\_30B\_01 | 31203 | False |
| HF2\_LC1\_hela\_170320\_30B\_01 | 31474 | False |
| HF2\_LC1\_hela\_100120\_01\_30B | 29318 | False |
| HF2\_LC1\_hela\_260620\_01 | 36300 | False |
| HF2\_LC1\_hela\_130420\_30B\_01 | 31090 | False |
| HF2\_LC1\_hela\_080720\_02 | 32452 | False |
| HF2\_LC1\_hela\_230820\_02 | 32662 | False |
| HF2\_LC1\_hela\_211020\_01 | 30003 | False |
| HF2\_LC1\_hela\_190620\_01 | 32549 | False |
| HF2\_LC1\_hela\_060320\_30B\_01 | 32721 | False |
| HF2\_LC1\_hela\_110820\_01 | 1440 | False |
| HF2\_LC1\_hela\_260320\_30B\_01 | 31123 | False |
| HF2\_LC1\_hela\_100720\_01 | 32107 | False |
| HF2\_LC1\_hela\_030220\_30B\_01 | 32554 | False |
| HF2\_LC1\_hela\_290320\_30B\_01 | 30751 | False |
| HF2\_LC1\_hela\_120520\_01 | 31105 | False |
| HF2\_LC1\_hela\_060220\_30B\_01 | 32469 | False |
| HF2\_LC1\_hela\_180520\_New\_batch | 31845 | False |
| HF2\_LC1\_hela\_180520\_01 | 29576 | False |
| HF2\_LC1\_hela\_220720\_01 | 28814 | False |
| HF2\_LC1\_hela\_090620\_01 | 30956 | False |
| HF2\_LC1\_hela\_190420\_30B\_02 | 32433 | False |
| HF2\_LC1\_hela\_221120\_02 | 30335 | False |
| HF2\_LC1\_hela\_080720\_01 | 27520 | False |
| HF2\_LC1\_hela\_220320\_30B\_01 | 31817 | False |
| HF2\_LC1\_hela\_261120\_02 | 31263 | False |
| HF2\_LC1\_hela\_020220\_30B\_01 | 32474 | False |
| HF2\_LC1\_hela\_081120\_01 | 29231 | False |
| HF2\_LC1\_hela\_030720\_03\_30B | 58131 | False |
| HF2\_LC1\_hela\_221120\_01 | 30582 | False |
| HF2\_LC1\_hela\_280620\_01 | 32717 | False |
| HF2\_LC1\_hela\_070120\_03\_30B | 29258 | False |
| HF2\_LC1\_hela\_291120\_01 | 30299 | False |
| HF2\_LC1\_hela\_290520\_01 | 30013 | False |
| HF2\_LC1\_hela\_070120\_02 | 29738 | False |
| HF2\_LC1\_hela\_170320\_30B\_02 | 31273 | False |
| HF2\_LC1\_hela\_260520\_01 | 31390 | False |
| HF2\_LC1\_hela\_251020\_01 | 30024 | False |
| HF2\_LC1\_hela\_130120\_02\_30B | 31227 | False |
| HF2\_LC1\_hela\_100320\_30B\_01 | 32416 | False |
| HF2\_LC1\_hela\_010120\_01 | 28142 | False |
| HF2\_LC1\_hela\_120120\_01\_30B | 29912 | False |
| HF2\_LC1\_hela\_160820\_01 | 32455 | False |
| HF2\_LC1\_hela\_041020\_01\_201004131621 | 29301 | False |
| HF2\_LC1\_hela\_190720\_01 | 33298 | False |
| HF2\_LC1\_hela\_280220\_30B\_01 | 32432 | False |
| HF2\_LC1\_hela\_070120\_01 | 28309 | False |
| HF2\_LC1\_hela\_050120\_01 | 29944 | False |
| HF2\_LC1\_hela\_180520\_02 | 31682 | False |
| HF2\_LC1\_hela\_181020\_01 | 28349 | False |
| HF2\_LC1\_hela\_030620\_01\_200604000800 | 32103 | False |
| HF2\_LC1\_hela\_090220\_30B\_01 | 32780 | False |
| HF2\_LC1\_hela\_200120\_01\_30B | 31673 | False |
| HF2\_LC1\_hela\_140720\_01 | 32274 | False |
| HF2\_LC1\_hela\_181020\_02 | 29009 | False |
| HF2\_LC1\_hela\_230820\_01 | 30300 | False |
| HF2\_LC1\_hela\_290120\_01\_30B | 32513 | False |

## TopN per Cycle

## Charge Distributions

## Injection Time

## Retention Time vs TopN

## Injection Time vs Retention Time

## Total Ion Current

Sort by Name

## MS2-Intensities

## MS2 Precursor Ratio

## Triggered M/Z Distribution

## FMHW

## Peak Symmetry

## Mass Deviation
