# Supplementary material for: RawBeans: A Simple, Vendor-Independent, Raw-Data Quality-Control Tool
Source: J Proteome Res. 2021 Mar 4;20(4):2098–104. doi: 10.1021/acs.jproteome.0c00956 (PMC8041395; doi:10.1021/acs.jproteome.0c00956)
Supplement: Supplementary file 3 — pr0c00956_si_004.zip [file pr0c00956_si_004.zip › ABSciex_data/qc-report.html]

QC Report


# Raw Beans

MS2 Counts
Top-N
Charge Distribution
Injection Time
Retention vs. Top-N
Injection vs. Retention
Total Ion Current
MS2-Intensities
MS2 Precursor Ratio
Triggered M/Z Distribution
FWHM
Peak Symmetry
Mass Deviation
Help

## Number of MS/MS Triggered per File

| Sample Name | # MS/MS Triggered | Peak Split? |
| --- | --- | --- |
| MK 20191021 6600 IDA C 1 1 | 15594 | False |
| MK 20191021 6600 IDA C 1 2 | 16781 | False |

## TopN per Cycle

## Charge Distributions

## Injection Time

## Retention Time vs TopN

## Injection Time vs Retention Time

## Total Ion Current

Sort by Name

## MS2-Intensities

## MS2 Precursor Ratio

## Triggered M/Z Distribution

## FMHW

## Peak Symmetry

## Mass Deviation
