# Supplementary figures and images for: RawBeans: A Simple, Vendor-Independent, Raw-Data Quality-Control Tool (part 3 of 3)
Source: J Proteome Res. 2021 Mar 4;20(4):2098–104. doi: 10.1021/acs.jproteome.0c00956 (PMC8041395; doi:10.1021/acs.jproteome.0c00956)

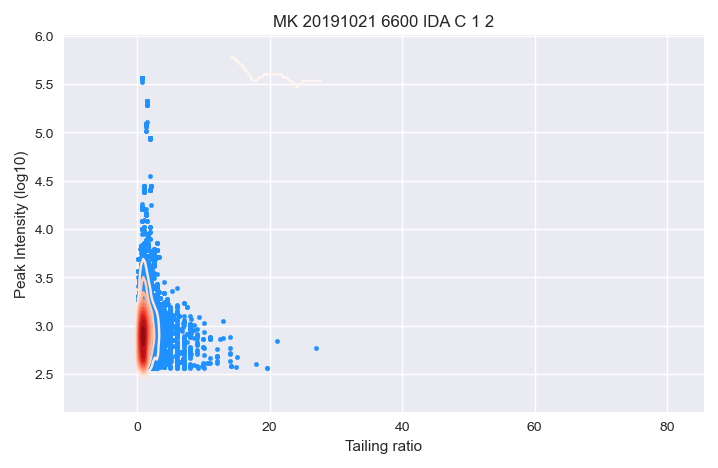

Supplement: Supplementary file 3 — pr0c00956_si_004.zip [file pr0c00956_si_004.zip › ABSciex_data/resources/images/MK 20191021 6600 IDA C 1 2-peak-intentsity-vs-t2-t1-ratio.png]

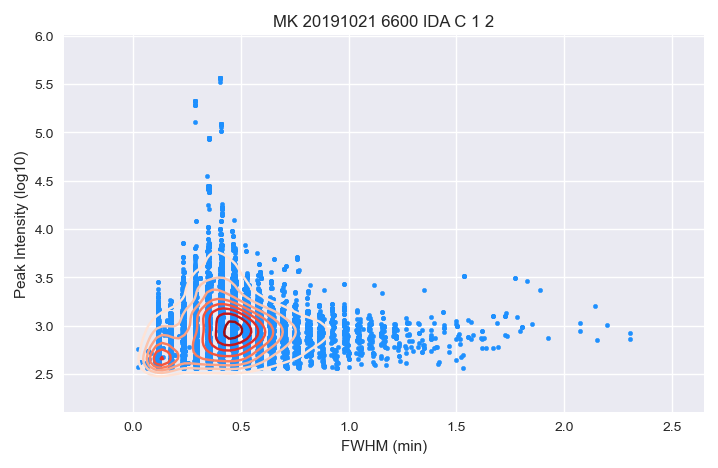

Supplement: Supplementary file 3 — pr0c00956_si_004.zip [file pr0c00956_si_004.zip › ABSciex_data/resources/images/MK 20191021 6600 IDA C 1 2-peak-intentsity-vs-t-sum.png]

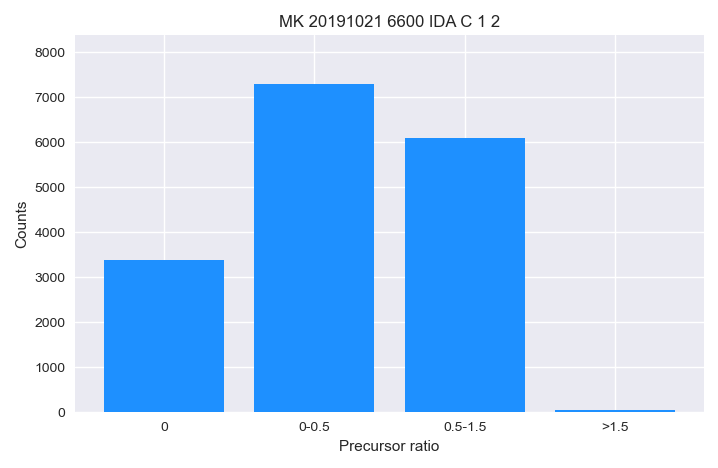

Supplement: Supplementary file 3 — pr0c00956_si_004.zip [file pr0c00956_si_004.zip › ABSciex_data/resources/images/MK 20191021 6600 IDA C 1 2-prec-ratio.png]

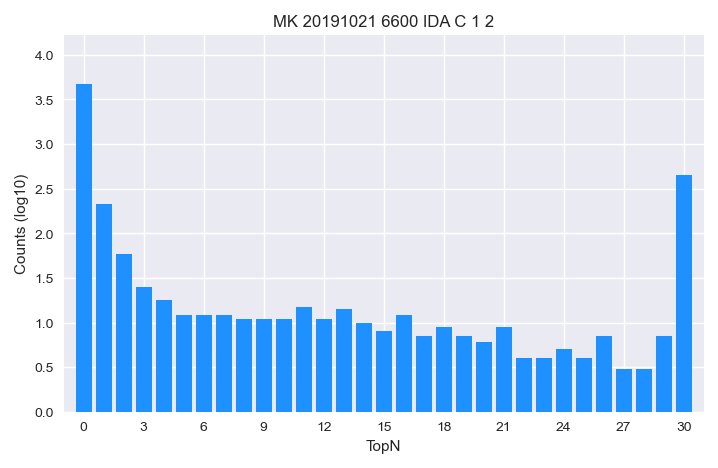

Supplement: Supplementary file 3 — pr0c00956_si_004.zip [file pr0c00956_si_004.zip › ABSciex_data/resources/images/MK 20191021 6600 IDA C 1 2-top-n.png]

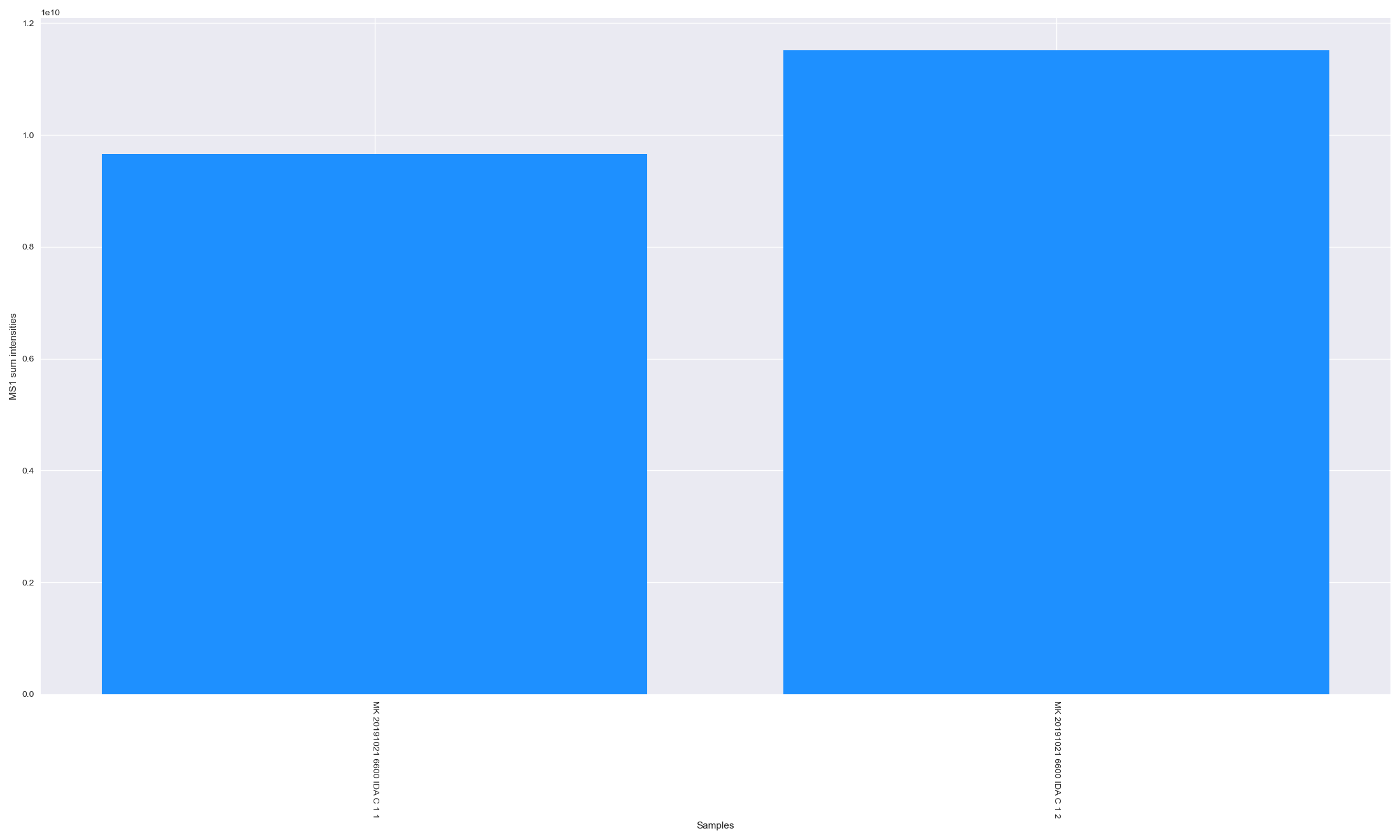

Supplement: Supplementary file 3 — pr0c00956_si_004.zip [file pr0c00956_si_004.zip › ABSciex_data/resources/images/tic-lex-sort.png]

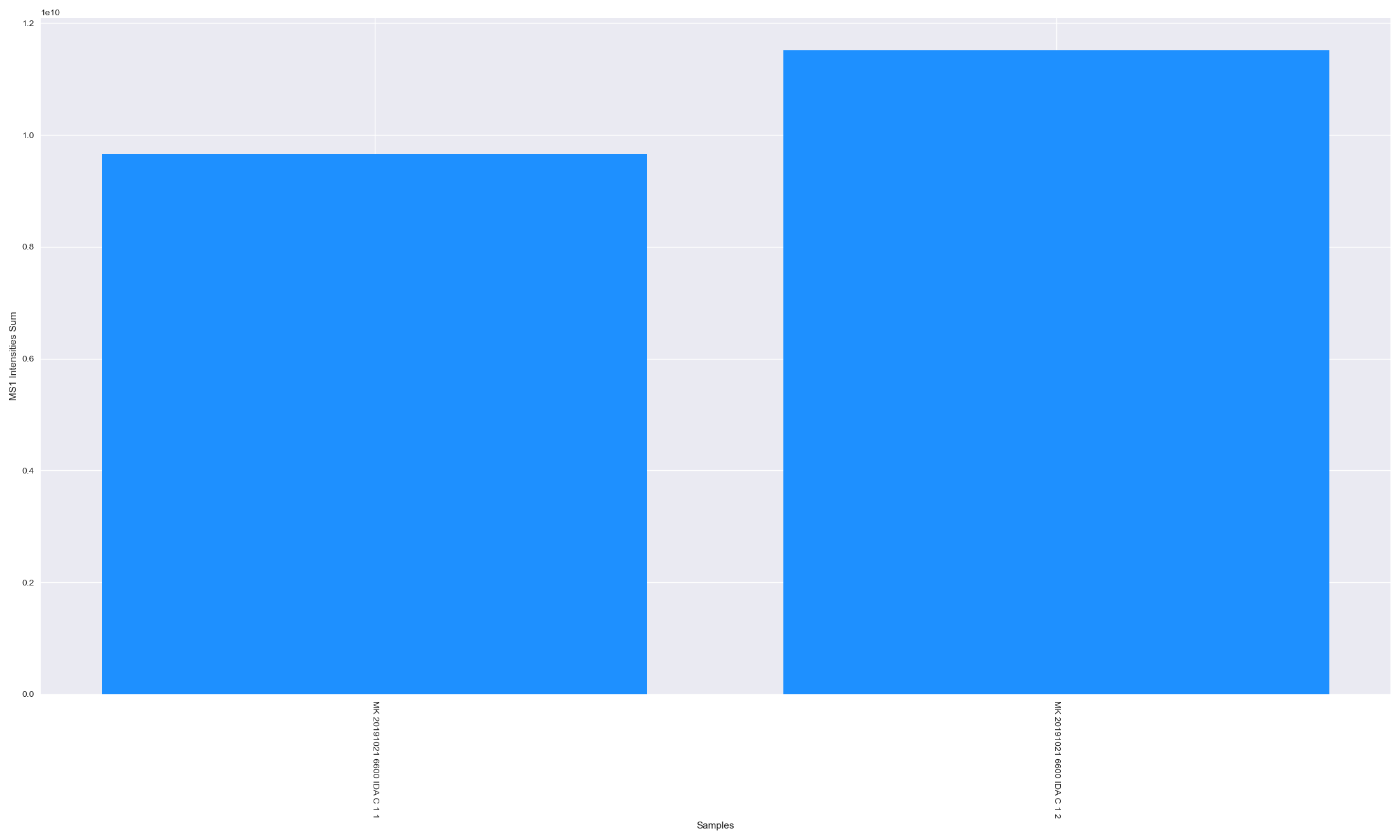

Supplement: Supplementary file 3 — pr0c00956_si_004.zip [file pr0c00956_si_004.zip › ABSciex_data/resources/images/tic-run-date-sort.png]

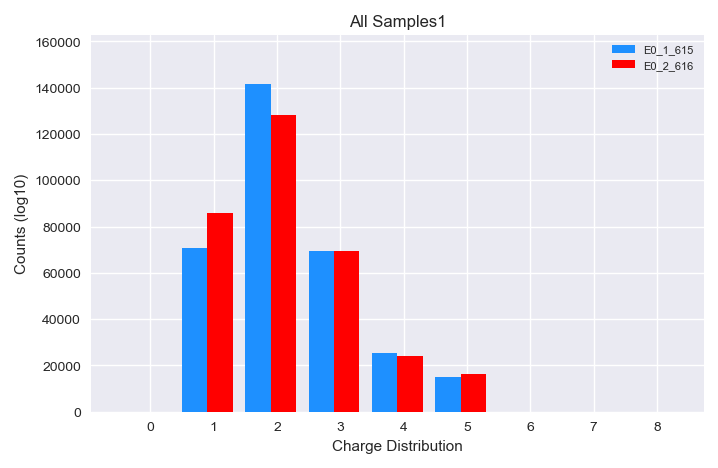

Supplement: Supplementary file 4 — pr0c00956_si_005.zip [file pr0c00956_si_005.zip › timstof_data/resources/images/all-samples1-charge-state.png]

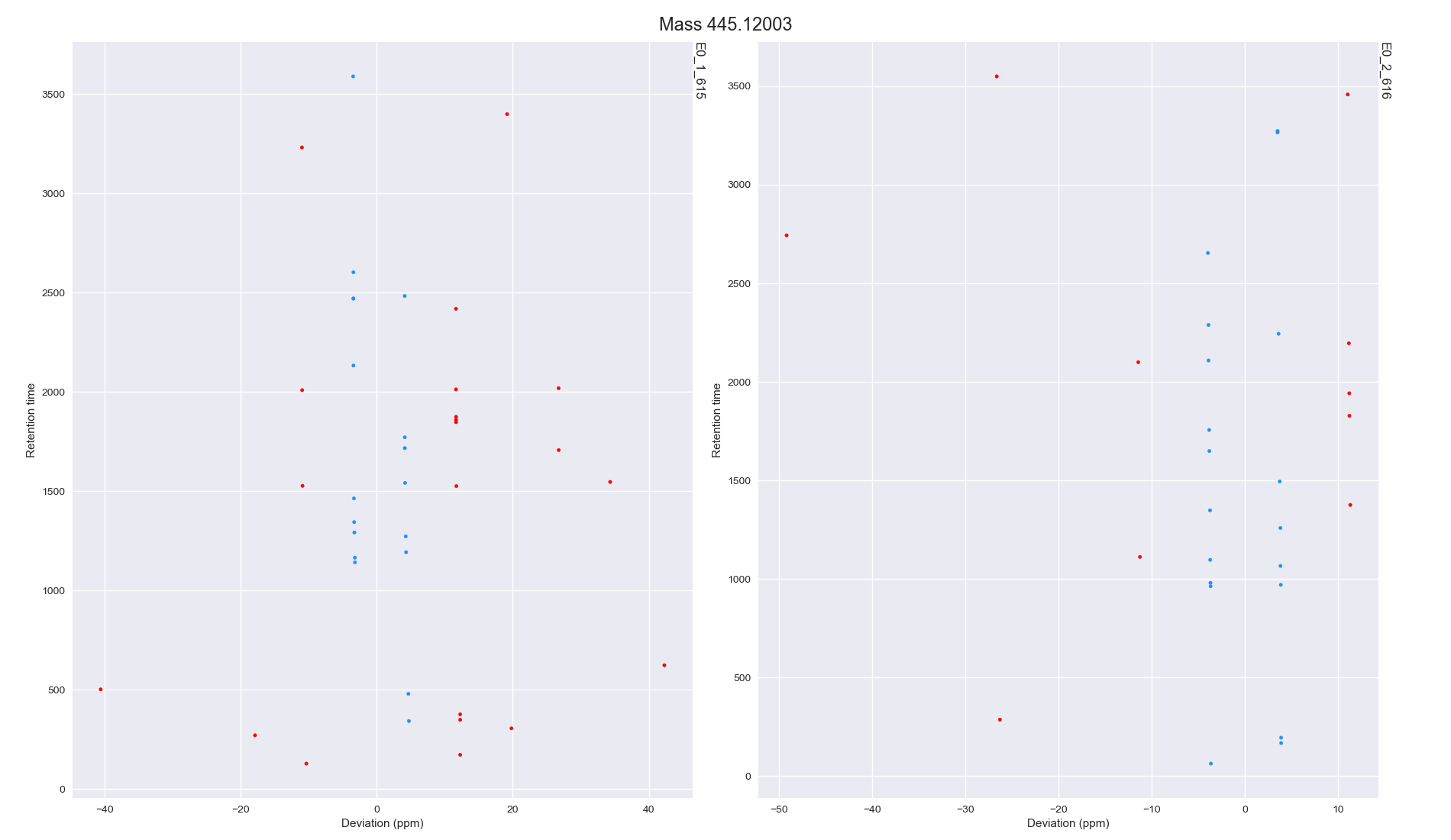

Supplement: Supplementary file 4 — pr0c00956_si_005.zip [file pr0c00956_si_005.zip › timstof_data/resources/images/all-samples1-mass1-deviation.png]

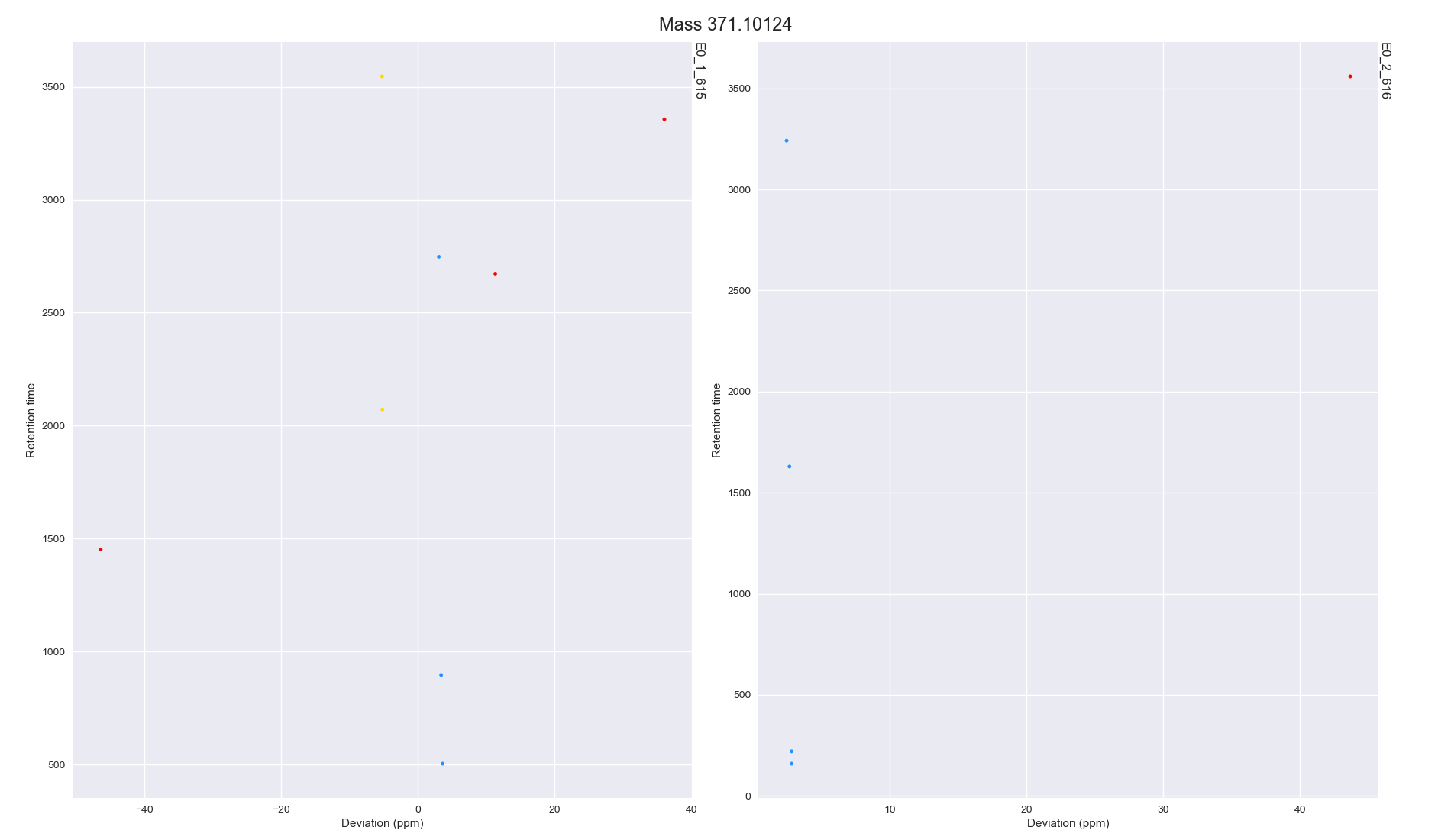

Supplement: Supplementary file 4 — pr0c00956_si_005.zip [file pr0c00956_si_005.zip › timstof_data/resources/images/all-samples1-mass2-deviation.png]

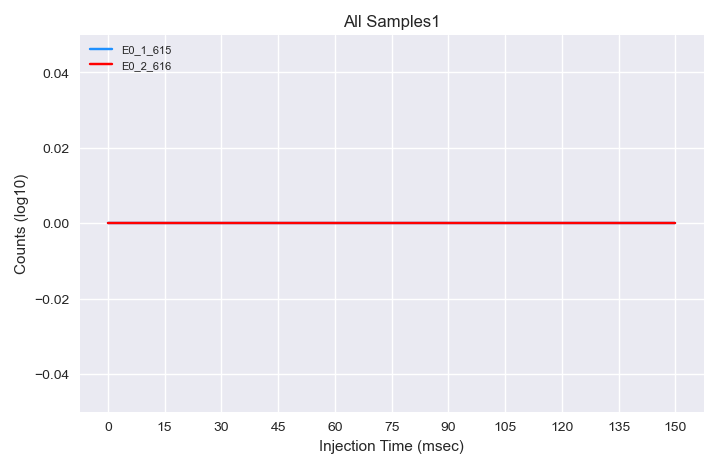

Supplement: Supplementary file 4 — pr0c00956_si_005.zip [file pr0c00956_si_005.zip › timstof_data/resources/images/all-samples1-ms2-inject.png]

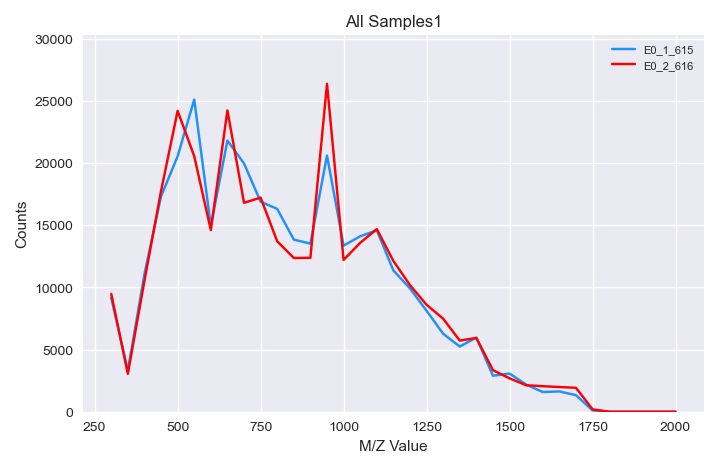

Supplement: Supplementary file 4 — pr0c00956_si_005.zip [file pr0c00956_si_005.zip › timstof_data/resources/images/all-samples1-ms2-mz-value.png]

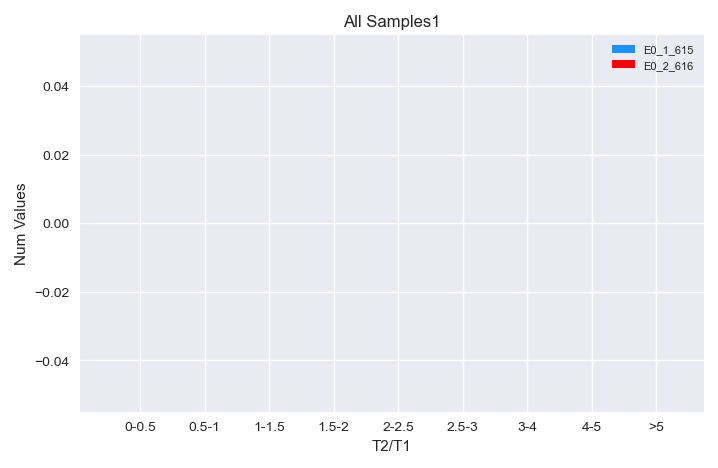

Supplement: Supplementary file 4 — pr0c00956_si_005.zip [file pr0c00956_si_005.zip › timstof_data/resources/images/all-samples1-peak-symmetry.png]

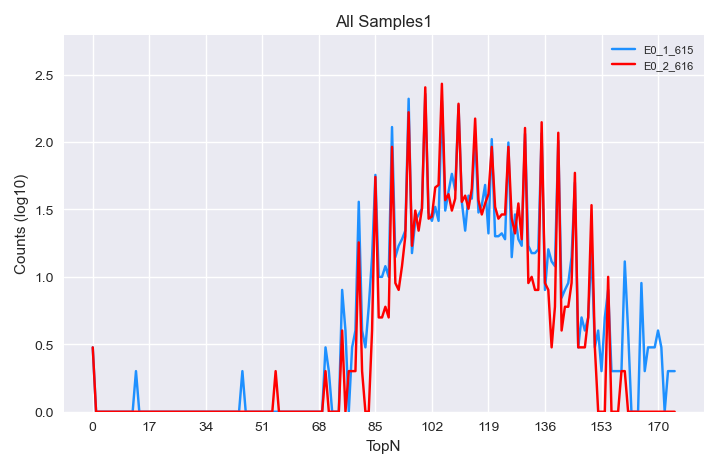

Supplement: Supplementary file 4 — pr0c00956_si_005.zip [file pr0c00956_si_005.zip › timstof_data/resources/images/all-samples1-top-n.png]

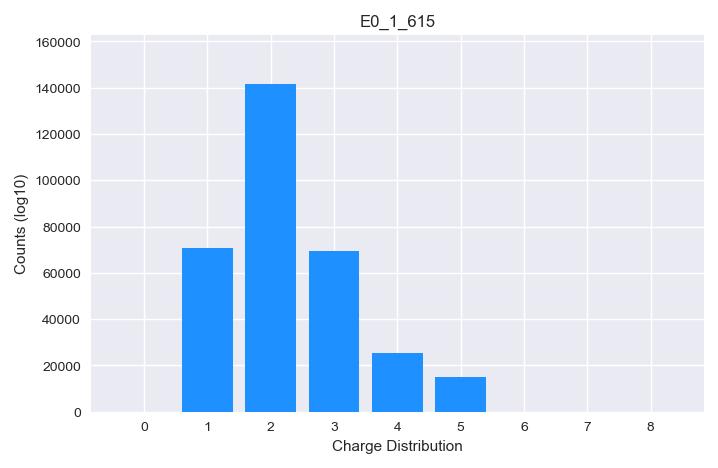

Supplement: Supplementary file 4 — pr0c00956_si_005.zip [file pr0c00956_si_005.zip › timstof_data/resources/images/E0_1_615-charge-state.png]

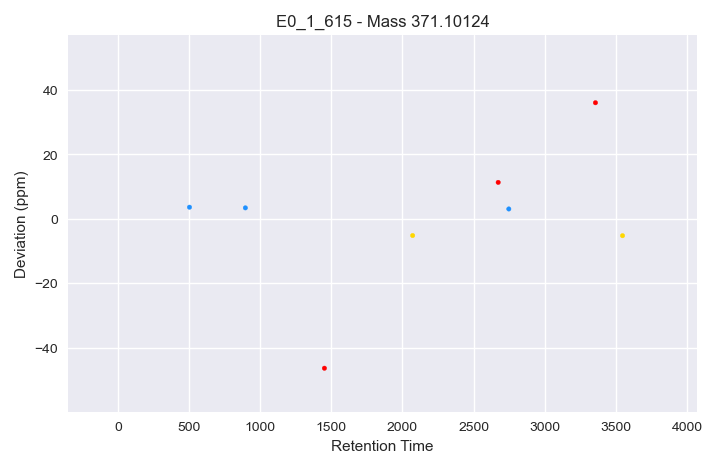

Supplement: Supplementary file 4 — pr0c00956_si_005.zip [file pr0c00956_si_005.zip › timstof_data/resources/images/E0_1_615-mass-deviation1.png]

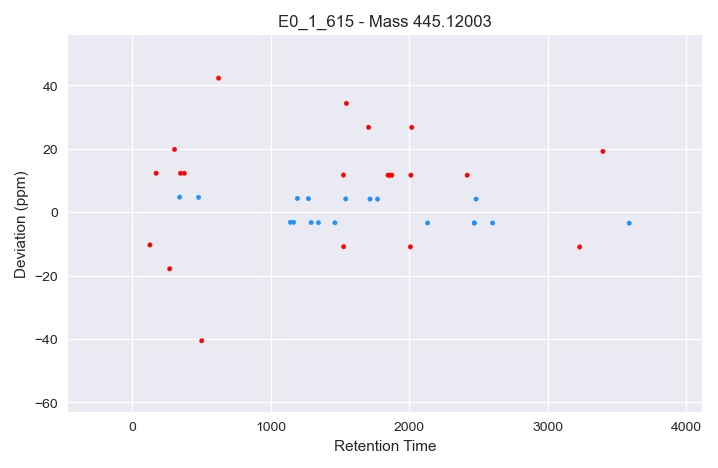

Supplement: Supplementary file 4 — pr0c00956_si_005.zip [file pr0c00956_si_005.zip › timstof_data/resources/images/E0_1_615-mass-deviation2.png]

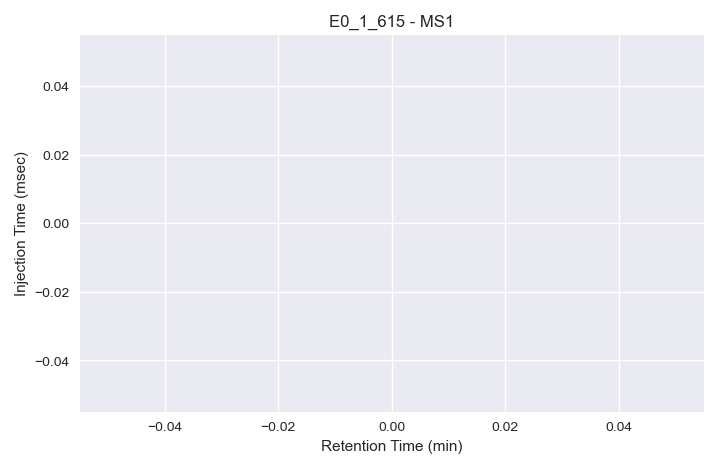

Supplement: Supplementary file 4 — pr0c00956_si_005.zip [file pr0c00956_si_005.zip › timstof_data/resources/images/E0_1_615-ms1-inject-vs-ret.png]

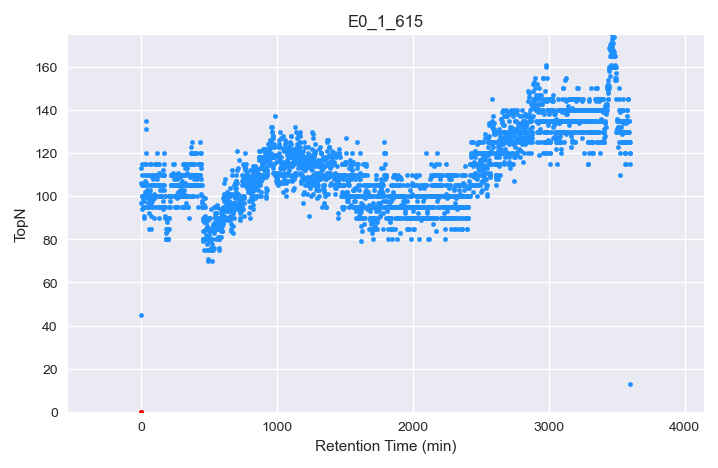

Supplement: Supplementary file 4 — pr0c00956_si_005.zip [file pr0c00956_si_005.zip › timstof_data/resources/images/E0_1_615-ms1-ret-vs-top-n.png]

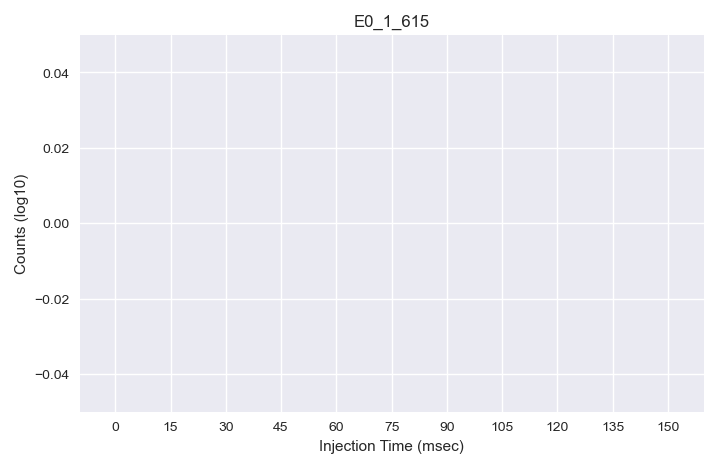

Supplement: Supplementary file 4 — pr0c00956_si_005.zip [file pr0c00956_si_005.zip › timstof_data/resources/images/E0_1_615-ms2-inject.png]

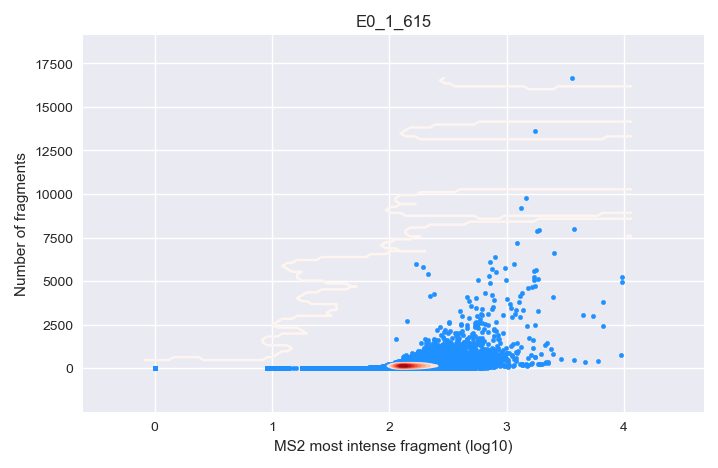

Supplement: Supplementary file 4 — pr0c00956_si_005.zip [file pr0c00956_si_005.zip › timstof_data/resources/images/E0_1_615-ms2-max-log-intensity-vs-ms2-num-intensities.png]

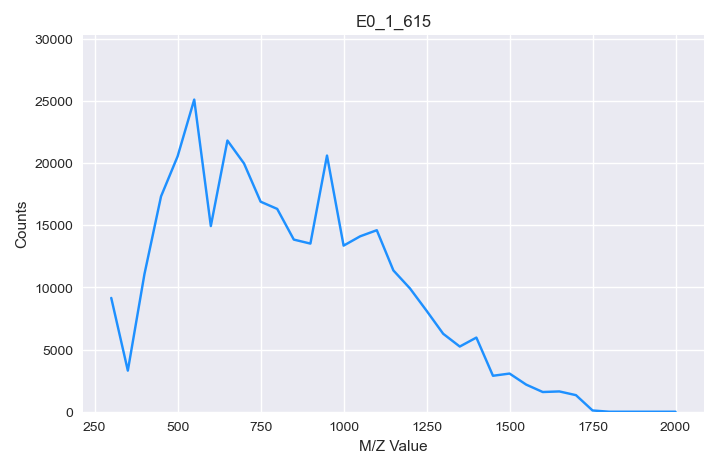

Supplement: Supplementary file 4 — pr0c00956_si_005.zip [file pr0c00956_si_005.zip › timstof_data/resources/images/E0_1_615-ms2-mz-value.png]

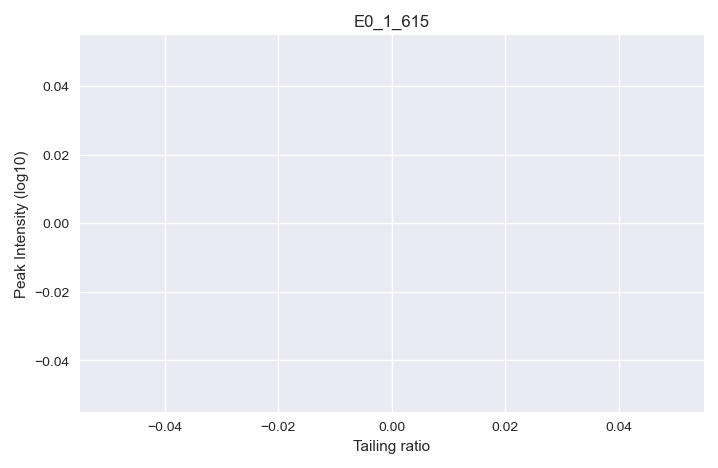

Supplement: Supplementary file 4 — pr0c00956_si_005.zip [file pr0c00956_si_005.zip › timstof_data/resources/images/E0_1_615-peak-intentsity-vs-t2-t1-ratio.png]

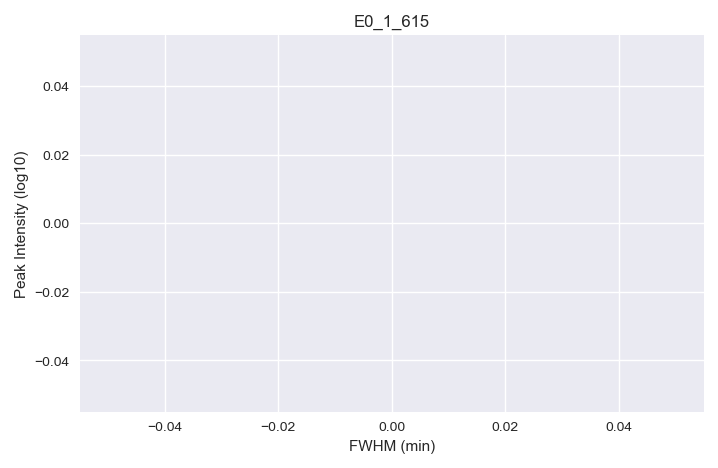

Supplement: Supplementary file 4 — pr0c00956_si_005.zip [file pr0c00956_si_005.zip › timstof_data/resources/images/E0_1_615-peak-intentsity-vs-t-sum.png]

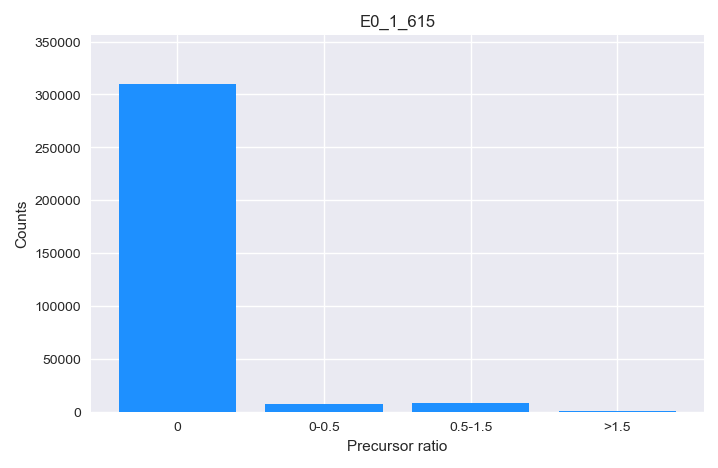

Supplement: Supplementary file 4 — pr0c00956_si_005.zip [file pr0c00956_si_005.zip › timstof_data/resources/images/E0_1_615-prec-ratio.png]

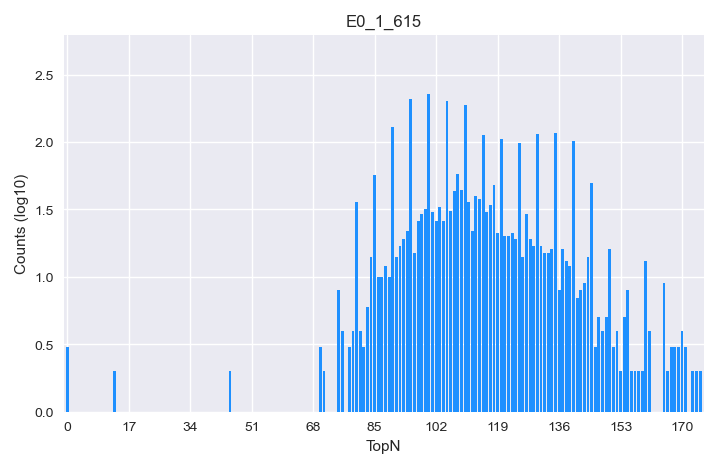

Supplement: Supplementary file 4 — pr0c00956_si_005.zip [file pr0c00956_si_005.zip › timstof_data/resources/images/E0_1_615-top-n.png]

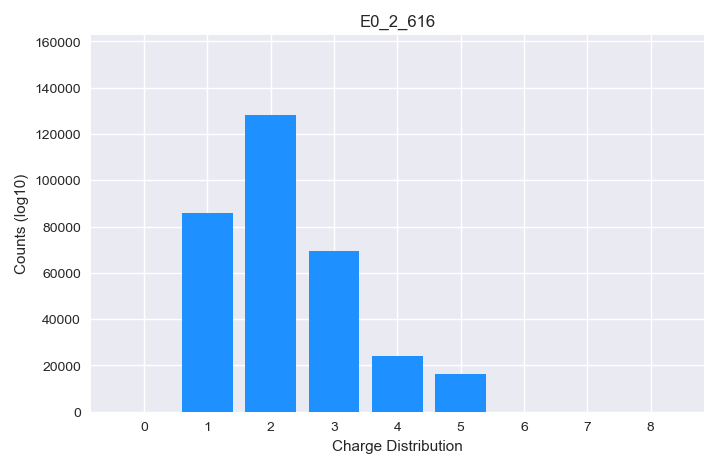

Supplement: Supplementary file 4 — pr0c00956_si_005.zip [file pr0c00956_si_005.zip › timstof_data/resources/images/E0_2_616-charge-state.png]

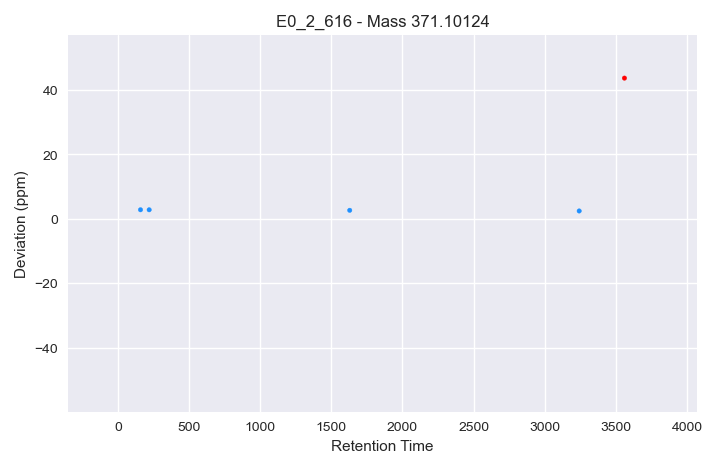

Supplement: Supplementary file 4 — pr0c00956_si_005.zip [file pr0c00956_si_005.zip › timstof_data/resources/images/E0_2_616-mass-deviation1.png]

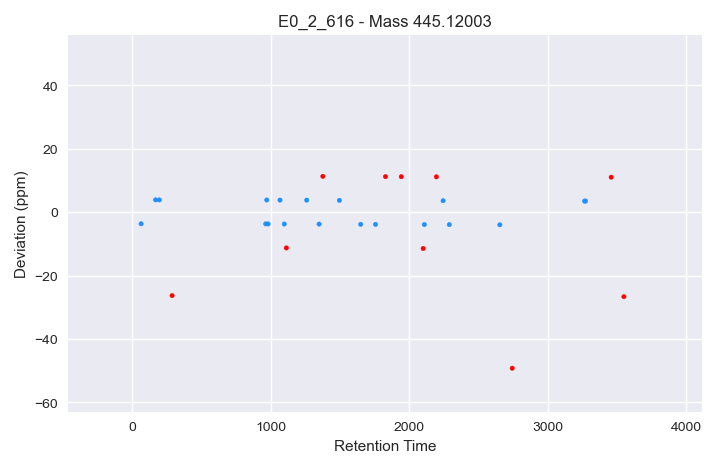

Supplement: Supplementary file 4 — pr0c00956_si_005.zip [file pr0c00956_si_005.zip › timstof_data/resources/images/E0_2_616-mass-deviation2.png]

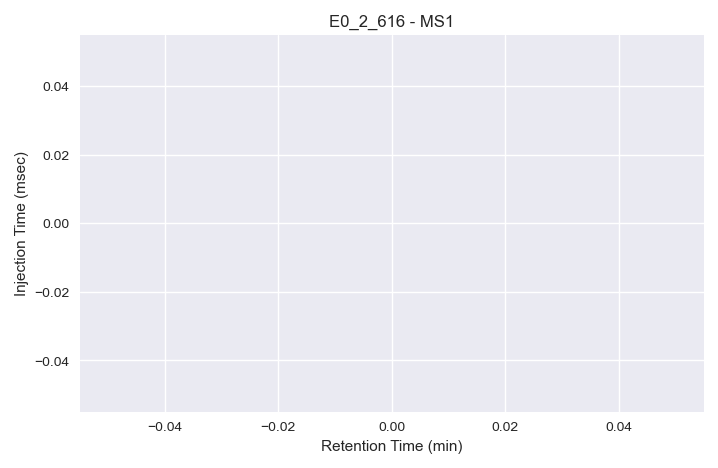

Supplement: Supplementary file 4 — pr0c00956_si_005.zip [file pr0c00956_si_005.zip › timstof_data/resources/images/E0_2_616-ms1-inject-vs-ret.png]

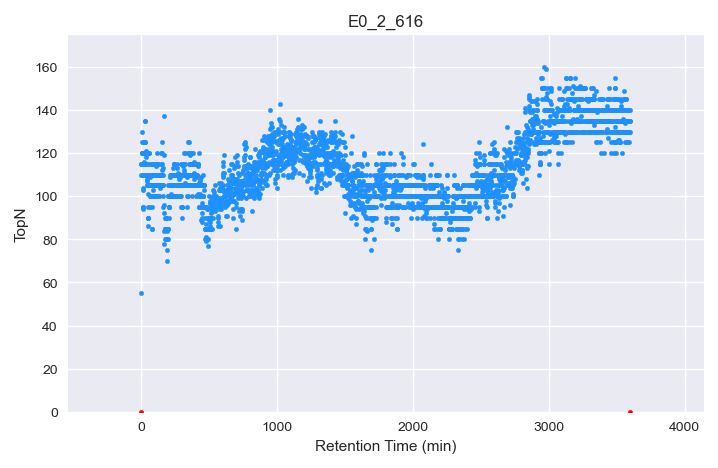

Supplement: Supplementary file 4 — pr0c00956_si_005.zip [file pr0c00956_si_005.zip › timstof_data/resources/images/E0_2_616-ms1-ret-vs-top-n.png]

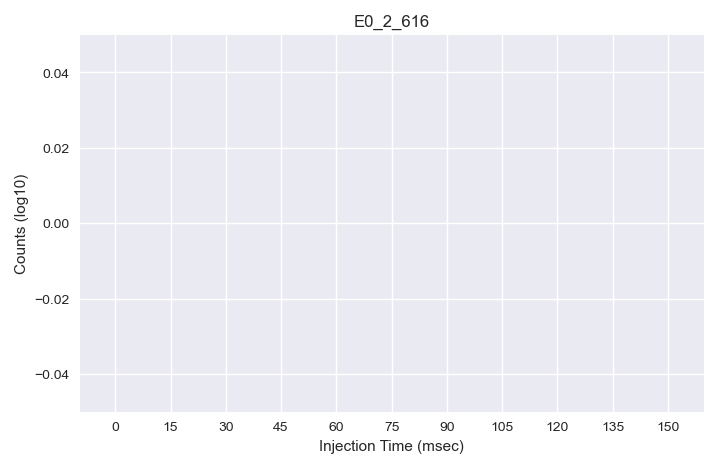

Supplement: Supplementary file 4 — pr0c00956_si_005.zip [file pr0c00956_si_005.zip › timstof_data/resources/images/E0_2_616-ms2-inject.png]

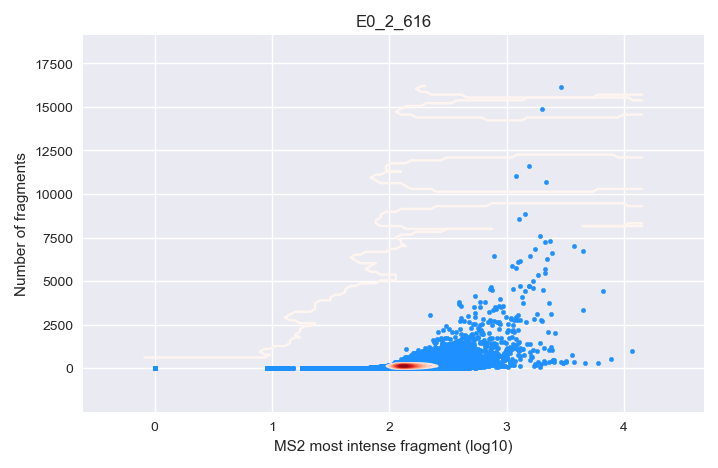

Supplement: Supplementary file 4 — pr0c00956_si_005.zip [file pr0c00956_si_005.zip › timstof_data/resources/images/E0_2_616-ms2-max-log-intensity-vs-ms2-num-intensities.png]

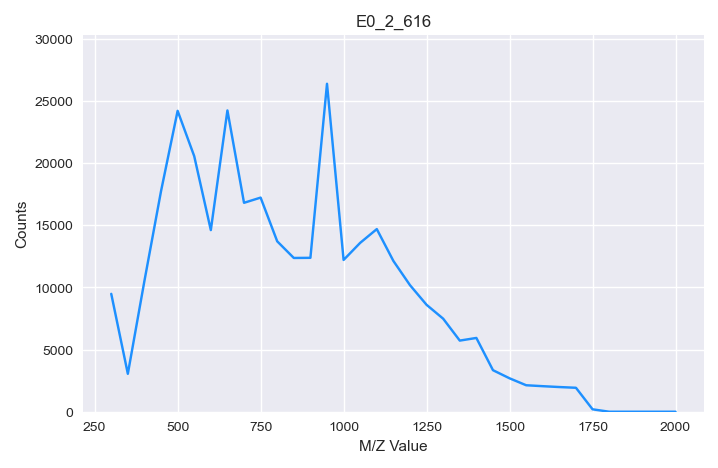

Supplement: Supplementary file 4 — pr0c00956_si_005.zip [file pr0c00956_si_005.zip › timstof_data/resources/images/E0_2_616-ms2-mz-value.png]

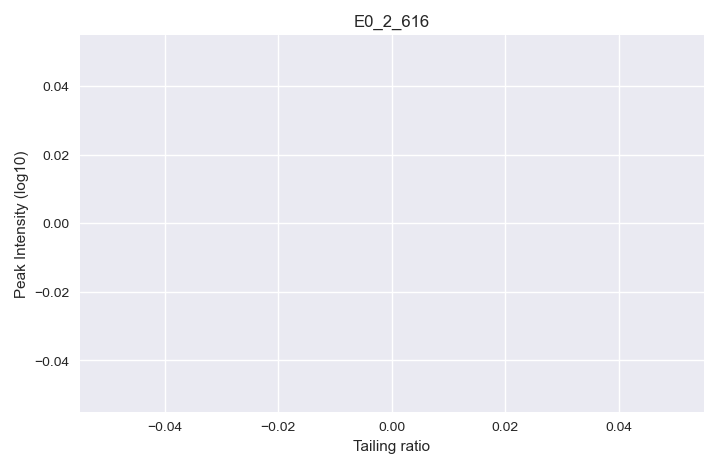

Supplement: Supplementary file 4 — pr0c00956_si_005.zip [file pr0c00956_si_005.zip › timstof_data/resources/images/E0_2_616-peak-intentsity-vs-t2-t1-ratio.png]

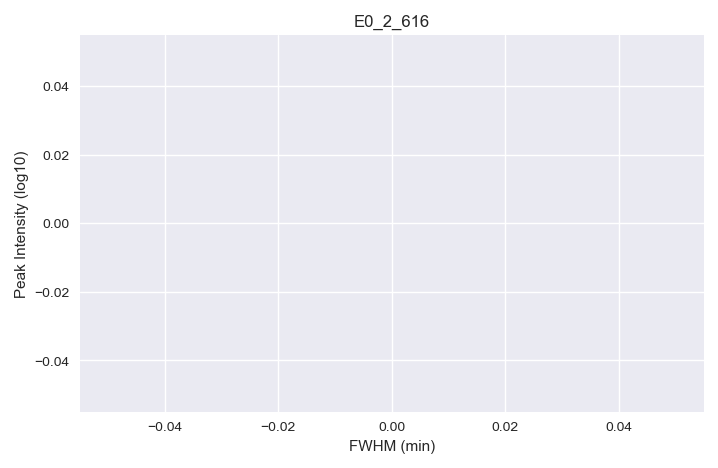

Supplement: Supplementary file 4 — pr0c00956_si_005.zip [file pr0c00956_si_005.zip › timstof_data/resources/images/E0_2_616-peak-intentsity-vs-t-sum.png]

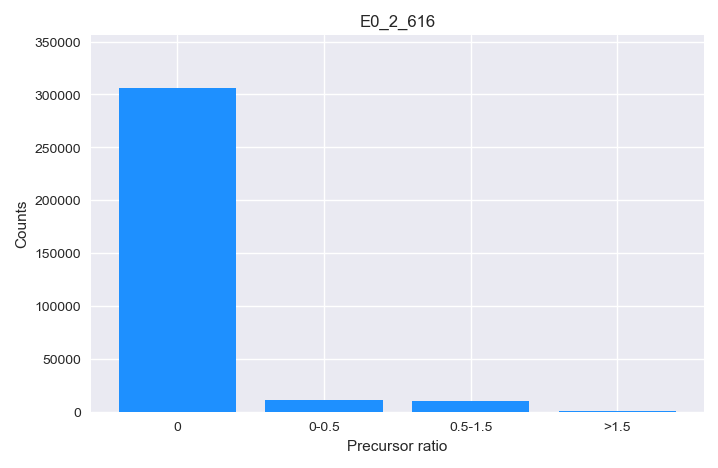

Supplement: Supplementary file 4 — pr0c00956_si_005.zip [file pr0c00956_si_005.zip › timstof_data/resources/images/E0_2_616-prec-ratio.png]

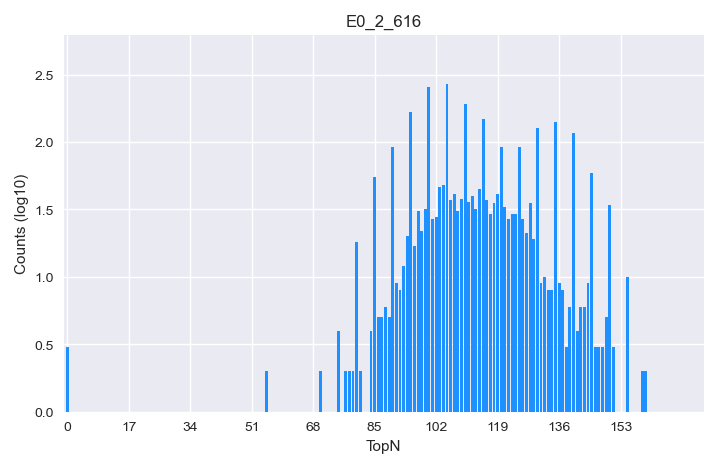

Supplement: Supplementary file 4 — pr0c00956_si_005.zip [file pr0c00956_si_005.zip › timstof_data/resources/images/E0_2_616-top-n.png]

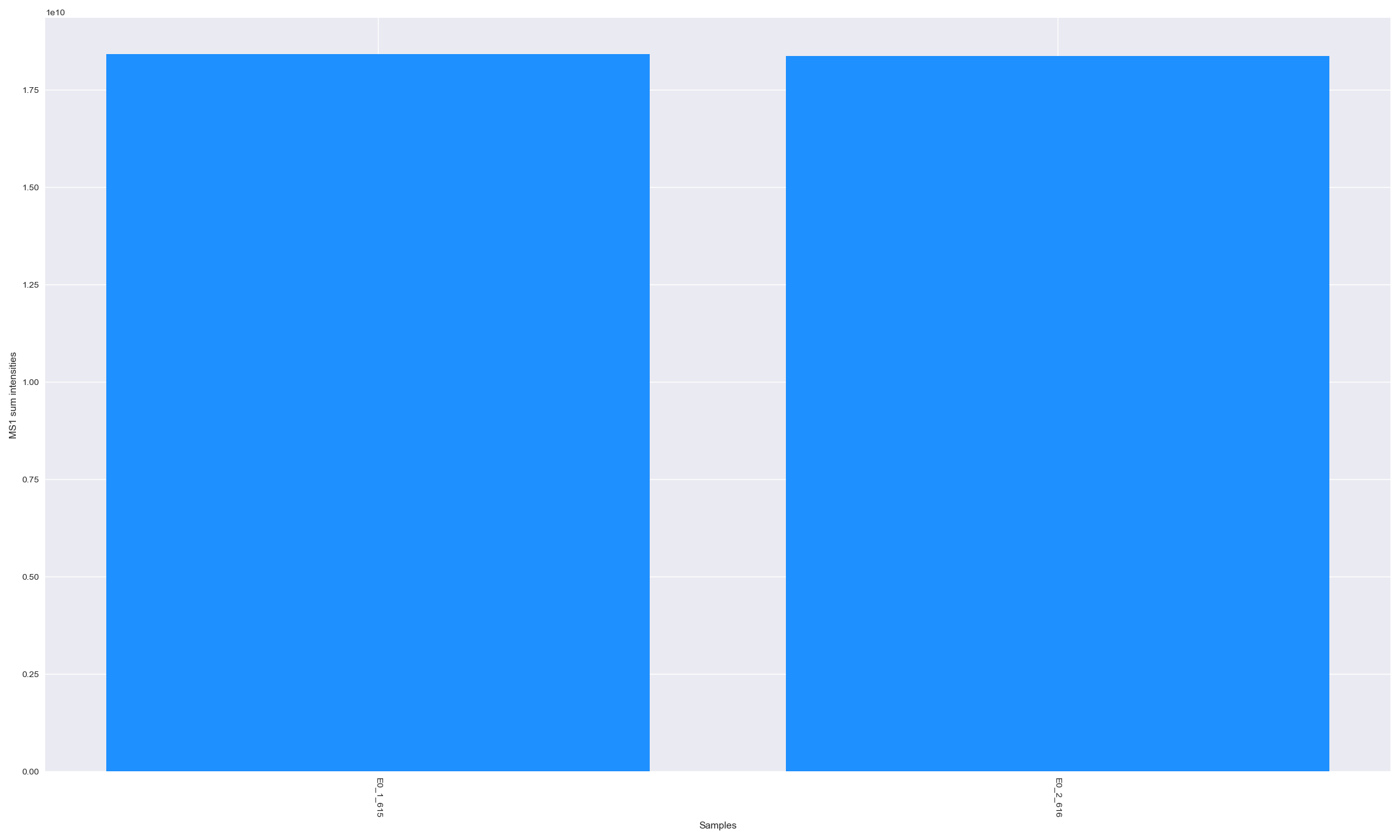

Supplement: Supplementary file 4 — pr0c00956_si_005.zip [file pr0c00956_si_005.zip › timstof_data/resources/images/tic-lex-sort.png]

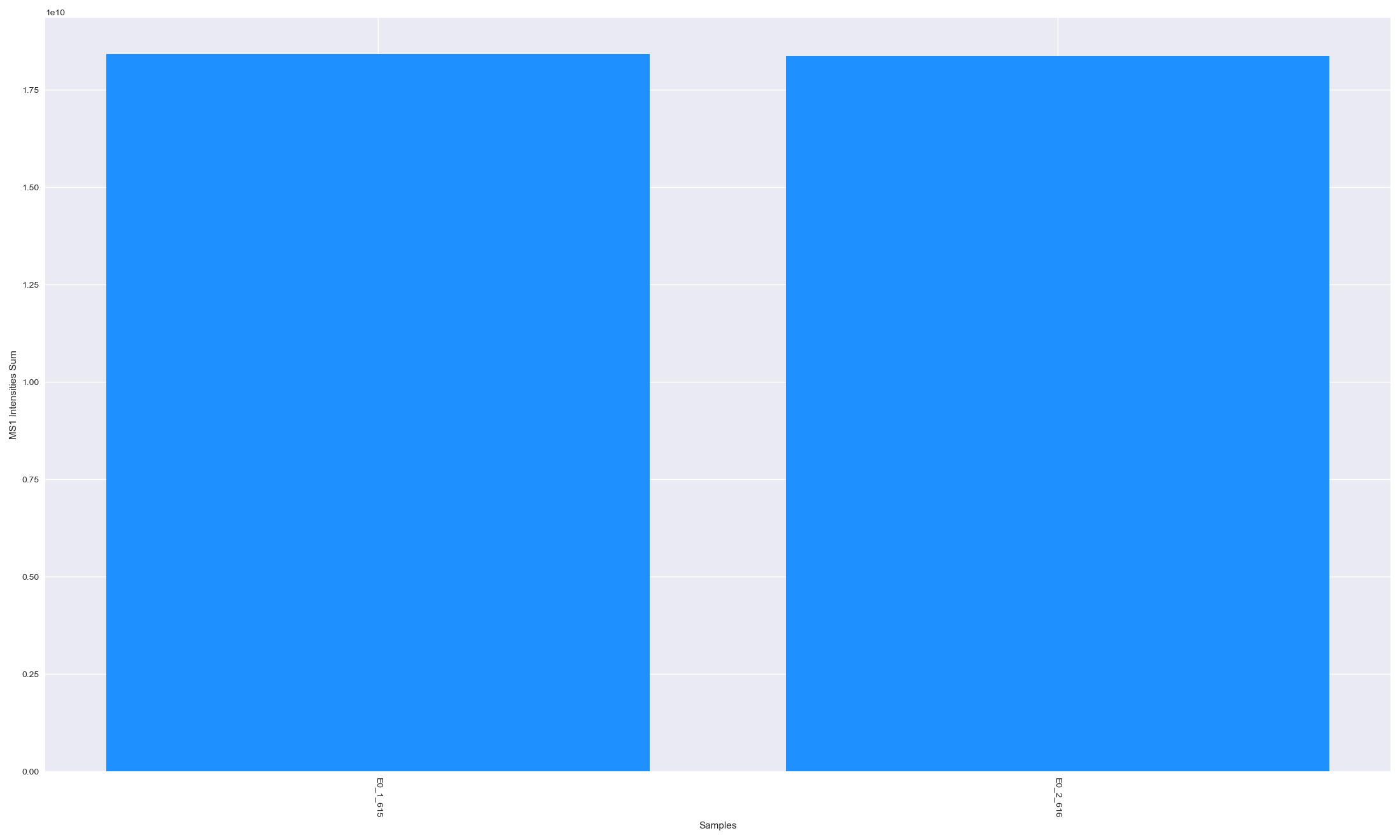

Supplement: Supplementary file 4 — pr0c00956_si_005.zip [file pr0c00956_si_005.zip › timstof_data/resources/images/tic-run-date-sort.png]

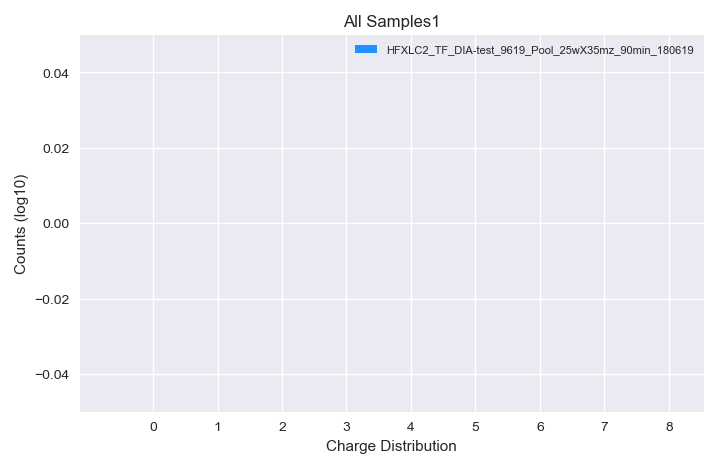

Supplement: Supplementary file 5 — pr0c00956_si_006.zip [file pr0c00956_si_006.zip › DIA/resources/images/all-samples1-charge-state.png]

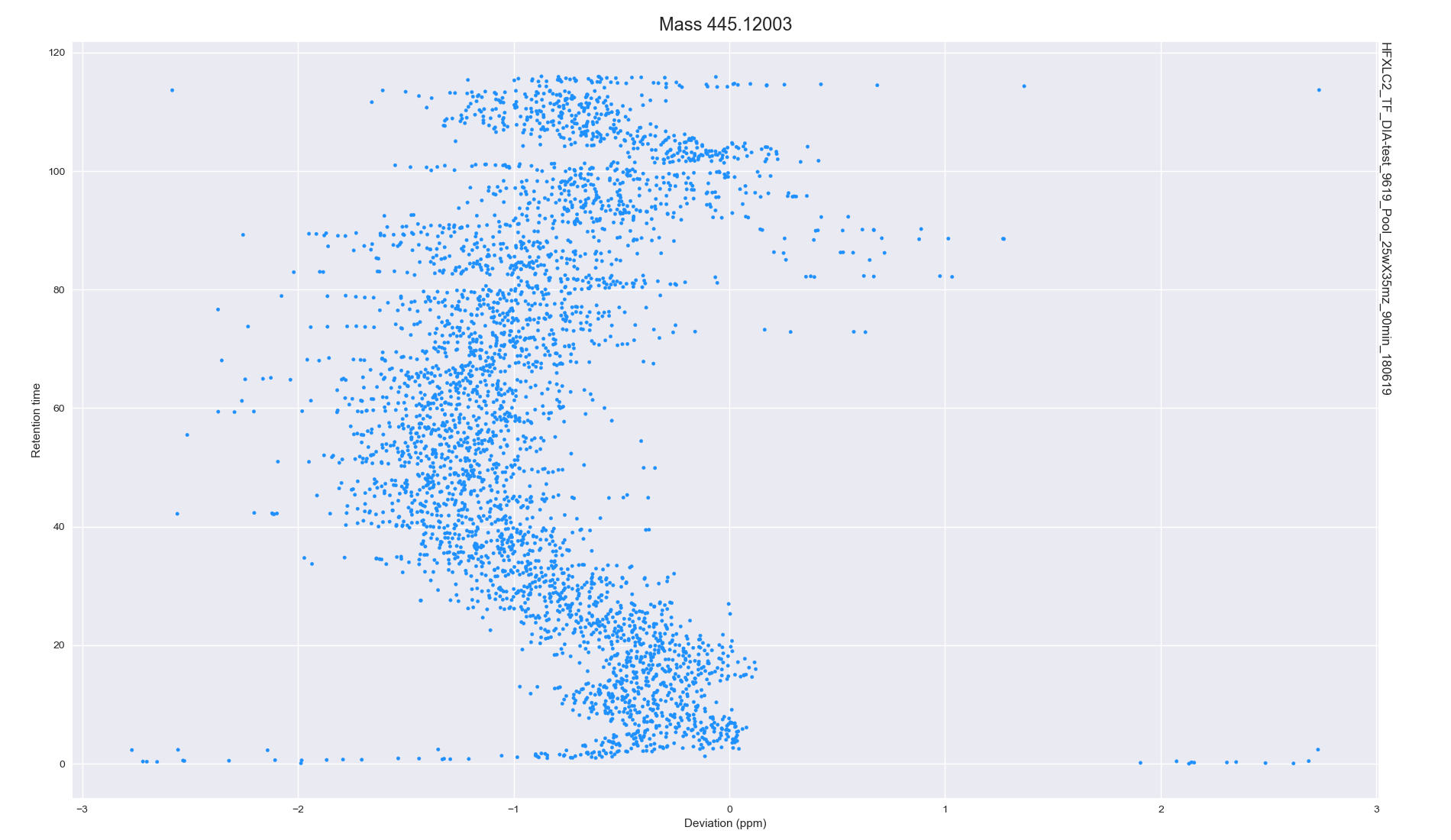

Supplement: Supplementary file 5 — pr0c00956_si_006.zip [file pr0c00956_si_006.zip › DIA/resources/images/all-samples1-mass1-deviation.png]

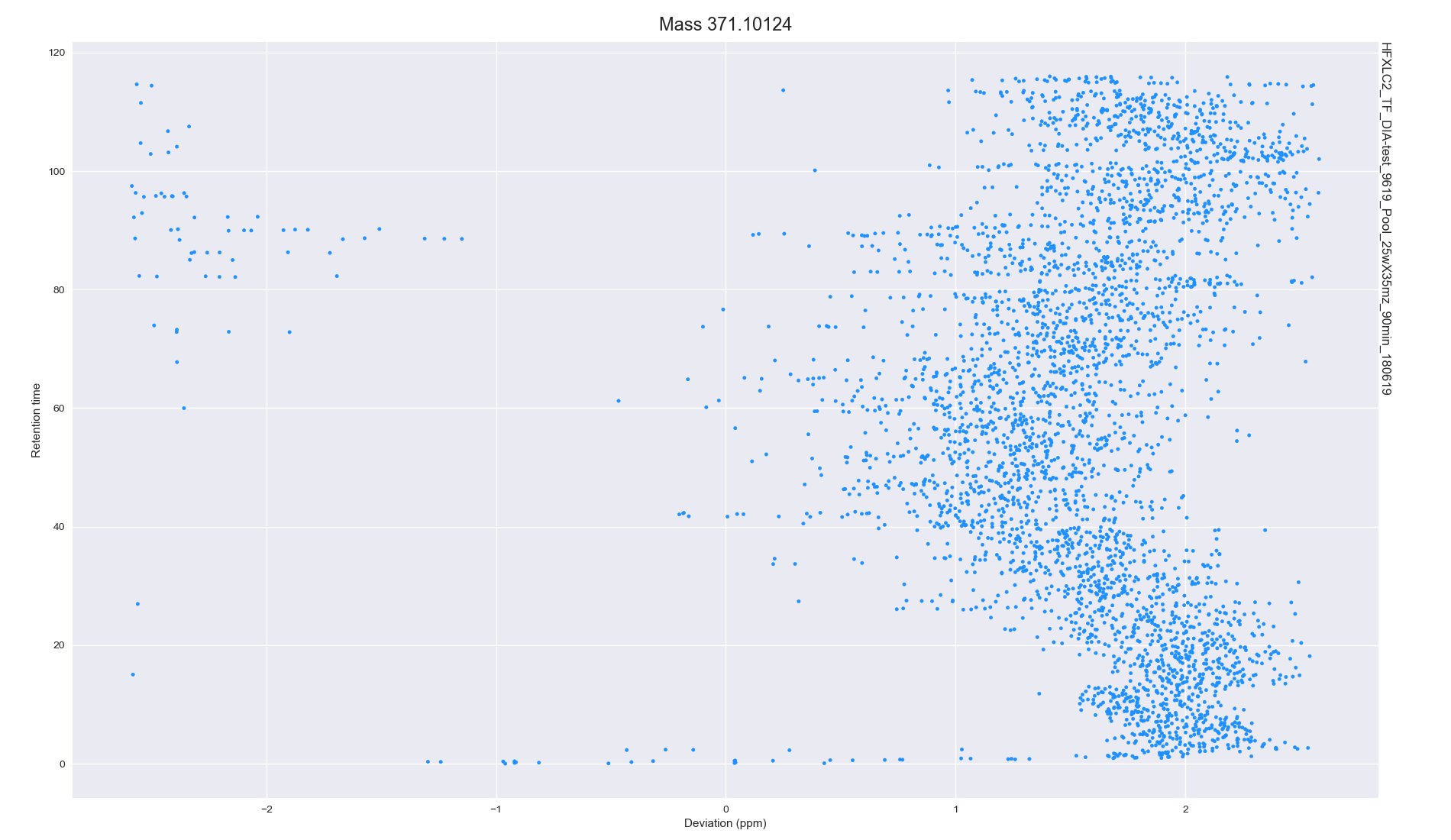

Supplement: Supplementary file 5 — pr0c00956_si_006.zip [file pr0c00956_si_006.zip › DIA/resources/images/all-samples1-mass2-deviation.png]

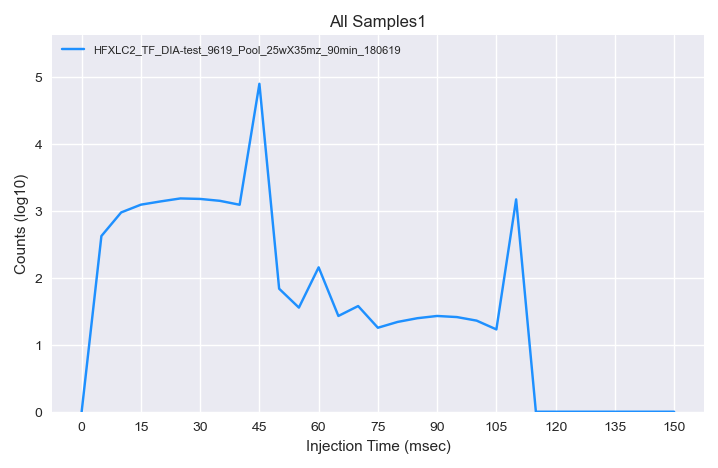

Supplement: Supplementary file 5 — pr0c00956_si_006.zip [file pr0c00956_si_006.zip › DIA/resources/images/all-samples1-ms2-inject.png]

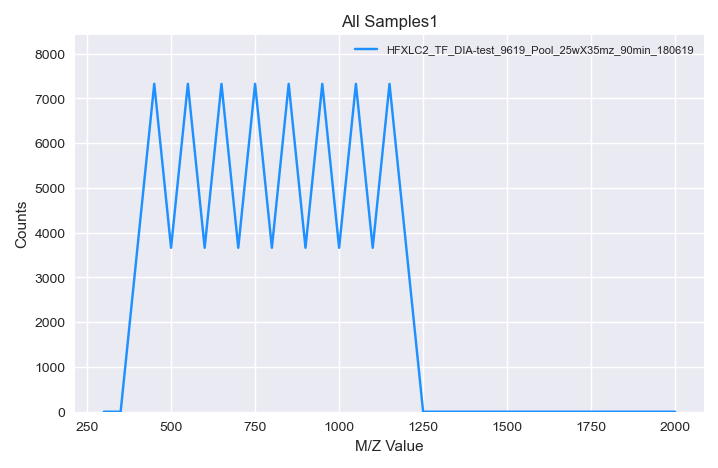

Supplement: Supplementary file 5 — pr0c00956_si_006.zip [file pr0c00956_si_006.zip › DIA/resources/images/all-samples1-ms2-mz-value.png]

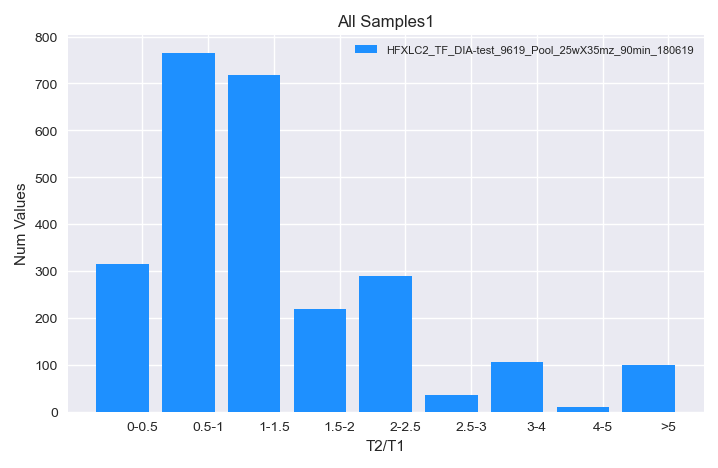

Supplement: Supplementary file 5 — pr0c00956_si_006.zip [file pr0c00956_si_006.zip › DIA/resources/images/all-samples1-peak-symmetry.png]

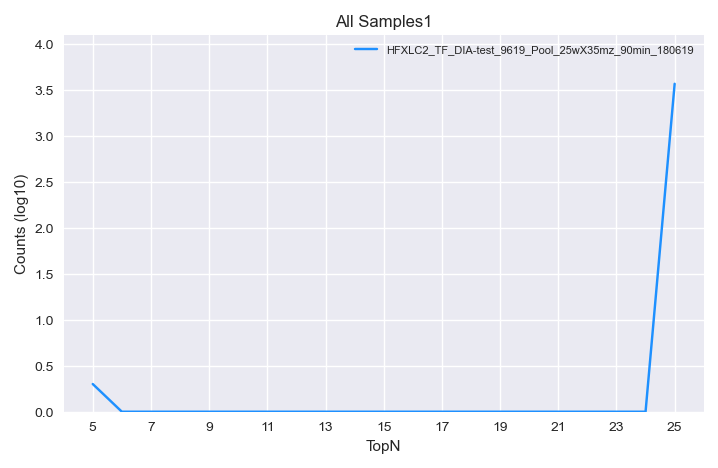

Supplement: Supplementary file 5 — pr0c00956_si_006.zip [file pr0c00956_si_006.zip › DIA/resources/images/all-samples1-top-n.png]

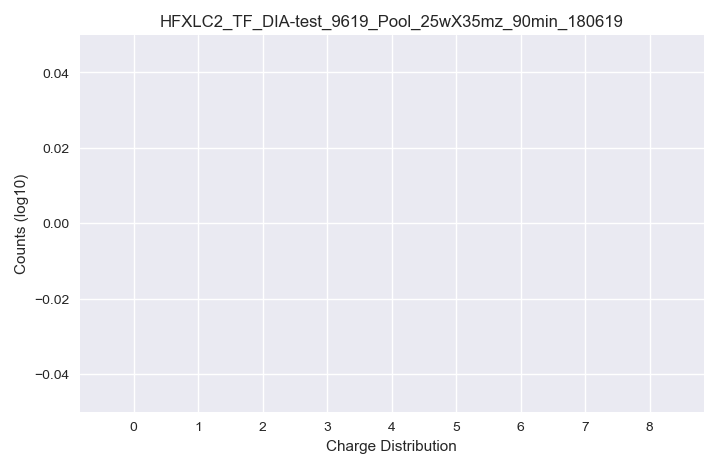

Supplement: Supplementary file 5 — pr0c00956_si_006.zip [file pr0c00956_si_006.zip › DIA/resources/images/HFXLC2_TF_DIA-test_9619_Pool_25wX35mz_90min_180619-charge-state.png]

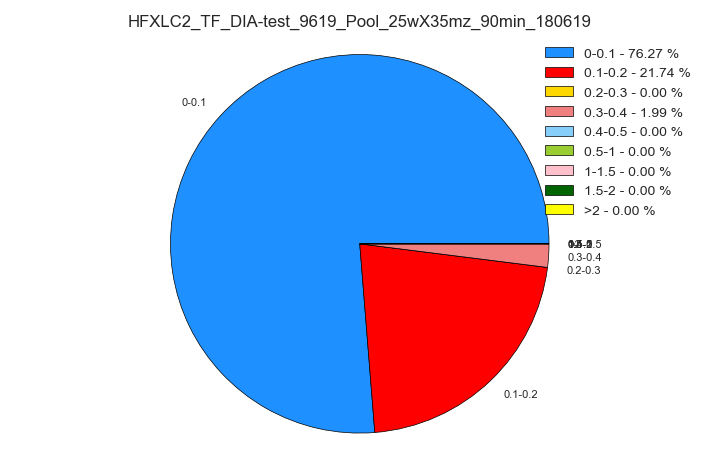

Supplement: Supplementary file 5 — pr0c00956_si_006.zip [file pr0c00956_si_006.zip › DIA/resources/images/HFXLC2_TF_DIA-test_9619_Pool_25wX35mz_90min_180619-fmhw-pie.png]

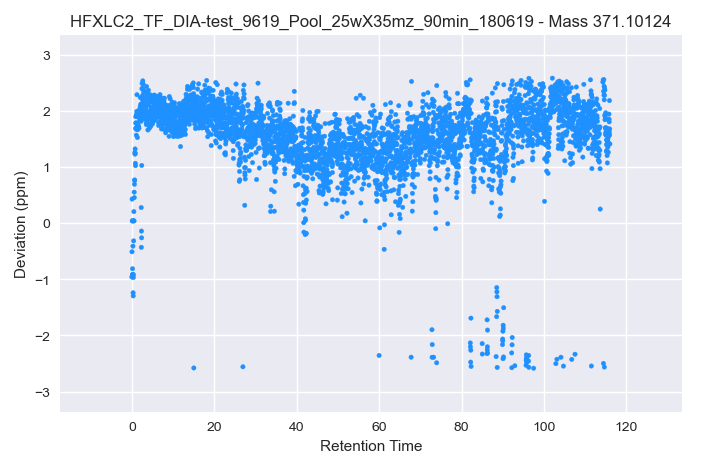

Supplement: Supplementary file 5 — pr0c00956_si_006.zip [file pr0c00956_si_006.zip › DIA/resources/images/HFXLC2_TF_DIA-test_9619_Pool_25wX35mz_90min_180619-mass-deviation1.png]

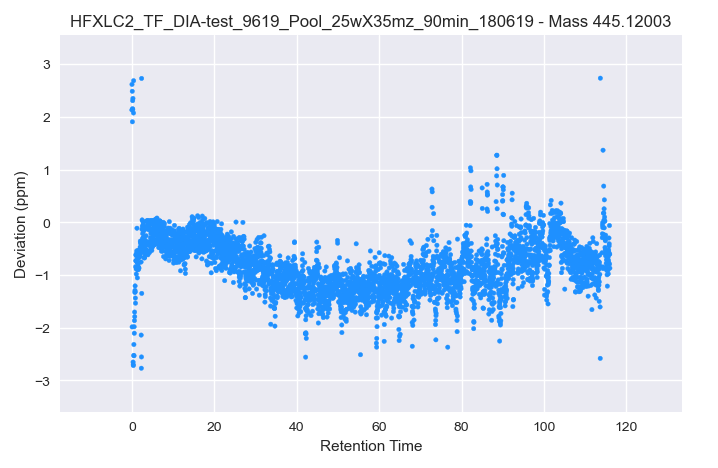

Supplement: Supplementary file 5 — pr0c00956_si_006.zip [file pr0c00956_si_006.zip › DIA/resources/images/HFXLC2_TF_DIA-test_9619_Pool_25wX35mz_90min_180619-mass-deviation2.png]

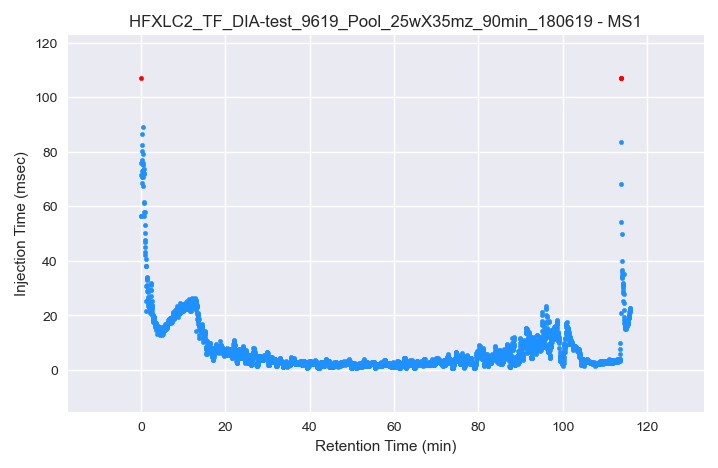

Supplement: Supplementary file 5 — pr0c00956_si_006.zip [file pr0c00956_si_006.zip › DIA/resources/images/HFXLC2_TF_DIA-test_9619_Pool_25wX35mz_90min_180619-ms1-inject-vs-ret.png]

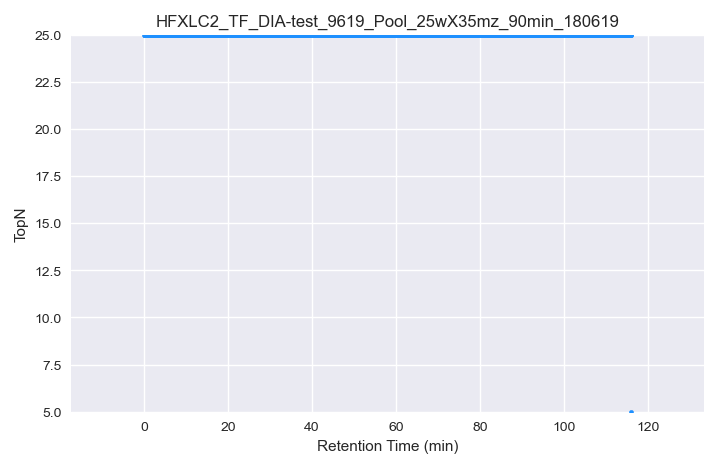

Supplement: Supplementary file 5 — pr0c00956_si_006.zip [file pr0c00956_si_006.zip › DIA/resources/images/HFXLC2_TF_DIA-test_9619_Pool_25wX35mz_90min_180619-ms1-ret-vs-top-n.png]

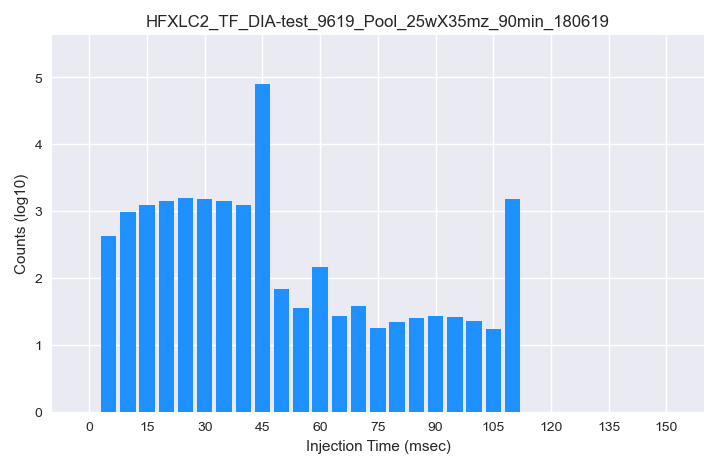

Supplement: Supplementary file 5 — pr0c00956_si_006.zip [file pr0c00956_si_006.zip › DIA/resources/images/HFXLC2_TF_DIA-test_9619_Pool_25wX35mz_90min_180619-ms2-inject.png]

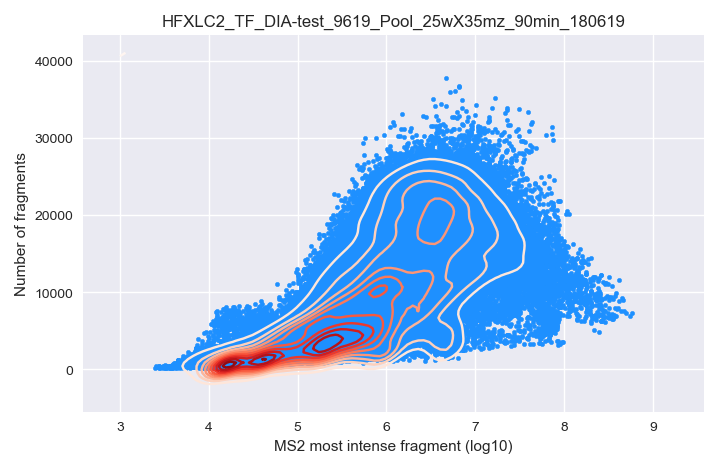

Supplement: Supplementary file 5 — pr0c00956_si_006.zip [file pr0c00956_si_006.zip › DIA/resources/images/HFXLC2_TF_DIA-test_9619_Pool_25wX35mz_90min_180619-ms2-max-log-intensity-vs-ms2-num-intensities.png]

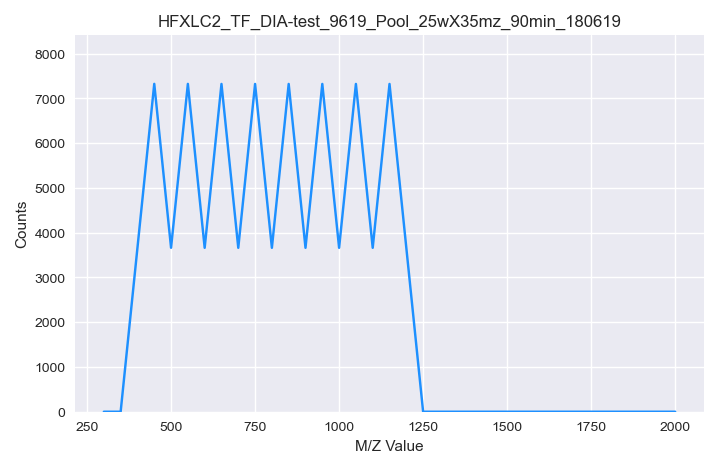

Supplement: Supplementary file 5 — pr0c00956_si_006.zip [file pr0c00956_si_006.zip › DIA/resources/images/HFXLC2_TF_DIA-test_9619_Pool_25wX35mz_90min_180619-ms2-mz-value.png]

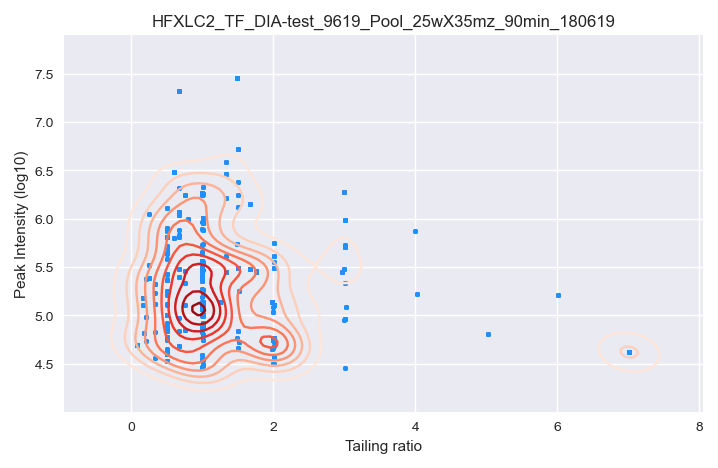

Supplement: Supplementary file 5 — pr0c00956_si_006.zip [file pr0c00956_si_006.zip › DIA/resources/images/HFXLC2_TF_DIA-test_9619_Pool_25wX35mz_90min_180619-peak-intentsity-vs-t2-t1-ratio.png]

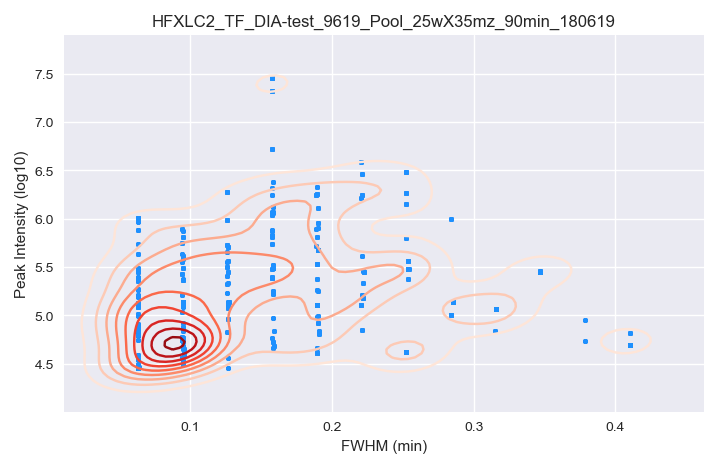

Supplement: Supplementary file 5 — pr0c00956_si_006.zip [file pr0c00956_si_006.zip › DIA/resources/images/HFXLC2_TF_DIA-test_9619_Pool_25wX35mz_90min_180619-peak-intentsity-vs-t-sum.png]

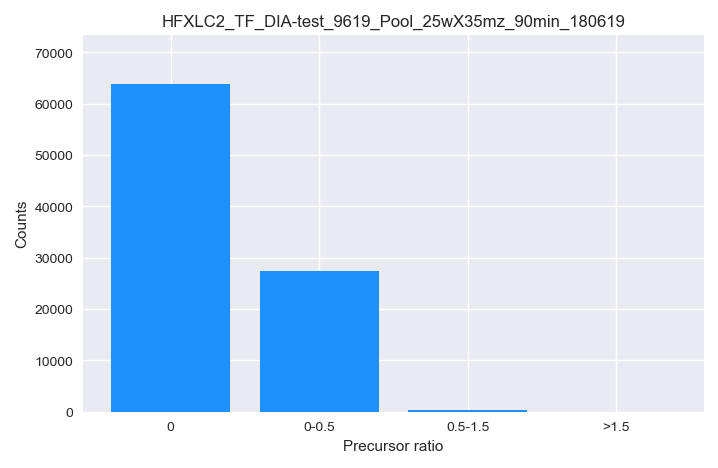

Supplement: Supplementary file 5 — pr0c00956_si_006.zip [file pr0c00956_si_006.zip › DIA/resources/images/HFXLC2_TF_DIA-test_9619_Pool_25wX35mz_90min_180619-prec-ratio.png]

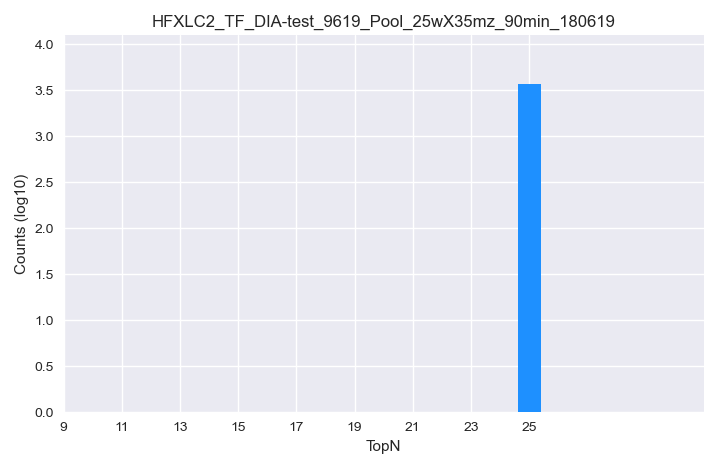

Supplement: Supplementary file 5 — pr0c00956_si_006.zip [file pr0c00956_si_006.zip › DIA/resources/images/HFXLC2_TF_DIA-test_9619_Pool_25wX35mz_90min_180619-top-n.png]

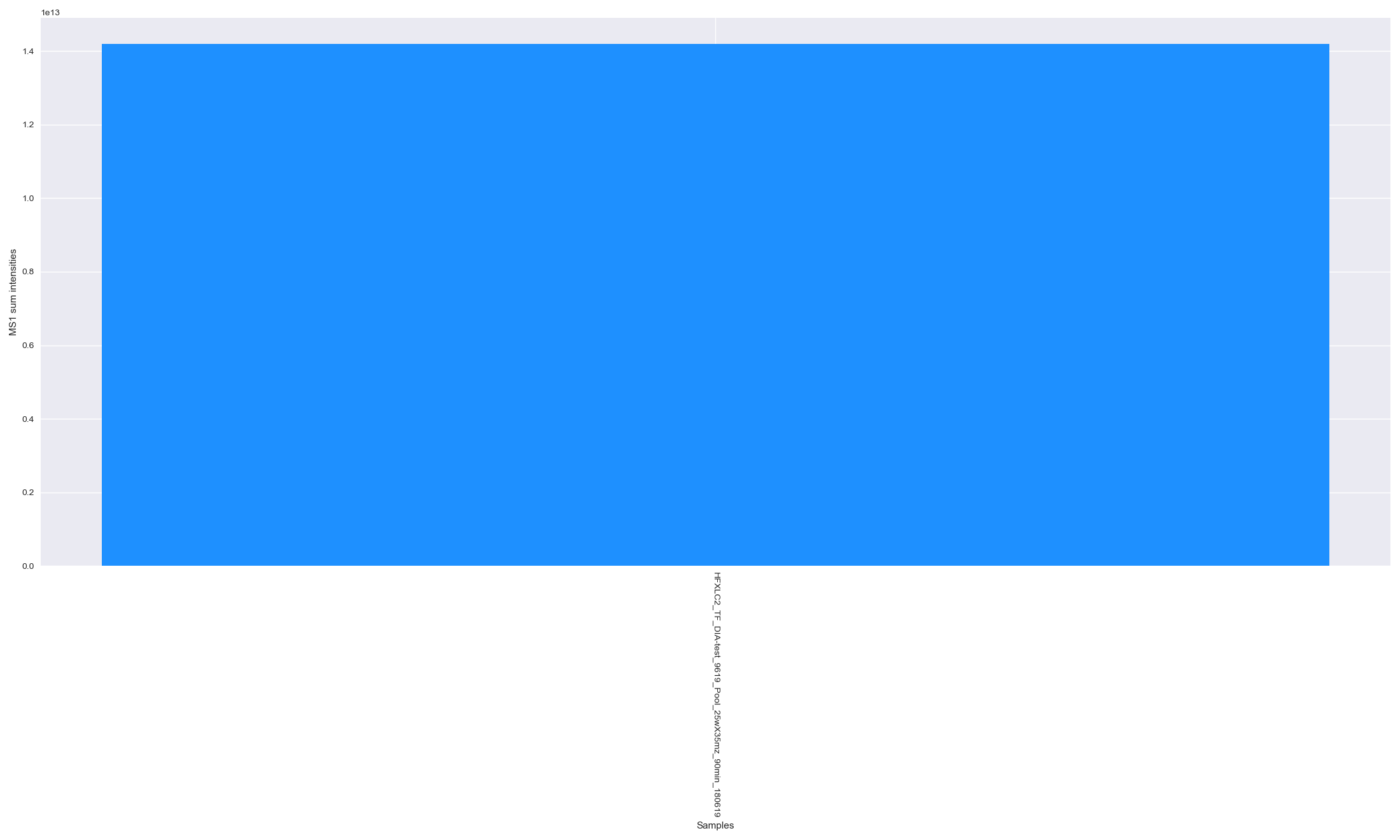

Supplement: Supplementary file 5 — pr0c00956_si_006.zip [file pr0c00956_si_006.zip › DIA/resources/images/tic-lex-sort.png]

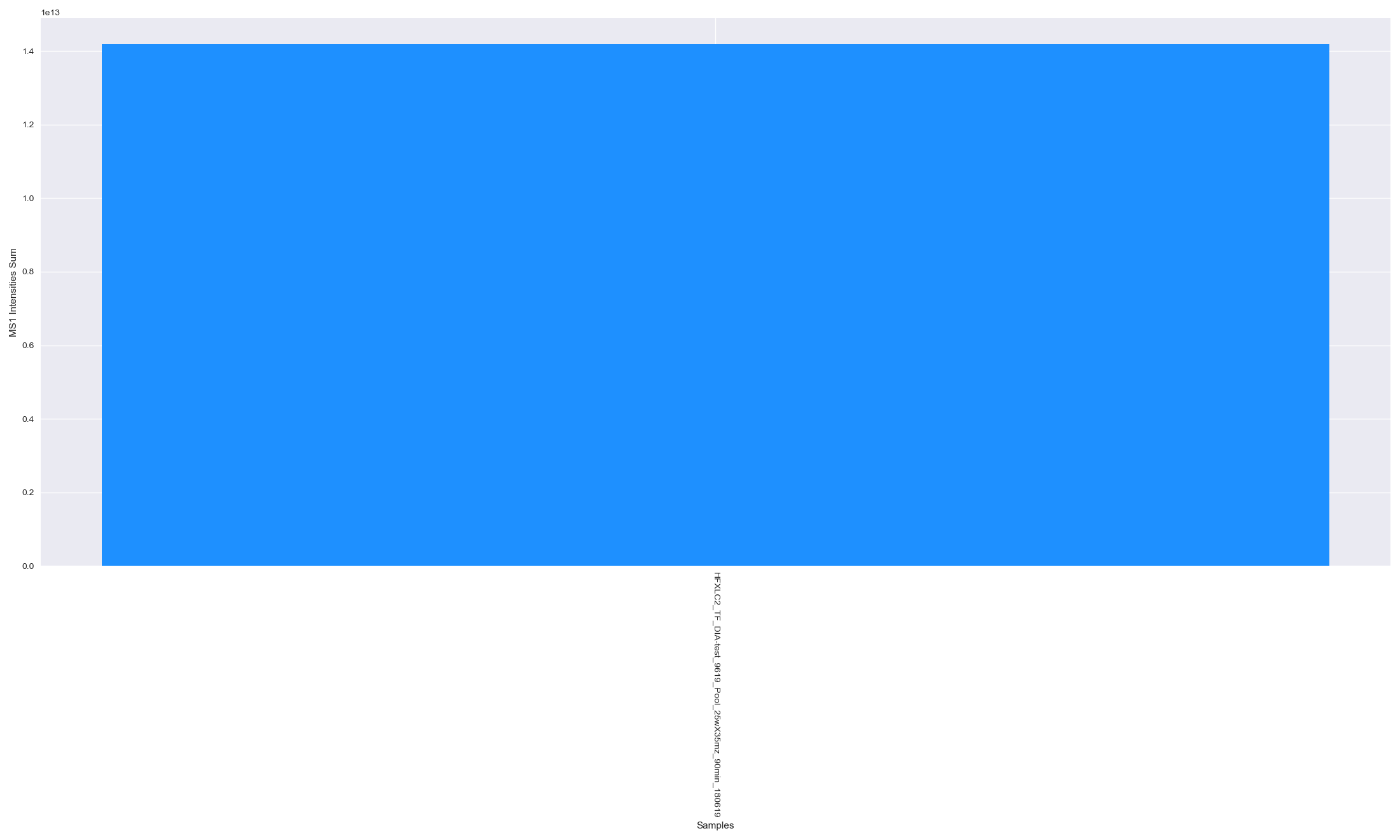

Supplement: Supplementary file 5 — pr0c00956_si_006.zip [file pr0c00956_si_006.zip › DIA/resources/images/tic-run-date-sort.png]
